# Discovery of New Antibacterial Accramycins from a Genetic Variant of the Soil Bacterium, *Streptomyces* sp. MA37

**DOI:** 10.3390/biom10101464

**Published:** 2020-10-20

**Authors:** Fleurdeliz Maglangit, Yuting Zhang, Kwaku Kyeremeh, Hai Deng

**Affiliations:** 1 Department of Chemistry, University of Aberdeen, Aberdeen AB24 3UE, Scotland, UK; y.zhang7.18@abdn.ac.uk; 2 Department of Biology and Environmental Science, College of Science, University of the Philippines Cebu, Lahug, Cebu City 6000, Philippines; 3 Department of Chemistry, University of Ghana, P.O. Box LG56 Legon-Accra, Ghana; kkyeremeh@ug.edu.gh

**Keywords:** accramycin, type II polyketides, *Streptomyces* sp. MA37, regulatory genes, gene inactivation, titer improvement, antibacterial activities, multidrug resistant *Enterococcus*

## Abstract

Continued mining of natural products from the strain *Streptomyces* sp. MA37 in our laboratory led to the discovery of a minor specialized metabolite (SM) called accramycin A. Owing to its low yield (0.2 mg/L) in the wild type strain, we investigated the roles of regulatory genes in the corresponding biosynthetic gene cluster (*acc* BGC) through gene inactivation with the aim of improving the titer of this compound. One of the resulting mutants (∆*accJ*) dramatically upregulated the production of accramycin A **1** by 330-fold (66 mg/L). Furthermore, ten new metabolites, accramycins B–K **2**–**11**, were discovered, together with two known compounds, naphthacemycin B_1_ **12** and fasamycin C **13** from the mutant extract. This suggested that *accJ*, annotated as multiple antibiotic resistance regulator (MarR), is a negative regulator gene in the accramycin biosynthesis. Compounds **1**–**13** inhibited the Gram-positive pathogens (*Staphylococcus aureus, Enterococcus faecalis*) and clinical isolates *Enterococcus faecium* (K59-68 and K60-39) and *Staphylococcus haemolyticus* with minimal inhibitory concentration (MIC) values in the range of 1.5–12.5 µg/mL. Remarkably, compounds **1**–**13** displayed superior activity against K60-39 (MIC = 3.1–6.3 µg/mL) compared to ampicillin (MIC = 25 µg/mL), and offered promising potential for the development of accramycin-based antibiotics that target multidrug-resistant *Enterococcus* clinical isolates. Our results highlight the importance of identifying the roles of regulatory genes in natural product discovery.

## 1. Introduction

**Figure 1:**
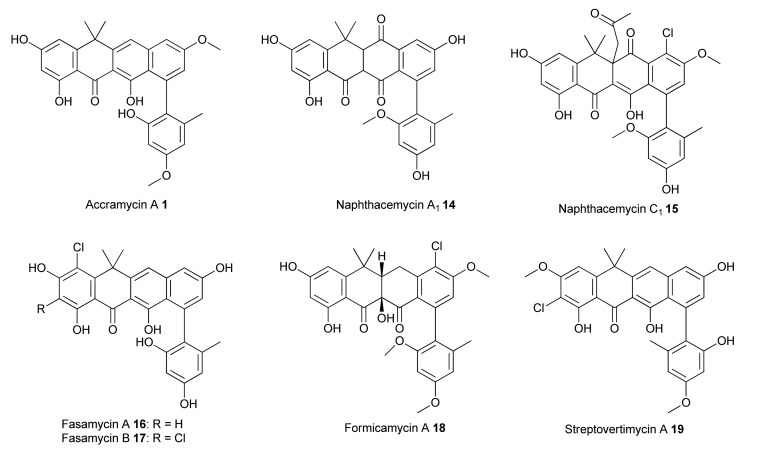
Structures of naphthacemycin-related antibiotics.

Naphthacemycins and congeners are a class of rare aromatic polyketides consisting of a partially reduced 1-phenyltetracene pentacyclic core [1,2,3]. This group of specialized metabolites (SM) display potent activity against various multidrug-resistant Gram-positive pathogens, such as methicillin-resistant *Staphylococcus aureus* (MRSA) and vancomycin-resistant *Enterococcus faecalis* (VRE) [4]. This can be exemplified by the recently discovered naphthacemycin congeners, fasamycins [4,5,6], formicamycins [4], and streptovertimycins [7] (Figure 1). It has been shown that fasamycin A inhibits type II fatty acid synthases (FASII) essential for bacterial cell viability and displays a potent inhibitory effect against FASII in vitro with low IC_50_ value (50 µg/mL) [5]. Due to their potent antibacterial activities, fasamycins have attracted attention to medicinal chemists to perform total synthesis and study the structure–activity relationship of the fasamycin scaffold [8,9,10]. 

During our natural product screening program, we found that the soil isolate, *Streptomyces* sp. MA37, is a highly prolific producer of SMs with great chemical diversity, for example, pyrrolizidines [11], carbazoles [12,13,14,15,16], siderophores [17,18], and fluorinated compounds [19,20,21,22,23,24]. More recently, we discovered a new fasamycin congener, accramycin A **1**, as one of the minor SMs in the MA37 wild type (WT) strain (0.2 mg/L) [25]. Two mono-chlorinated accramycin derivatives were also tentatively assigned via molecular network analysis. Both derivatives, however, could not be isolated due to their minute quantities in the extract [25].

Based on the previous knowledge of fasamycins and formicamycins [4], the putative biosynthetic gene cluster (*acc* BGC) of **1** was identified [25]. The biosynthetic enzymes encoded in the *acc* BGC display high amino acid (AA) similarity with the ones in fasamycins and formicamycins, including the FAD-dependent halogenation enzyme (AccV) which shares high AA identity with the chlorinase, ForV, encoded in the fasamycin and formicamycin BGCs from *Streptomyces formicae* [4]. Unlike *S. formicae*, which produces an array of mono-, di-, and tri-chlorinated fasamycins and formicamycins, the MA37 WT strain appears to be less productive in terms of accramycin yield and chemical diversity, suggesting poor expression of the *acc* BGC in the MA37 WT strain [25]. It was hypothesized that this may be due to the presence of negative regulatory genes that suppress the biosynthesis of accramycins.

Herein, we report the hyper accramycin producer of an MA37 variant by inactivating the putative regulatory gene (*accJ*), which encodes for the multiple antibiotic resistance regulator (MarR). Subsequent chemical workup and structural elucidation allowed for the discovery of more than ten new accramycin congeners, together with two known compounds, naphthacemycin B_1_ **12** [26] and fasamycin C **13** [4]. Except for compounds **3** and **4**, compounds **5**–**11** contain multi-chlorines installed at various positions of the accramycin scaffold, suggesting that the putative enzyme (AccV) is a promiscuous chlorinase. A similar observation was observed for the deletion of the *forJ* gene (*accJ* homologue) in *S. formicae*, which also resulted in improved titer of fasamycins and formicamycins [27], in parallel to our study. 

## 2. Materials and Methods

### 2.1. General Experimental Procedures

Agilent 1260 Infinity (Scotland, UK) was used for high pressure liquid chromatography (HPLC) separation. IR spectra were obtained using a PerkinElmer Spectrum version 10.4.00 Fourier transform infrared (FTIR) spectrometer (2013) (Scotland, UK) equipped with an attenuated total reflection (ATR) diamond cell. HR-ESIMS were determined on an LC-MS Thermal Science Mass Spectrometry (LTQ Orbitrap, Scotland, UK) coupled with a thermal instrument HPLC (Accela PDA detector, Accela PDA autosampler and Accela Pump, C18 Sunfire 4.6 × 150 mm waters, Scotland, UK). The NMR spectra were recorded on a Bruker AVANCE III HD 400 MHz (Ascend™ 9.4 Tesla) and/or Bruker AVANCE III HD 600 MHz (Ascend™ 14.1 Tesla, Scotland, UK) with Prodigy TCITM cryoprobe.

### 2.2. Strain, Genomic DNA, and Media

The *Streptomyces* sp. MA37 strain was isolated from the soil sample collected from Legon, Ghana, Africa [20]. Genomic DNA from MA37 was extracted as described previously [24]. All media/broth for fermentation, solvents, and chemicals were obtained from Fisher Scientific (Scotland, UK), unless otherwise stated.

### 2.3. Construction of Knockout Vector

The upstream and downstream flanking regions for the in-frame deletion of *accF* (LuxR)*, accJ* (MarR)*, accI* (LysR)*,* and *accP* (MerR) genes were amplified from MA37 genomic DNA by a polymerase chain reaction (PCR) using primers with 15 bp overlaps at their 5’ ends (Tables S1 and S2). Each PCR mixture (25 µL) contained genomic DNA (50 ng), primers (1.0 µM), dNTP (0.1 mM, Novagen®), MgSO_4_ (0.1 mM), DMSO (4%), KOD buffer (10×), and KOD hot start DNA polymerase enzyme (0.5 µL). Reaction conditions consisted of an initial denaturation step at 95 °C for 5 min followed by 30 cycles at 95 °C for 30 s, annealing at 60 °C for 30 s, and a final extension step at 70 °C for 2 min. The PCR fragments were verified on agarose gel.

The amplified fragments were ligated into the linearized (HindIII, EcoRI) temperature-sensitive pKC1139 vector (Figure S1) via a one-pot In-Fusion® cloning following the protocol provided by the manufacturer (Clontech, TaKaRa, Shiga, Japan). The infusion reaction mixture (10 µL) consisted of the purified PCR fragment (50–100 ng), pKC1139 (50–100 ng), 5x infusion HD enzyme premix (2 µL), and water. The right construct was verified by PCR using a pair of primers (Table S2). 

### 2.4. Conjugations between Escherichia coli and Streptomyces sp. MA37

The confirmed deletion construct was introduced to *Escherichia coli* S17-1 by heat-shock (42 °C, 1 min), and the resulting strains were used as conjugal donors to MA37. The MA37 mycelia were used for conjugation as the strain produces no spores, and were prepared as described previously [24]. The mycelia (0.5 mL) was mixed with *E. coli* S17-1 (0.5 mL), followed by centrifugation (5 min). The supernatant was discarded, and the remaining mixture was plated onto SFM (soya flour (4 g), mannitol (4 g), agar (4 g), in 200 mL H_2_O) containing MgCl_2_ (10 mM). The plate was incubated at 28 °C. After 16–20 h, the plate was overlaid with water containing nalidixic acid (25 µg/mL, 1 mL) to inhibit the growth of *E. coli* and apramycin (50 µg/mL) for selection of successful exconjugants. Incubation was continued until the ex-conjugates appeared (28 °C, 3–5 days).

### 2.5. Screening for the Mutant Strain

The resulting ex-conjugants were colony-purified at 28 °C on ISP2 (glucose (4 g), yeast extract (4 g), malt extract (10 g), agar (20 g), in 1 L H_2_O) containing apramycin (50 µg/mL), and then re-streaked onto ISP2 with no antibiotic selection, and incubated at 37 °C for 1–3 generations to promote plasmid loss. Using the streak plate method, each single colony was inoculated separately onto ISP2 and ISP2 with apramycin (50 µg/mL). Strains sensitive to apramycin (indicating plasmid loss) were subsequently confirmed by PCR screening using internal primers. Glycerol stocks of the verified mutant strains (∆*accF,* ∆*accJ,* ∆*accI,* and ∆*accP*) were prepared and stored in an −80 °C freezer.

### 2.6. Metabolic Profile of Mutant and WT Strains

The seed cultures of ∆*accF,* ∆*accJ,* ∆*accI,* and ∆*accP* strains were prepared by inoculating 10 µL of the glycerol stocks in 10 mL YEME (yeast extract (3 g), tryptone (5 g), malt extract (3 g), glucose (10 g), sucrose (103 g), in 1 L H_2_O), and incubating for 3 days (28 °C, 180 rpm, Incu-shake FL16-2). The seed cultures were then used to prepare small-scale cultures (50 mL) in 250-mL Erlenmeyer flasks (Pyrex™ borosilicate glass narrow neck flask) containing ISP2 broth. 

The cultures were incubated (7 days, 28 °C, 180 rpm), after which Diaion® HP-20 (3 g/50 mL solution) was added. Incubation was continued for the next 18–24 h (28 °C, 180 rpm). The resin was filtered, extracted exhaustively with methanol, followed by concentration using a rotary evaporator (Buchi Rotavapor R200, Scotland, UK). Likewise, the MA37 WT strain was fermented and extracted as in the mutant strains. The mutants and WT extracts were subjected to mass spectrometric and HPLC-UV analyses monitored at λ450 nm, and the chemical profiles of each were compared. Several peaks were observed in the ∆*accJ* extract with the characteristic accramycin UV pattern (226, 250, 286, 355, and 420 nm) not detected in the WT or other mutant strains.

### 2.7. Fermentation, Extraction, Metabolite Screening

Two-liter fermentation culture of the ∆*accJ* mutant strain was carried out in ISP2 broth. Four 2.0-L baffled flasks (Corning™ polycarbonate), each containing 500 mL ISP2, were inoculated with seed culture (1:100), and plugged with foam stoppers (Fisherbrand™ polyurethane). Fermentation, incubation, and extraction of the ∆*accJ* cultures were carried out as described above. The methanol extracts were combined, evaporated to dryness under reduced pressure to yield crude extract (7 g), which was subjected to high-resolution electrospray ionization mass spectrometry (HRESIMS) analysis.

The crude extract was fractionated by vacuum liquid chromatography on silica gel 60 (Acros Organics™ ultra-pure 60A 40-63u) eluting with a gradient of *n*-hexane-ethyl acetate-MeOH to give 10 subfractions (F1-F10). HPLC-UV analysis was carried out in all the fractions to screen and target the accramycin scaffold using semi-prep reversed-phase HPLC (ACE C18-HL 10 µM 10 × 250 mm) equipped with a diode array detector (DAD) with spectral scanning between 200–550 nm. HPLC separation was carried out by the solvent gradient method over a period of 45 min (flow rate 1.5 mL/min, injection volume 10 µL, solvent A—95% H_2_O, 5% methanol, and 0.1% trifluoroacetic acid; solvent B—100% methanol). The compounds of interest with the characteristic accramycin UV maxima (226, 250, 286, 355, and 420 nm) were observed in fractions F3–F4.

### 2.8. Semi-Prep HPLC Isolation

Further purification of F3–F4 fractions was carried out using semi-prep reversed-phase HPLC as described above, eluting with a 45 min gradient of 40–100% methanol. The purification afforded 11 accramycins A–K **1**–**11** along with two other known compounds, naphthacemycin B_1_ **12** [26] and fasamycin C **13** [4].

Accramycin A **1**: Yield 131.4 mg; deep yellow powder; UV (PDA) λ_max_: 225, 245, 290, 355, 420 nm; IR (neat) ν_max_ (cm^−1^): 3350, 2946, 2834, 1681, 1607, 1284, 1202, 1026, 584; ^1^H, ^13^C NMR data, see Table S4; molecular formula: C_29_H_26_O_7_; HRESIMS (positive mode) *m/z* calculated for C_29_H_27_O_7_^+^ [M + H]^+^ = 487.1751; observed [M + H]^+^ = 487.1748; ∆ = −1.78 ppm.

Accramycin B **2**: Yield 6.12 mg; deep yellow powder; UV (PDA) λ_max_: 250, 290, 315, 335, 350, 425 nm; IR (neat) ν_max_ (cm^−1^): 3350, 2946, 2834, 1681, 1607, 1284, 1202, 1026, 584; molecular formula: C_30_H_28_O_7_; ^1^H, ^13^C NMR data, see Table S4; HRESIMS (positive mode) *m/z* calculated for C_30_H_29_O_7_^+^ [M + H]^+^ = 501.1912; observed [M + H]^+^ = 501.1898; ∆ = −1.98 ppm.

Accramycin C **3**: Yield 6.04 mg; deep yellow powder; UV (PDA) λ_max_: 250, 280, 300, 355, 430 nm; IR (neat) ν_max_ (cm^−1^): 3362, 2922, 2848, 1679, 1612, 1443, 1203, 1149, 726; molecular formula: C_28_H_23_ClO_7_; ^1^H, ^13^C NMR data, see Table S4; HRESIMS (positive mode) *m/z* calculated for C_28_H_24_ClO_7_^+^ [M + H]^+^ = 507.1205; observed [M + H]^+^ = 507.1207; ∆ = 0.37 ppm.

Accramycin D **4**: Yield 6.42 mg; deep yellow powder; UV (PDA) λ_max_: 250, 290, 315, 335, 350, 425 nm; IR (neat) ν_max_ (cm^−1^) 3337, 2947, 2834, 1681, 1450, 1025, 634; ^1^H, ^13^C NMR data, see Table S4; molecular formula: C_30_H_27_ClO_7_; HRESIMS (positive mode) *m/z* calculated for C_30_H_28_ClO_7_^+^ [M + H]^+^ = 535.1518; observed [M + H]^+^ = 535.1511; ∆ = −1.86 ppm.

Accramycin E **5**: Yield 6.68 mg; deep yellow powder; UV (PDA) λ_max_: 250, 290, 315, 335, 350, 425 nm; IR (neat) ν_max_ (cm^−1^) 3337, 2947, 2834, 1681, 1450, 1025, 634; ^1^H, ^13^C NMR data, see Table S4; molecular formula: C_29_H_24_Cl_2_O_7_; HRESIMS (positive mode) *m/z* calculated for C_29_H_25_Cl_2_O_7_^+^ [M + H]^+^ = 555.0972; observed [M + H]^+^ = 555.0978; ∆ = 1.18 ppm.

Accramycin F **6**: Yield 7.04 mg; deep yellow powder; UV (PDA) λ_max_: 250, 290, 315, 335, 350, 425 nm; IR (neat) ν_max_ (cm^−1^) 3325, 2943, 2833, 1678, 1449, 1119, 1023, 635; ^1^H, ^13^C NMR data, see Table S4; molecular formula: C_29_H_24_Cl_2_O_7_; HRESIMS (positive mode) *m/z* calculated for C_29_H_25_Cl_2_O_7_^+^ [M + H]^+^ = 555.0972; observed [M + H]^+^ = 555.0975; ∆ = 0.63 ppm.

Accramycin G **7**: Yield 35.24 mg; deep yellow powder; UV (PDA) λ_max_: 225, 250, 295, 320, 340, 350, 425 nm; IR (neat) ν_max_ (cm^−1^) 3399, 2917, 2851, 1688, 1606, 1423, 1322, 1205; ^1^H, ^13^C NMR data, see Table S4; molecular formula: C_30_H_26_Cl_2_O_7_; HRESIMS (positive mode) *m/z* calculated for C_30_H_27_Cl_2_O_7_^+^ [M + H]^+^ = 569.1128; observed [M + H]^+^ = 569.1127; ∆ = −0.29 ppm.

Accramycin H **8**: Yield 6.24 mg; deep yellow powder; UV (PDA) λ_max_: 250, 290, 310, 355, 425 nm; IR (neat) ν_max_ (cm^−1^) 3337, 2946, 1678, 1448, 1204, 1021, 644; ^1^H, ^13^C NMR data, see Table S4; molecular formula: C_28_H_21_Cl_3_O_7_; HRESIMS (positive mode) *m/z* calculated for C_28_H_22_Cl_3_O_7_^+^ [M + H]^+^ = 575.0426; observed [M + H]^+^ = 575.0420; ∆ = −0.99 ppm.

Accramycin I **9**: Yield 13.58 mg; deep yellow powder; UV (PDA) λ_max_: 250, 290, 315, 340, 350, 425 nm; IR (neat) ν_max_ (cm^−1^) 3399, 2921, 2849, 1680, 1442, 1196, 1139; ^1^H, ^13^C NMR data, see Table S4; molecular formula: C_29_H_23_Cl_3_O_7_; HRESIMS (positive mode) *m/z* calculated for C_29_H_24_Cl_3_O_7_^+^ [M + H]^+^ = 589.0582; observed [M + H]^+^ = 589.0574; ∆ = −1.32 ppm.

Accramycin J **10**: Yield 15.04 mg; deep yellow powder; UV (PDA) λ_max_: 250, 290, 315, 340, 350, 425 nm; IR (neat) ν_max_ (cm^−1^) 3427, 1688, 1601, 1439, 1328, 1204, 1139; ^1^H, ^13^C NMR data, see Table S4; molecular formula: C_28_H_20_Cl_4_O_7_; HRESIMS (positive mode) *m/z* calculated for C_28_H_21_Cl_4_O_7_^+^ [M + H]^+^ = 609.0036; observed [M + H]^+^ = 609.0040; ∆ = 0.72 ppm.

Accramycin K **11**: Yield 6.84 mg; deep yellow powder; UV (PDA) λ_max_: 225, 250, 295, 320, 340, 350, 425 nm; IR (neat) ν_max_ (cm^−1^) 3427, 1688, 1601, 1439, 1328, 1204, 1139; ^1^H, ^13^C NMR data, see Table S4; molecular formula: C_29_H_22_Cl_4_O_7_; HRESIMS (positive mode) *m/z* calculated for C_29_H_23_Cl_4_O_7_^+^ [M + H]^+^ = 623.0192; observed [M + H]^+^ = 623.0184; ∆ = 0.41 ppm.

Naphthacemycin B_1_ **12**: Yield 86.38 mg; reddish powder; UV (PDA) λ_max_: 245, 290, 355, 420 nm; IR (neat) ν_max_ (cm^−1^) 3337, 2946, 1678, 1448, 1204, 1021, 644; ^1^H, ^13^C NMR data, see Table S4; molecular formula: C_27_H_22_O_7_; HRESIMS (positive mode) *m/z* calculated for C_27_H_23_O_7_^+^ [M + H]^+^ = 459.1438; observed [M + H]^+^ = 459.1435; ∆ = −1.86 ppm.

Fasamycin C **13**: Yield 51.68 mg; deep yellow powder; UV (PDA) λ_max_: 245, 290, 355, 420 nm; IR (neat) ν_max_ (cm^−1^) 3338, 2947, 2834, 1644, 1449, 1202, 1114, 1019, 617; ^1^H, ^13^C NMR data, see Table S4; molecular formula: C_28_H_24_O_7_; HRESIMS (positive mode) *m/z* calculated for C_28_H_25_O_7_^+^ [M + H]^+^ = 473.1595; observed [M + H]^+^ = 473.1598; ∆ = −1.08 ppm.

### 2.9. Minimum Inhibitory Concentration

Minimum inhibitory concentrations (MIC) of compounds **1**–**13** were determined against a range of Gram-positive bacteria, *Staphylococcus aureus* (ATCC 25923) and *Enterococcus faecalis* (ATCC 29212), and Gram-negative bacteria, *E. coli* (ATCC 25922), *Pseudomonas aeruginosa* (ATCC 27853), fungal pathogen, *Candida albicans* ATCC 10,231, as well as clinical strains of *Enterococcus faecium* K59–68 and *Enterococcus faecium* K60-39, and *Staphylococcus haemolyticus* 8-7A. The hospital *E. faecium* isolates (K59-68 and K60-39) were isolated from the bloodstream of patients at the University Hospital of North Norway [28], and belonged to the complex clonal 17 sub-cluster, which is highly resistant to ampicillin [28,29]. *S. haemolyticus* 8-7A was obtained from the same hospital; all clinical strains were provided courtesy of Prof. Kristin Hegstad. The activity of **1**–**13** was determined using a sequential 2-fold serial dilution of the compounds (50–0.10 µg/mL) in DMSO following the standard protocols recommended by the Clinical and Laboratory Standard Institute [30] and as previously described [25,31,32]. The MIC was defined as the lowest concentration of the compound that inhibited ≥ 95% bacterial growth after overnight incubation. Ampicillin (Sigma) was used as the antibiotic standard. 

### 2.10. GenBank Accession Number

The accramycin BGC was deposited in NCBI and was assigned the accession number MN477201.

## 3. Results and Discussion

Bioinformatics analysis suggested that the *acc* BGC encodes four putative pathway-specific regulators, including LuxR (AccF), MarR (AccJ), LysR (AccI), and MerR (AccP) transcriptional regulators (Table S1) [25]. To assess their roles in the production of accramycins, we carried out the in-frame deletion of these four genes, generating four MA37 variants, respectively. The resulting mutants were then cultivated in ISP2 (7 days) and subsequently extracted with methanol to yield four crude extracts. Among the four samples, only the extract from the ∆*acc*J variant displayed a significant metabolic profile in HPLC and high-resolution electrospray ionization mass spectrometry (HRESIMS) analyses compared with the WT (Figure S2). Of particular relevance was the presence of several new HPLC peaks with the characteristic UV pattern (226, 250, 286, 355, and 420 nm), which had identical UV absorption to the one of accramycin A we previously isolated [25]. This finding was also supported by the more intense yellow color of the ∆*accJ* culture compared to the WT, which pointed to the yellow naphthacene chromophore [4,25].

To further confirm the identities of the newly emerged metabolites in the ∆*accJ* variant, we set out a large-scale fermentation (2 L) for chemical workup and structural elucidation. The crude extract was first fractionated through vacuum liquid chromatography to generate ten fractions. HPLC analysis of these ten fractions confirmed the presence of the accramycin constituents in fractions 3 (F3) and 4 (F4). The HPLC-UV-targeted isolation approach afforded accramycin A **1** in a significantly improved titer (66 mg/L), a 330-fold increase compared to the one from the WT (0.2 mg/L) [25]. Additionally, several new accramycin analogues **2**–**11**, together with two known compounds naphthacemycin B_1_ **12** [26] and fasamycin C **13** [4], were isolated. Likewise, the titer of fasamycin C was enhanced in the mutant strain by 30-fold (26 mg/L).

### 3.1. Structure Elucidation

New accramycin analogues were obtained as yellowish to red powders. Inspection of the HRESIMS data and MS/MS fragmentation pattern indicated that compounds **1**–**2** are non-halogenated, **3**–**4** have one chlorine atom, **5**–**7** have two chlorine atoms, **8**–**9** are trichloro-substituted, and **10**–**11** are tetra-chlorinated. Thorough analyses of the UV, HRESIMS, and nuclear magnetic resonance (NMR) data of **1**, **12**, and **13** indicated that compounds **1**, **12**, and **13** are known metabolites, accramycin A [25], naphthacemycin B_1_ produced by *Streptomyces* sp. KB-3346-5 (Figures S3–S22) [1,26], and fasamycin C produced by *S. formicae* (Figures S21–S26) [4], respectively. The structures of accramycins B–K **2**–**11** (Figure 2, Figures S23–S76) were elucidated by comparison of the observed UV, molecular formulae, and NMR data with the reported data of **1**, **12**, and **13** (Tables S2 and S3).

Compound **2** had the molecular formula C_30_H_28_O_7_ deduced by HRESIMS (observed [M+H]^+^ = 501.1898, *calcd* [M+H]^+^ = 501.1912 for C_30_H_29_O_7_^+^, ∆ = −1.9 ppm), suggesting 17 degrees of unsaturation. Detailed analysis of the HRESIMS, 1D, and 2D NMR data of **2** revealed that it is similar to **1** [25] except for the presence of the methoxy moiety at C-2 in ring A, which was supported by the heteronuclear multiple bond correlation (HMBC) from H_3_-30 (δ_H_ 3.91) to C-2 (δ_C_ 166.5). Compound **2** was therefore identified as the new analogue, accramycin B.

The molecular formula C_28_H_23_ClO_7_ of compound **3** deduced by HRESIMS had 34 mass units more than **13,** which suggested that **3** was a mono-chlorinated analogue of **13**. This was supported by the isotope pattern observed for **3** (*m*/*z* 507.1205, *calcd* [M+H]^+^: 509.1166) in a 3:1 ratio. The lower-field shifting of carbon signal (δ_C-3_ 106.6) in ring A indicated that the electronegative chlorine atom was attached to C-3 in the structure. This was further supported by the HMBC correlation from H-1 (δ_H_ 6.96) to C-3. The cross peak from H_3_-28 (δ_H_ 3.80) to C-24 (δ_C_ 159.0) established the connectivity of the methoxy functional group at C-24 in **3**. 

HRESIMS analysis of compound **4** deduced the molecular formula C_30_H_27_ClO_7_ (observed [M+H]^+^ = 535.1511, *calcd* [M+H]^+^ = 535.1518 for C_28_H_24_ClO_7_^+^, ∆ = −1.4 ppm), and indicated that **4** had 34 Da difference (Cl) from **2.** The additional chlorine atom was assigned at C-3 based on the HMBC correlation from H-1 (δ_H_ 6.97) to C-3 (δ_C_ 106.3). 

Compounds E **5** and F **6** are isomers with the same molecular formula, C_29_H_24_Cl_2_O_7_ deduced by HR ESIMS. The 68 Da difference of **5**–**6** from **1** and the isotope fragmentation pattern (*m*/*z* 555.0978: 557.0944: 559.0925) in 9:6:1 ratio supported the presence of two chlorine atoms in the formula. Two chlorines were placed at C-3 and C-13 in **5**–**6,** based on the HMBC correlations from H-1 (δ_H_ 6.87) to C-3 (δ_C_ 106.8), H-11 (δ_H_ 7.05) to C-13 (δ_C_ 115.2), and H-1 (δ_H_ 7.00) to C-3 (δ_C_ 107.6), H-11 (δ_H_ 6.87) to C-13 (δ_C_ 112.9), respectively. They differed in the attachment of the two methoxy groups, where one was attached to C-24 in **5**–**6**, while the other was linked to C-12 in **5** and C-2 in **6**. This was confirmed by the correlations from H_3_-29 (δ_H_ 4.02) to C-12 (δ_C_ 155.8) and from H_3_-30 (δ_H_ 4.06) to C-2 (δ_C_ 161.5) in the HMBC spectra of **5** and **6**, respectively.

The ^1^H and ^13^C NMR spectra of compound **7** were similar to those of **6** except for ring D. The molecular formula of **7** C_30_H_26_Cl_2_O_7_ showed 14 mass units more than **6**, indicative of an additional methyl group in the structure which was assigned at C-12 based on the HMBC correlations from H_3_-29 (δ_H_ 4.02) to C-12 (δ_C_ 156.6).

Compound **8** is a tri-chlorinated isomer with the molecular formula, C_28_H_21_Cl_3_O_7_ deduced by HRESIMS. The ^1^H-NMR spectrum of **8** revealed only one methoxy group which was assigned at C-2 based on the HMBC cross-peaks between H_3_-30 (δ_H_ 4.05) and C-2 (δ_C_ 161.3). The three chlorines were designated based on the downfield carbon shifts observed in C-3 (δ_C_ 108.8), C-23 (δ_C_ 107.5), and C-25 (δ_C_ 113.2) in comparison with non-chlorinated **12**, the attachment of which was further supported by HMBC correlations from H-1 (δ_H_ 6.97) to C-3, H_3_-27 (δ_H_ 1.93) to C-23, and H-27 to C-25.

HRESIMS analysis established the molecular formula of compound **9**, C_29_H_23_Cl_3_O_7_ (*m*/*z* 589.0574 [M+H]^+^, 589.0582 *calcd.* for C_29_H_24_Cl_3_O_7_^+^), and had 14 Da difference from **8**. The ^1^H-NMR spectrum of **9** in comparison with **8** confirmed the presence of an additional methoxy substituent, and its location was established at C-12 of ring D based on the nuclear Overhauser enhancement spectroscopy (NOESY) correlation between H_3_-29 (δ_H_ 4.02) and H-11 (δ_H_ 7.06). The three chlorines identified in the formula were assigned at C-3, C-13, and C-25 based on the HMBC correlations from H-1 (δ_H_ 7.00) to C-3 (δ_C_ 108.1), H-11 (δ_H_ 7.06) to C-13 (δ_C_ 115.5), and H_3_-27 (δ_H_ 1.98) to C-25 (δ_C_ 112.1). 

The ^1^H and ^13^C NMR spectra of compound **10** were similar to **8** except for ring D. HRESIMS analysis of **10** showed 34 mass units more than **8**, indicating an additional chlorine substituent in the structure, which was assigned at C-13 based on the HMBC correlation from H-11 (δ_H_ 6.87) to C-13 (δ_C_ 113.8). 

The ^1^H-NMR spectrum of compound **11** showed an additional singlet signal in the methoxy region compared to **10**. The HMBC cross peak between H_3_-29 (δ_H_ 4.02) and C-12 (δ_C_ 156.2) designated the methoxy group at C-12 of ring D in the structure.

On the basis of the evidence in this study, compounds **2**–**11** were confirmed as new members of accramycin polyketides for which the names accramycin B–K are proposed, respectively.

### 3.2. Biological Activity

Compounds **1**–**13** exhibited good activity against the Gram-positive pathogens, *S. aureus* (ATCC 25923), *E. faecalis* (ATCC 29212), and clinical isolates *E. faecium* K59-68, *E. faecium* K60-39, and *S. haemolyticus* 8-7A with MIC values of 1.5–12.5 µg/mL (Table 1). The presence of multiple chlorines at rings A, D, or E did not enhance the activity as observed in accramycins C–K consistent with previous findings [7]. The *O*-methyl-bearing accramycin B exhibited slightly higher MIC against all the tested pathogens. Conversely, the presence of free hydroxyl groups at C-12 and C-24 favored antibacterial activity as observed in accramycin J. Compound **10** in comparison with naphthacemycin B_1_ supported that the *O*-methyl at C-2 is a preferred structural feature for the activity. Furthermore, accramycin J (MIC = 6.3 µg/mL) exhibited 2-fold inhibitory activity over ampicillin (MIC = 12.5 µg/mL) against the clinical isolate, *S. haemolyticus*. Notably, compounds **1**–**13** had superior activity (MIC = 3.1–6.3 µg/mL) against *E. faecium* K60-39 than ampicillin (MIC = 25 µg/mL). Thus, the accramycins represent potential therapeutic lead molecules for the development of potent drugs against ampicillin-resistant *Enterococcus* clinical strains. None of the compounds **1**–**13** displayed activity against the Gram-negative pathogens, *E. coli* (ATCC 25922), and *P. aeruginosa* (ATCC 27853), and the fungal pathogen *C. albicans* (ATCC 10231) at the highest concentration tested (50 µg/mL). 

## 4. Conclusions

In conclusion, we confirmed that *accJ* is a repressor gene in accramycin biosynthesis. Inactivation of the *accJ* gene resulted in the production of thirteen fasamycin-type antibiotics in *Streptomyces* sp. MA37 ∆*accJ* mutant including accramycins A–K **1**–**11** along with two known compounds, naphthacemycin B_1_ **12** and fasamycin C **13**. The structures of compounds **1**–**13** were deduced by HRESIMS and 1D and 2D NMR. The fermentation titers of the isolated metabolites were significantly improved, particularly accramycin A with an estimated yield from 0.2 mg/L (WT strain) to 66 mg/L (∆*accJ* mutant strain). During the preparation of this manuscript, Devine et al. reported a similar observation in the producer of fasamycins and formicamycins, *S. formicae*, suggesting that inactivation of *forJ*, encoding the MarR regulator (homologue to AccJ), resulted in an improved titer of fasamycins and formicamycins [27]. Six new fasamycin analogues L–Q and two new formicamycins R–S were characterized in this variant [27]. Compounds **1**–**13** exhibited good activity against the Gram-positive pathogens, *S. aureus* and *E. faecalis*, and clinical isolates, *E. faecium* K59-68, *E. faecium* K60-39, and *S. haemolyticus* (MIC = 1.5–12.5 µg/mL). Remarkably, compounds **1**–**13** displayed better inhibitory activity over ampicillin against K60-39. Hence, the accramycin pharmacophore represents potential lead molecules for the development of potent antibiotics that target *Enterococcus* isolates.

## Figures and Tables

**Figure 2:**
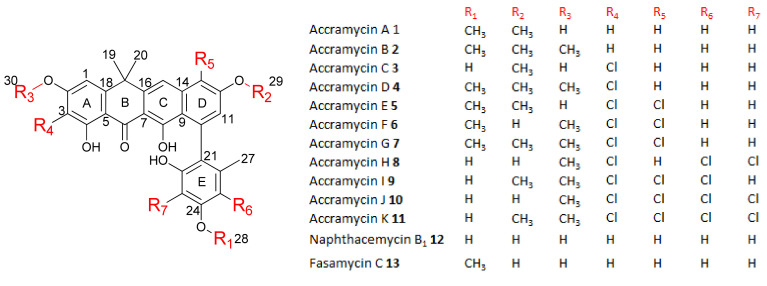
Accramycins A–K, naphthacemycin B_1_ and fasamycin C isolated from *Streptomyces* sp. MA37 (∆*accJ*) strain.

**Table 1:** Minimum inhibitory concentration (MIC) of compounds **1**–**13.**

Compound Name	MIC (µg/mL)				
	*S. aureus* ATCC 25923)	*E. faecalis* (ATCC 29212)	*E. faecium* K59–68 *	*E. faecium* K60–39 *	*S. haemolyticus* 8-7A *
Accramycin A **1**	12.5	3.1	6.3	6.3	12.5
Accramycin B **2**	12.5	6.3	12.5	6.3	12.5
Accramycin C **3**	6.3	3.1	6.3	6.3	12.5
Accramycin D **4**	12.5	1.5	6.3	6.3	12.5
Accramycin E **5**	12.5	1.5	6.3	6.3	12.5
Accramycin F **6**	12.5	1.5	6.3	6.3	12.5
Accramycin G **7**	12.5	1.5	6.3	6.3	12.5
Accramycin H **8**	6.3	1.5	6.3	6.3	12.5
Accramycin I **9**	6.3	1.5	3.1	6.3	12.5
Accramycin J **10**	3.1	1.5	1.5	3.1	6.3
Accramycin K **11**	6.3	3.1	6.3	6.3	12.5
Naphthacemycin B_1_ **12**	3.1	6.3	6.3	3.1	12.5
Fasamycin C **13**	6.3	6.3	6.3	3.1	12.5
Ampicillin	0.5	1.0	1.5	25	12.5

* Clinical isolate.

## Supplementary Materials

The following are available online at https://www.mdpi.com/2218-273X/10/10/1464/s1.
