# Supplementary material for: Discovery of New Antibacterial Accramycins from a Genetic Variant of the Soil Bacterium, Streptomyces sp. MA37
Source: Biomolecules. 2020 Oct 20;10(10):1464. doi: 10.3390/biom10101464 (PMC7590149; doi:10.3390/biom10101464)
Supplement: Supplementary file 1 [file biomolecules-10-01464-s001.pdf]

SUPPLEMENTARY INFORMATION

Discovery of new antibacterial accramycins from a genetic variant of the soil bacterium, *Streptomyces* sp. MA37

Fleurdeliz Maglangit[1,2]*, Yuting Zhang[1], Kwaku Kyeremeh[3], Hai Deng[1]*

List of Tables

Table S1. Deduced functions of ORFs in acc biosynthetic gene cluster
Table S2. Primers used in the Study
Table S3. Physico-chemical Properties of Accramycins A-K **1-11**
Table S4. ^{1}H and ^{13}C of Accramycins A-K **1-11**, naphthacemycin B1 **12** and fasamycin C **13** (CD_3OD, 298K, 600MHz)

List of Figures

Figure S1. HPLC traces of *Streptomyces* sp. MA37 mutant strain compared to Wild Type
Figure S2. Key HMBC correlations (→) of accramycins A-K **1-11**, naphthacemycin B1 **12**, and fasamycin C **13** (CD_3OD, 298K, 600MHz)
Figure S3. HRESIMS of naphthacemycin B1 **12**
Figure S4. ^{1}H-NMR of naphthacemycin B1 **12** (CD_3OD, 298K, 600MHz)
Figure S5. ^{1}H-1H COSY of naphthacemycin B1 **12**(CD_3OD, 298K, 600MHz)
Figure S6. HSQC of naphthacemycin B1 **12** (CD_3OD, 298K, 600MHz)
Figure S7. HMBC of naphthacemycin B1 **12** (CD_3OD, 298K, 600MHz)
Figure S8. NOESY of naphthacemycin B1 **12** (CD_3OD, 298K, 600MHz)
Figure S9. HRESIMS of fasamycin C **13**
Figure S10. ^{1}H-NMR of fasamycin C **13** (CD_3OD, 298K, 600MHz)
Figure S11. ^{1}H-^{1}H COSY of fasamycin C **13** (CD_3OD, 298K, 600MHz)
Figure S12. HSQC of fasamycin C **13** (CD_3OD, 298K, 600MHz)
Figure S13. HMBC of fasamycin C **13** (CD_3OD, 298K, 600MHz)
Figure S14. NOESY of fasamycin C **13** (CD_3OD, 298K, 600MHz)
Figure S15. HRESIMS of Accramycin A **1**
Figure S16. ^{1}H-NMR of Accramycin A **1** (CD_3OD, 298K, 600MHz)
Figure S17. ^{1}H-^{1}H COSY of Accramycin A **1** (CD_3OD, 298K, 600MHz)
Figure S18. HSQC of Accramycin A **1** (CD_3OD, 298K, 600MHz)
Figure S19. HMBC of Accramycin A **1** (CD_3OD, 298K, 600MHz)
Figure S20. NOESY of Accramycin A **1** (CD_3OD, 298K, 600MHz)
Figure S21. HRESIMS of Accramycin B **2**
Figure S22. ^{1}H-NMR of Accramycin B **2** (CD_3OD, 298K, 600MHz)
Figure S23. ^{1}H-^{1}H COSY of Accramycin B **2** (CD_3OD, 298K, 600MHz)
Figure S24. HSQC of Accramycin B **2** (CD_3OD, 298K, 600MHz)
Figure S25. HMBC of Accramycin B **2** (CD_3OD, 298K, 600MHz)
Figure S26. **A.** HRESIMS and **B**. Isotope Pattern of Accramycin C **3**
Figure S27. ^{1}H-NMR of Accramycin C **3** (CD_3OD, 298K, 600MHz)
Figure S28. ^{1}H-^{1}H COSY of Accramycin C **3** (CD_3OD, 298K, 600MHz)
Figure S29. HSQC of Accramycin C **3** (CD_3OD, 298K, 600MHz)
Figure S30. NOESY of Accramycin C **3** (CD_3OD, 298K, 600MHz)
Figure S31. **A**. HRESIMS and **B**. Isotope Pattern of Accramycin D **4**
Figure S32. ^{1}H-NMR of Accramycin D **4** (CD_3OD, 298K, 600MHz)
Figure S33. ^{1}H-^{1}H COSY of Accramycin D **4** (CD_3OD, 298K, 600MHz)
Figure S34. HSQC of Accramycin D **4** (CD_3OD, 298K, 600MHz)
Figure S35. HMBC of Accramycin D **4** (CD_3OD, 298K, 600MHz)

Figure S36. **A.** HRESIMS and **B.** Isotope Pattern of Accramycin E **5**
Figure S37. ^{1}H-NMR of Accramycin E **5** (CD_3OD, 298K, 600MHz)
Figure S38. ^{1}H-^{1}H COSY of Accramycin E **5** (CD_3OD, 298K, 600MHz)
Figure S39. HSQC of Accramycin E **5** (CD_3OD, 298K, 600MHz)
Figure S40. HMBC of Accramycin E **5** (CD_3OD, 298K, 600MHz)
Figure S41. **A.** HRESIMS and **B.** Isotope Pattern of Accramycin F **6**
Figure S42. ^{1}H-NMR of Accramycin F **6** (CD_3OD, 298K, 600MHz)
Figure S43. ^{1}H-^{1}H COSY of Accramycin F **6** (CD_3OD, 298K, 600MHz)
Figure S44. HSQC of Accramycin F **6** (CD_3OD, 298K, 600MHz)
Figure S45. HMBC of Accramycin F **6** (CD_3OD, 298K, 600MHz)
Figure S46. NOESY of Accramycin F **6** (CD_3OD, 298K, 600MHz)
Figure S47. **A.** HRESIMS and **B.** Isotope Pattern of Accramycin G **7**
Figure S48. ^{1}H-NMR of Accramycin G **7** (CD_3OD, 298K, 600MHz)
Figure S49. ^{1}H-^{1}H COSY of Accramycin G **7** (CD_3OD, 298K, 600MHz)
Figure S50. HSQC of Accramycin G **7** (CD_3OD, 298K, 600MHz)
Figure S51. HMBC of Accramycin G **7** (CD_3OD, 298K, 600MHz)
Figure S52. NOESY of Accramycin G **7** (CD_3OD, 298K, 600MHz)
Figure S53. **A.** HRESIMS and **B.** Isotope Pattern of Accramycin H **8**
Figure S54. ^{1}H-NMR of Accramycin H **8** (CD_3OD, 298K, 600MHz)
Figure S55. ^{1}H-^{1}H COSY of Accramycin H **8** (CD_3OD, 298K, 600MHz)
Figure S56. HSQC of Accramycin H **8** (CD_3OD, 298K, 600MHz)
Figure S57. HMBC of Accramycin H **8** (CD_3OD, 298K, 600MHz)
Figure S58. **A.** HRESIMS and **B.** Isotope Pattern of Accramycin I **9**
Figure S59. ^{1}H-NMR of Accramycin I **9** (CD_3OD, 298K, 600MHz)
Figure S60. ^{1}H-^{1}H COSY of Accramycin I **9** (CD_3OD, 298K, 600MHz)
Figure S61. HSQC of Accramycin I **9** (CD_3OD, 298K, 600MHz)
Figure S62. HMBC of Accramycin I **9** (CD_3OD, 298K, 600MHz)
Figure S63. NOESY of Accramycin I **9** (CD_3OD, 298K, 600MHz)
Figure S64. LCMS isotope pattern of Accramycin J **10**
Figure S65. ^{1}H-NMR of Accramycin J **10** (CD_3OD, 298K, 600MHz)
Figure S66. ^{1}H-^{1}H COSY of Accramycin J **10** (CD_3OD, 298K, 600MHz)
Figure S67. HSQC of Accramycin J **10** (CD_3OD, 298K, 600MHz)
Figure S68. HMBC of Accramycin J **10** (CD_3OD, 298K, 600MHz)
Figure S69. NOESY of Accramycin J **10** (CD_3OD, 298K, 600MHz)
Figure S70. LCMS Isotope Pattern of Accramycin K **11**
Figure S71. ^{1}H-NMR of Accramycin K **11** (CD_3OD, 298K, 600MHz)
Figure S72. ^{1}H-^{1}H COSY of Accramycin K **11** (CD_3OD, 298K, 600MHz)
Figure S73. HSQC of Accramycin K **11** (CD_3OD, 298K, 600MHz)
Figure S74. HMBC of Accramycin K **11** (CD_3OD, 298K, 600MHz)
Figure S75. NOESY of Accramycin K **11** (CD_3OD, 298K, 600MHz)

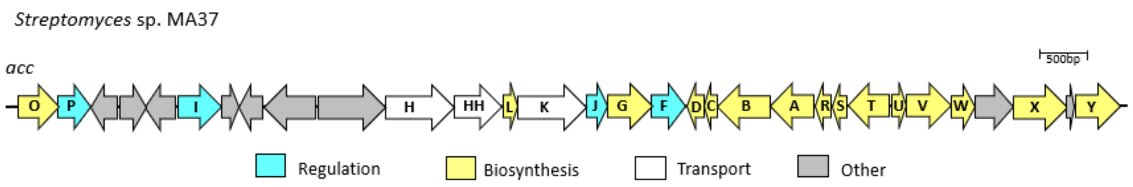

Table S1. Deduced functions of ORFs in *acc* biosynthetic gene cluster

acc	Residue	Deduced Function
O	305	NAD(P) dependent oxidoreductase
P	275	MerR family transcriptional regulator
I	303	LysR family transcriptional regulator
H	491	ABC transporter / ATP binding protein
HH	319	ABC transporter / ATP binding protein
L	119	Type II PKS cyclase
K	509	Na^+/H^+ exchanger
J	145	MarR family transcriptional regulator
G	359	Sensor histidine kinase / hypothetical protein
F	227	LuxR family response regulator
D	152	polyketide cyclase / dehydrase
C	97	Acyl carrier protein
B	415	Beta-ketoacyl synthase
A	426	Beta-ketoacyl synthase
R	131	AraC family transcriptional regulator
S	113	antibiotic biosynthesis monooxygenase
T	350	O-methyl transferase regulator / methyl transferase
U	113	antibiotic biosynthesis monooxygenase
V	430	Halogenase / FAD dependent oxidoreductase
W	348	O-methyl transferase regulator / methyl transferase
X	777	glycoside hydrolase family 92 protein
Y	517	pyruvate oxidase decarboxylase

Table S2. Primers used in the Study

Primer ID	Primer Sequence	Purpose
MarR-FRA	CATGACCTCTAGACTCAAGAAGGCCTCCG C GAACTGA	Right Arm Forward: Construction of knockout vector
MarR-RRA	ACATGATTACGAATTCGGATGCGCTGGGT G CAGGACTT	Right Arm Reverse: Construction of knockout vector
MarR-FLA	GGCCAGTGCCAAGCTTTCCCGCCATGCAC A CCACACTGAT	Left Arm Forward: Construction of knockout vector
MarR-RLA	TCTTGAGTCTAGAGGTCATGGTCGGTCAC C TCTGCCCT	Left Arm Reverse: Construction of knockout vector
LuxR-FRA	AATTCGTAATCATGTCATAGCTGTTTCCTG TG	Right Arm Forward: Construction of knockout vector
LuxR-RRA	ACATGATTACGAATTTCATCCGCGACCGG ATCGA	Right Arm Reverse: Construction of knockout vector
LuxR-FLA	GGCCAGTGCCAAGCTGTGAGCGGGGGC TGCGC	Left Arm Forward: Construction of knockout vector
LuxR-RLA	TGGCACTGGCCGTCGTTTTACAACGTCGTG AC TGGG	Left Arm Reverse: Construction of knockout vector
LysR-FRA	AATTCGTAATCATGTCATAGCTGTTTCCTG TG	Right Arm Forward: Construction of knockout vector
LysR-RRA	ACATGATTACGAATTTCAGGTGTCGGC GCCG	Right Arm Reverse: Construction of knockout vector
LysR-FLA	GGCCAGTGCCAAGCTATGGAGCTCCGGCA GCTGCA	Left Arm Forward: Construction of knockout vector
LysR-RLA	TGGCACTGGCCGTCGTTTTACAACGTC GTGACTG	Left Arm Reverse: Construction of knockout vector
MerR-FRA	AATTCGTAATCATGTCATAGCTGTTTCCTG TG	Right Arm Forward: Construction of knockout vector
MerR-RRA	ACATGATTACGAATTTCACCCGGTGGTGAT CGGCTCCTG	Right Arm Reverse: Construction of knockout vector

MerR-FLA	CGACGGCCAGTGCCAATGTTCAGTATCGG AGACTTCGC	Left Arm Forward: Construction of knockout vector
MerR-RLA	TGGCACTGGCCGTCGTTTTACAACGTCGT GACTGGG	Left Arm Reverse: Construction of knockout vector
Mu-F	CGCACCCTTCTGTCGGACCACCACTGA	Mutant verification
Mu-R	GTTCTGCTTGGCGACGCTGACCGAGTA	Mutant verification

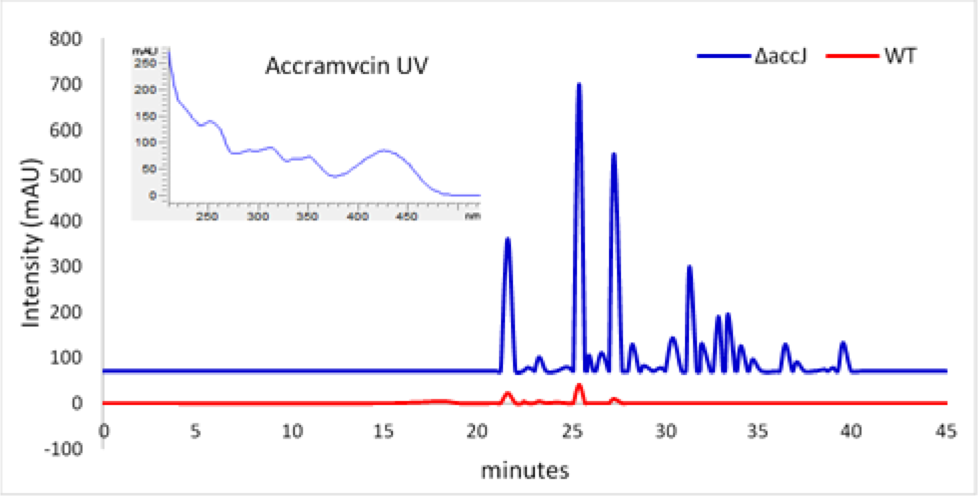

Figure S1. HPLC traces monitored at λ450nm of Streptomyces sp. MA37 mutant strain (blue) compared to Wild Type (red) (5μL injection, 5mg/mL) with the characteristic accramycin UV spectrum

Table S3. Physico-chemical Properties of Accramycins A-K **1-11**, naphthacemycin B1 **12**, and fasamycin C **13** from *Streptomyces* sp. MA37

	Accramycin A **1**	Accramycin B **2**	Accramycin C **3**	Accramycin D **4**
Appearance	deep yellow powder	deep yellow powder	deep yellow powder	deep yellow powder
Molecular formula	$C_{29}H_{26}O_7$	$C_{30}H_{28}O_7$	$C_{28}H_{23}ClO_7$	$C_{30}H_{27}ClO_7$
HR ESIMS (*obs*)	487.1748 $[M+H]^+$	501.1898 $[M+H]^+$	507.1207 $[M+H]^+$	535.1511 $[M+H]^+$
m/z (*calc*)	487.1751 (for $C_{29}H_{27}O_7^+$)	501.1912 (for $C_{30}H_{29}O_7^+$)	507.1205 (for $C_{28}H_{24}ClO_7^+$)	535.1518 (for $C_{30}H_{28}ClO_7^+$)
Δ ppm	-1.78	-1.98	0.37	-1.39
IR ν_{max} (cm^{-1})	3350, 2946, 2834, 1681, 1607, 1284, 1202, 1026, 584	3350, 2946, 2834, 1681, 1607, 1284, 1202, 1026, 584	3362, 2922, 2848, 1679, 1612, 1443, 1203, 1149, 726	3337, 2947, 2834, 1681, 1450, 1025, 634
UV (PDA) λ_{max}	225, 245, 290, 355, 420	250, 290, 315, 335, 350, 425	250, 280, 300, 355, 430	250, 290, 315, 335, 350, 425

	Accramycin E **5**	Accramycin F **6**	Accramycin G **7**	Accramycin H **8**
Appearance	deep yellow powder	deep yellow powder	deep yellow powder	deep yellow powder
Molecular formula	$C_{29}H_{24}Cl_2O_7$	$C_{29}H_{24}Cl_2O_7$	$C_{30}H_{26}Cl_2O_7$	$C_{28}H_{21}Cl_3O_7$
HR ESIMS (*obs*)	555.0978 $[M+H]^+$	555.0975 $[M+H]^+$	569.1127 $[M+H]^+$	575.0420 $[M+H]^+$
m/z (*calc*)	555.0972 (for $C_{29}H_{25}Cl_2O_7^+$)	555.0972 (for $C_{29}H_{25}Cl_2O_7^+$)	569.1128 (for $C_{30}H_{27}Cl_2O_7^+$)	575.0426 (for $C_{28}H_{22}Cl_3O_7^+$)
Δ ppm	1.18	0.63	-0.29	-0.99
IR ν_{max} (cm^{-1})	3337, 2947, 2834, 1681, 1450, 1025, 634	3325, 2943, 2833, 1678, 1449, 1119, 1023, 635	3399, 2917, 2851, 1688, 1606, 1423, 1322, 1205	3386, 1682, 1439, 1327, 1197, 1136, 844, 802, 725
UV (PDA) λ_{max}	250, 290, 315, 335, 350, 425	250, 290, 315, 335, 350, 425	250, 295, 320, 340, 350, 425	250, 290, 310, 355, 425

Table S3. Physico-chemical Properties of Accramycins A-K **1-11**, naphthacemycin B1 **12**, and fasamycin C **13** from *Streptomyces* sp. MA37

	Accramycin I **9**	Accramycin J **10**	Accramycin K **11**
Appearance	deep yellow powder	deep yellow powder	deep yellow powder
Molecular formula	$C_{29}H_{23}Cl_3O_7$	$C_{28}H_{20}Cl_4O_7$	$C_{29}H_{22}Cl_4O_7$
HR ESIMS (*obs*)	589.0574 $[M+H]^+$	609.0040 $[M+H]^+$	623.0184 $[M+H]^+$
m/z (*calc*)	589.0582 (for $C_{29}H_{24}Cl_3O_7^+$)	609.0036 (for $C_{28}H_{21}Cl_4O_7^+$)	623.0192 (for $C_{29}H_{23}Cl_4O_7^+$)
Δ ppm	-1.32	0.72	0.41
IR ν_{max} (cm^{-1})	3399, 2921, 2849, 1680, 1442, 1196, 1139	3427, 1688, 1601, 1439, 1328, 1204, 1139	3427, 1688, 1601, 1439, 1328, 1204, 1139
UV (PDA) λ_{max}	250, 290, 315, 340, 350, 425	250, 290, 315, 340, 350, 425	250, 295, 320, 340, 350, 425

	Naphthacemycin B1 **12**	Fasamycin C **13**
Appearance	reddish powder	deep yellow powder
Molecular formula	$C_{27}H_{22}O_7$	$C_{28}H_{24}O_7$
HR ESIMS (*obs*)	459.1435 $[M+H]^+$	473.1598 $[M+H]^+$
m/z (*calc*)	459.1438 (for $C_{27}H_{23}O_7^+$)	473.1595 (for $C_{28}H_{25}O_7^+$)
Δ ppm	-1.86	-1.08
IR ν_{max} (cm^{-1})	3337, 2946, 1678, 1448, 1204, 1021, 644	3338, 2947, 2834, 1644, 1449, 1202, 1114, 1019, 617
UV (PDA) λ_{max}	245, 290, 355, 420	245, 290, 355, 420

Table S4. ^{1}H and ^{13}C of Accramycins A-K **1-11**, naphthacemycin B1 **12** and fasamycin C **13** (CD_3OD, 298K, 600MHz)

	Accramycin A **1**		Accramycin B **2**		Accramycin C **3**		Accramycin D **4**		Accramycin E **5**	
**no.**	^{13}C	^{1}H, mult. (J,Hz)	^{13}C	^{1}H, mult. (J,Hz)	^{13}C	^{1}H, mult. (J,Hz)	^{13}C	^{1}H, mult. (J,Hz)	^{13}C	^{1}H, mult. (J,Hz)
1	105.9, CH	6.67, d (2.4)	106.1, CH	6.66, d (1.4)	105.4, CH	6.84, s	101.0, CH	6.97, s	105.7, CH	6.87, s
2	165.7, C	-	166.5, C	-		-	161.2, C	-	160.7, C	-
3	100.9, CH	6.22, d (2.4)	98.7, CH	6.42, d (2.0)		-	106.3, C	-	106.8, C	-
4	165.7, C	-	165.8, C	-		-	161.3, C	-	160.7, C	-
5	107.5, C	-	108.0, C	-		-	108.4, C	-	108.4, C	-
6	190.4, C	-	190.8, C	-		-	190.4, C	-	190.4, C	-
7	107.4, C	-	106.2, C	-		-	106.3, C	-	106.6, C	-
8	165.3, C	-	165.0, C	-		-	165.7, C	-	165.7, C	-
9	117.4, C	-	117.6, C	-		-	116.9, C	-	117.4, C	-
10	141.0, C	-	141.0, C	-		-	141.9, C	-	137.3, C	-
11	121.7, CH	6.75, d (2.4)	121.4, CH	6.77, d (2.1)	121.1, CH	6.73, d (2.1)	121.4, CH	6.77, d (2.1)	115.9, CH	7.05, d (2.1)
12	161.1, C	-	160.9, C			-	161.2, C	-	155.8, C	-
13	105.8, CH	7.21, d (2.4)	105.8, CH	7.25, d (2.0)	109.0, CH	7.08, d (2.1)	105.9, CH	7.25, d (2.1)	115.2, C	-
14	141.8, C	-	141.3, C			-	141.9, C	-	138.4, C	-
15	115.8, CH	7.51, s	115.5, CH	7.56, s	115.0, CH	7.39, s	115.8, CH	7.57, s	111.2, CH	7.95, s
16	145.3, C	-	145.3, C	-	144.8, C	-	145.3, C	-	146.4, C	-
17	39.3, C	-	38.6, C	-	38.7, C	-	38.4, C	-	38.4, C	-
18	154.6, C	-	154.0, C	-	151.9, C	-	152.4, C	-	151.7, C	-
19	34.4, CH_3	1.70, s	33.3, CH_3	1.77, s	33.1, CH_3	1.72, s	33.4, CH_3	1.80, s	33.4, CH_3	1.77, s
20	34.7, CH_3	1.69, s	33.4, CH_3	1.75, s	33.1, CH_3	1.71, s	33.4, CH_3	1.79, s	33.4, CH_3	1.75, s
21	123.9, C	-	124.4 C	-	124.5, C	-	123.9, C	-	123.9, C	-
22	154.2, C	-	154.5, C	-		-	154.3, C	-	154.3, C	-
23	98.3, CH	6.33, d (2.4)	98.3, CH	6.35, s	98.3, CH	6.33, d (2.1)	98.4, CH	6.34, d (2.1)	98.4, CH	6.35, d (2.1)
24	159.1, C	-	159.2,C	-	158.9, C	-	159.5, C	-	159.3, C	-
25	105.9, CH	6.37, d (2.4)	106.0, CH	6.40, s	106.5, CH	6.38, d (2.1)	105.9, CH	6.38, d (2.1)	105.9, CH	6.40, d (2.1)
26	137.0, C	-	136.9, C		136.8, C	-	136.8, C	-	136.8, C	-
27	20.6, CH_3	1.91, s	19.5, CH_3	1.93, s	19.5, CH_3	1.92, s	19.4, CH_3	1.91, s	19.4, CH_3	1.93, s
28	55.3, CH_3	3.80, s	54.3, CH_3	3.83, s	54.3, CH_3	3.80, s	54.2, CH_3	3.81, s	54.2, CH_3	3.81, s
29	55.6, CH_3	3.95, s	54.7, CH_3	3.99, s	-	-	54.7, CH_3	3.98, s	55.7, CH_3	4.02, s
30	-	-	54.9, CH_3	3.91, s	-	-	55.8, CH_3	4.05, s	-	-

Table S4. ^{1}H and ^{13}C of Accramycins A-K **1-11**, naphthacemycin B1 **12** and fasamycin C **13** (CD_3OD, 298K, 600MHz)

	Accramycin F **6**		Accramycin G **7**		Accramycin H **8**		Accramycin I **9**		Accramycin J **10**	
**no.**	^{13}C	^{1}H, mult. (J,Hz)	^{13}C	^{1}H, mult. (J,Hz)	^{13}C	^{1}H, mult. (J,Hz)	^{13}C	^{1}H, mult. (J,Hz)	^{13}C	^{1}H, mult. (J,Hz)
1	101.5, CH	7.00, s	101.3, CH	7.00, s	101.1, CH	6.97, s	101.5, CH	7.00, s	101.5, CH	7.00, s
2	161.5, C	-	161.9, C	-	161.3, C	-	161.6, C	-	161.9, C	-
3	107.6, C	-	108.1, C	-	108.8, C	-	108.1, C	-	108.0, C	-
4	161.7, C	-	161.7, C	-	160.8, C	-	161.8, C	-	162.0, C	-
5	108.1, C	-	108.1, C	-	108.8, C	-	108.1, C	-	108.0, C	-
6	190.6, C	-	190.6, C	-	190.0, C	-	190.5, C	-	190.7, C	-
7	106.8, C	-	106.7, C	-	106.6, C	-	107.0, C	-	107.2, C	-
8	165.7, C	-	166.0, C	-	165.5, C	-	164.3, C	-	165.0, C	-
9	117.7, C	-	118.0, C	-	116.8, C	-	118.0, C	-	117.7, C	-
10	137.3, C	-	138.5, C	-	141.3, C	-	138.4, C	-	137.1, C	-
11	120.9, CH	6.87, d (2.1)	116.2, CH	7.07, s	121.3, CH	6.74, s	116.3, CH	7.06, s	120.9, CH	6.87, s
12	154.9, C	-	156.6, C	-	163.5, C	-	155.9, C	-	155.0, C	-
13	112.9, C	-	115.0, C	-	109.4, CH	7.14, d (2.1)	115.5, C	-	113.8, C	-
14	138.4, C	-	146.5, C	-	142.5, C	-	138.4, C	-	138.4, C	-
15	111.2, CH	7.93, s	111.3, CH	7.98, s	115.2, CH	7.45, s	111.3, CH	7.99, s	111.7, CH	7.95, s
16	146.7, C	-	146.5, C	-	148.5, C	-	146.9, C	-	146.8, C	-
17	39.4, C	-	39.1, C	-	39.4, C	-	39.5, C	-	39.6, C	-
18	152.5, C	-	152.5, C	-	153.2, C	-	152.5, C	-	152.5, C	-
19	33.4, CH_3	1.83, s	33.4, CH_3	1.83, s	33.2, CH_3	1.79, s	33.5, CH_3	1.83, s	33.5, CH_3	1.83, s
20	33.4, CH_3	1.82, s	33.4, CH_3	1.82, s	33.2, CH_3	1.78, s	33.5, CH_3	1.82, s	33.5, CH_3	1.82, s
21	124.2, C	-	123.7, C	-	125.5, C	-	124.5, C	-	124.9, C	-
22	159.5, C	-	159.4, C	-	169.0, C	-	152.5, C	-	152.5, C	-
23	98.1, CH	6.32, d (2.3)	98.3, CH	6.34, d (2.3)	107.5, C	-	100.7, CH	6.44, s	107.5, CH	-
24	159.3, C	-	159.4, C	-	169.0, C	-	152.5, C	-	148.4, C	-
25	106.3, CH	6.38, d (2.3)	105.9, CH	6.40, d (2.3)	113.2, C	-	112.1, C	-	113.2, C	-
26	137.2, C	-	137.0, C	-	133.1, C	-	134.7, C	-	132.5, C	-
27	19.4, CH_3	1.93, s	19.4, CH_3	1.93, s	16.9, CH_3	1.93, s	17.1, CH_3	1.98, s	16.9, CH_3	1.98, s
28	54.4, CH_3	3.80, s	54.2, CH_3	3.81, s	-	-	-	-	-	-
29	-	-	55.7, CH_3	4.02, s	-	-	56.0, CH_3	4.02, s	-	-
30	55.8, CH_3	4.06, s	55.7, CH_3	4.06, s	55.7, CH_3	4.05, s	55.7, CH_3	4.06, s	56.0, CH_3	4.06, s

Table S4. ^{1}H and ^{13}C of Accramycins A-K **1-11**, naphthacemycin B1 **12** and fasamycin C **13** (CD_3OD, 298K, 600MHz)

	Accramycin K **11**		Naphthacemycin B1 **12**		Fasamycin C **13**	
**no.**	^{13}C	^{1}H, mult. (J,Hz)	^{13}C	^{1}H, mult. (J,Hz)	^{13}C	^{1}H, mult. (J,Hz)
1	101.4, CH	7.00, s	106.6, CH	6.66, d (2.1)	106.1, CH	6.66, d (2.3)
2	161.5, C	-	166.1, C	-	165.9, C	-
3	108.3, C	-	101.2, CH	6.21, d (2.1)	101.2, CH	6.21, d (2.0)
4	161.8, C	-	165.5, C	-	165.5, C	-
5	108.3, C	-	108.8, C	-	108.8, C	-
6	190.6, C	-	190.7, C	-	190.4, C	-
7	107.4, C	-	107.4, C	-	107.4, C	-
8	165.6, C	-	165.2, C	-	165.2, C	-
9	118.0, C	-	116.7, C	-	116.7, C	-
10	137.4, C	-	141.0, C	-	141.0, C	-
11	116.3, CH	7.09, s	121.7, CH	6.72, d (2.2)	121.7, CH	6.72, d (2.1)
12	156.2, C	-	158.8, C	-	158.8, C	-
13	116.1, C	-	109.3, CH	7.06, d (2.5)	109.3, CH	7.06, d (2.0)
14	141.4, C	-	141.6, C	-	141.6, C	-
15	111.3, CH	8.00, s	115.3, CH	7.36, s	115.3, CH	7.37, s
16	148.6, C	-	141.5 C	-	141.5 C	-
17	39.3, C	-	38.7, C	-	38.7, C	-
18	152.4, C	-	156.3, C	-	156.3, C	-
19	33.5, CH_3	1.83, s	34.0, CH_3	1.71, s	33.8, CH_3	1.72, s
20	33.5, CH_3	1.82, s	34.0, CH_3	1.70, s	33.8, CH_3	1.70, s
21	124.9, C	-	124.4 C	-	124.4 C	-
22	152.4, C	-	155.1, C	-	155.1, C	-
23	107.6, CH	-	100.1, CH	6.24, d (2.1)	100.1, CH	6.32, s
24	148.6, C	-	156.4, C	-	159.3, C	-
25	113.1, C	-	107.9, CH	6.27 ,d (2.3)	107.9, CH	6.37 , s
26	132.4, C	-	136.8, C	-	136.8, C	-
27	17.1, CH_3	1.99, s	19.8, CH_3	1.87, s	19.8, CH_3	1.92, s
28	-	-	-	-	54.0, CH_3	3.80, s
29	55.9, CH_3	4.02, s	-	-	-	-
30	55.7, CH_3	4.06, s	-	-	-	-

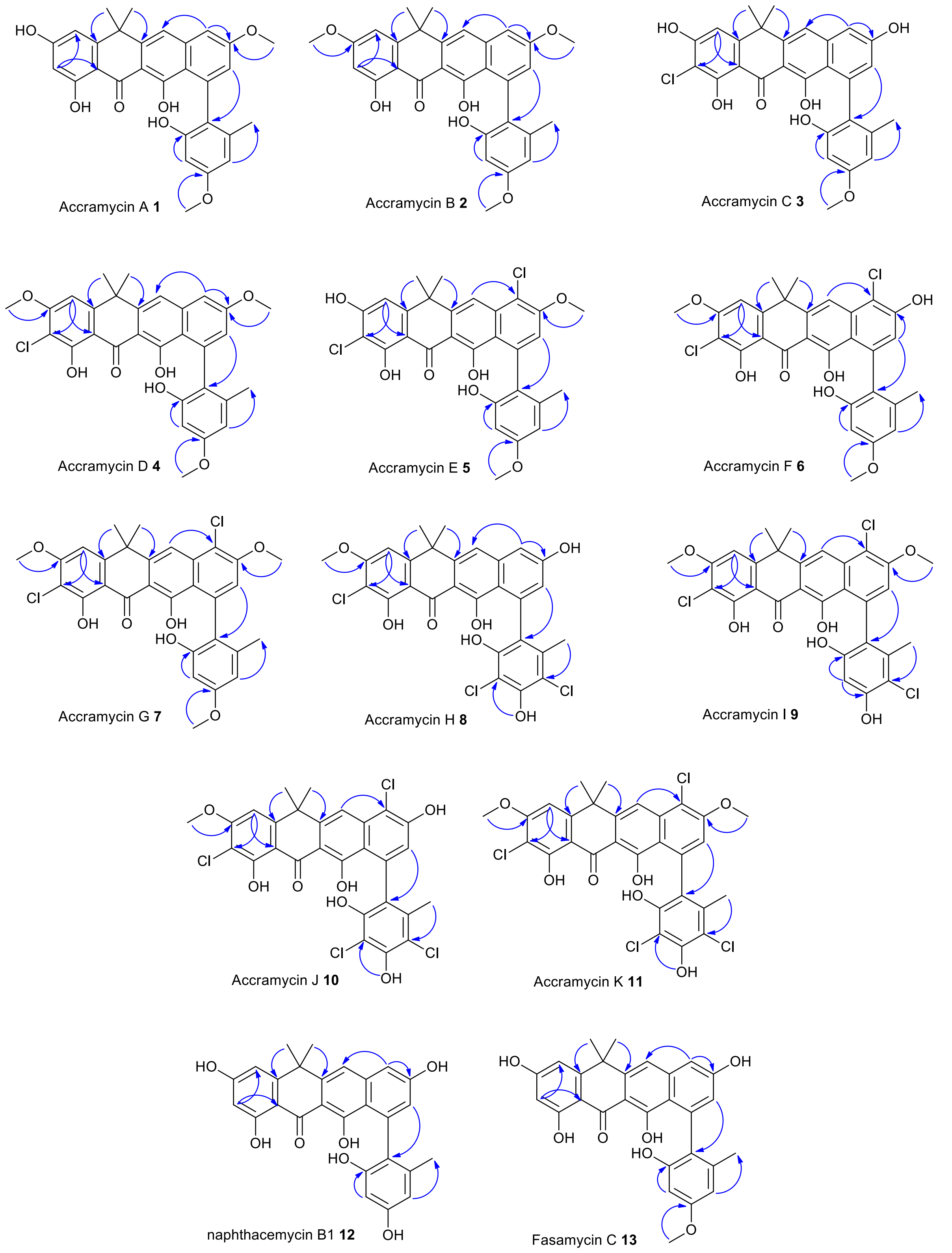

Figure S2. Key HMBC correlations (→) of accramycins A-K **1-11**, naphthacemycin B1 **12**, and fasamycin C **13** (CD_3OD, 298K, 600MHz)

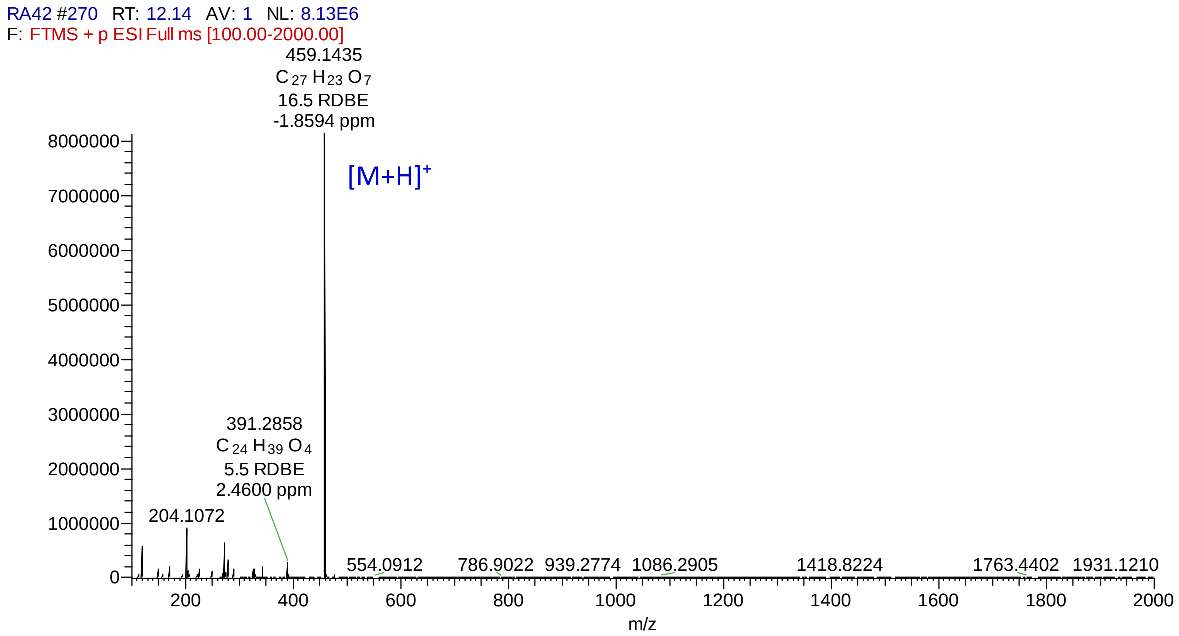

Figure S3. HRESIMS of naphthacemycin B1 **12**

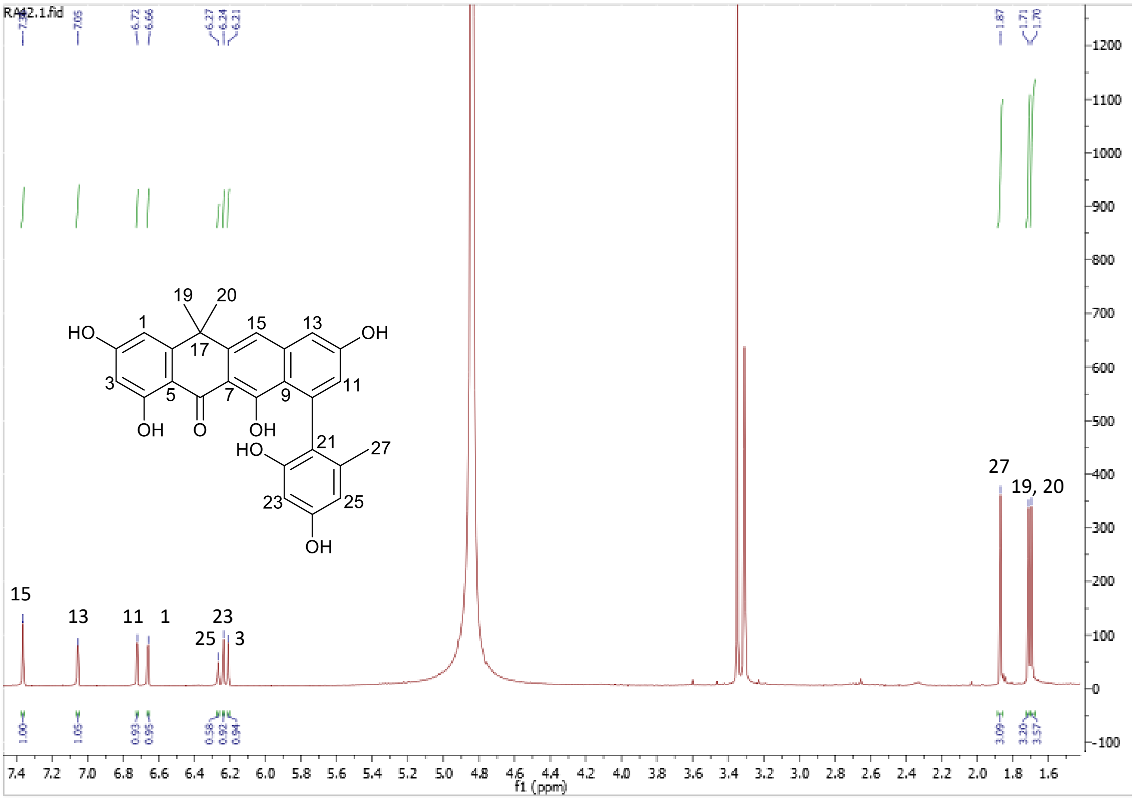

Figure S4. ^{1}H-NMR of naphthacemycin B1 **12** (CD_3OD, 298K, 600MHz)

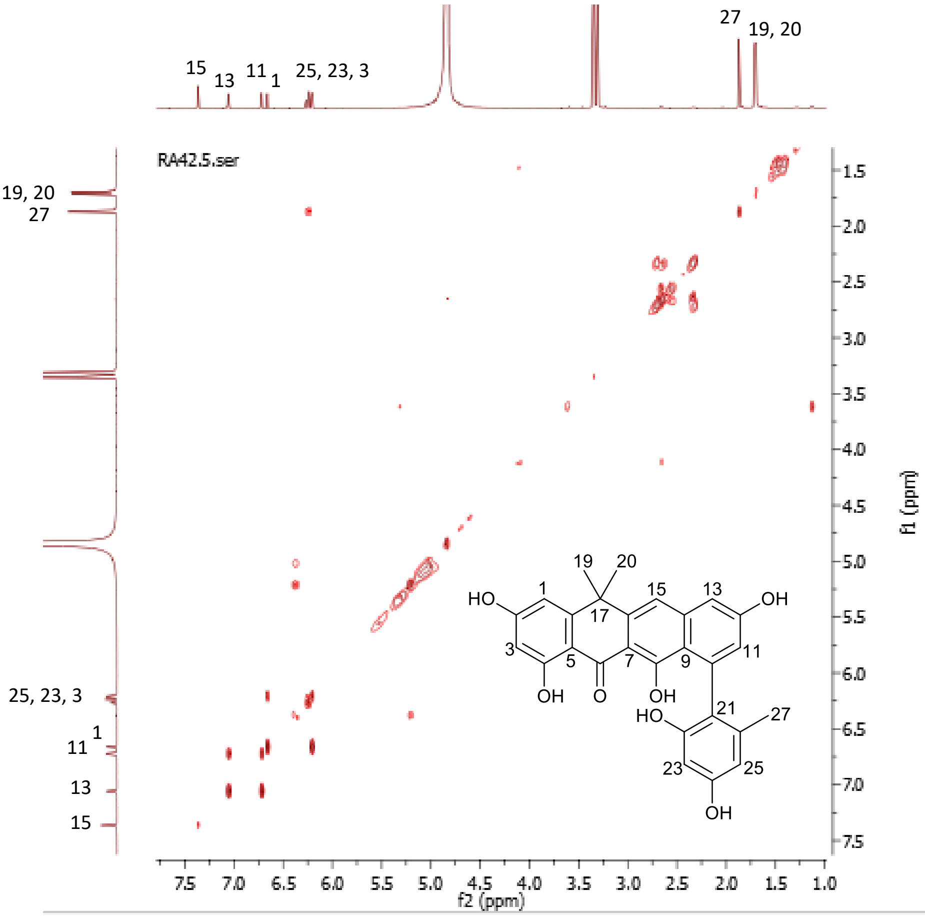

Figure S5. ^{1}H-^{1}H COSY of naphthacemycin B1 **12** (CD_3OD, 298K, 600MHz)

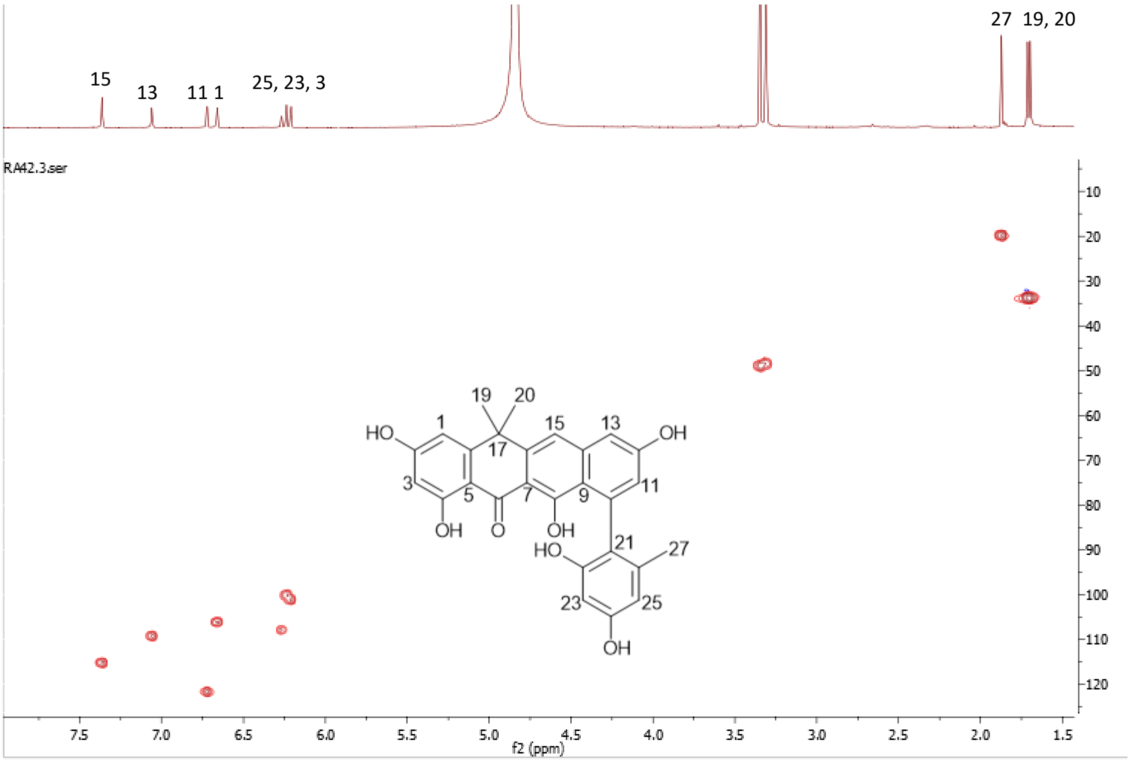

Figure S6. HSQC of naphthacemycin B1 **12** (CD_3OD, 298K, 600MHz)

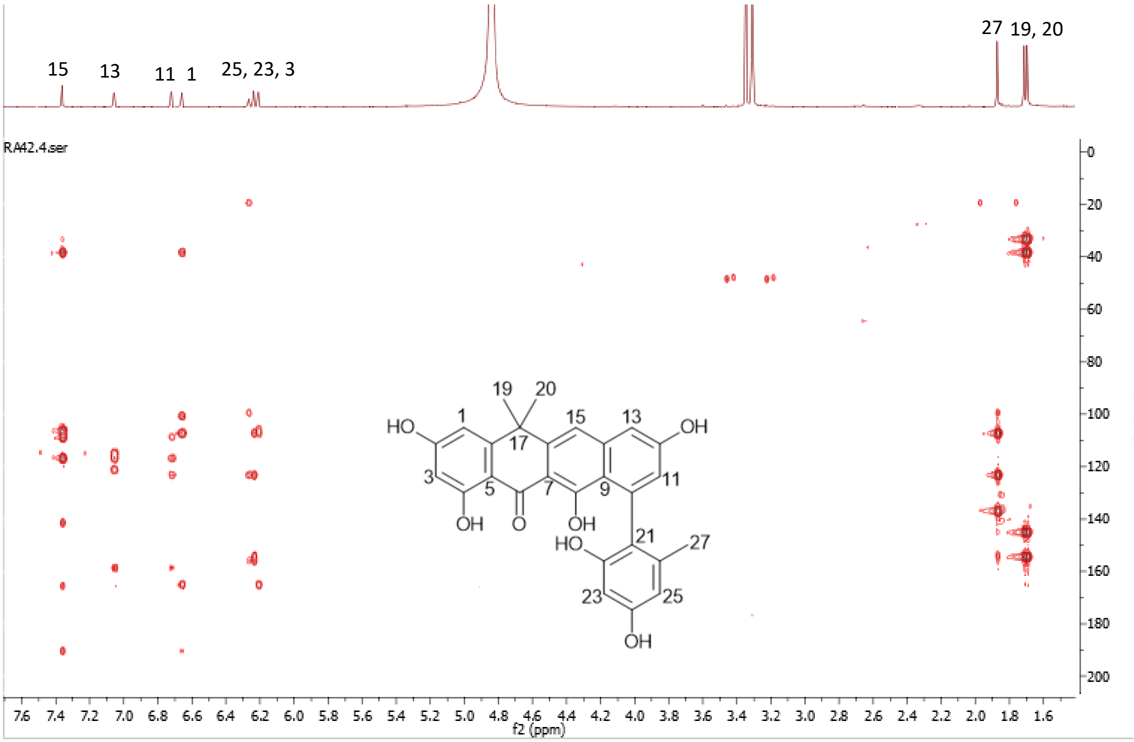

Figure S7. HMBC of naphthacemycin B1 **12** (CD_3OD, 298K, 600MHz)

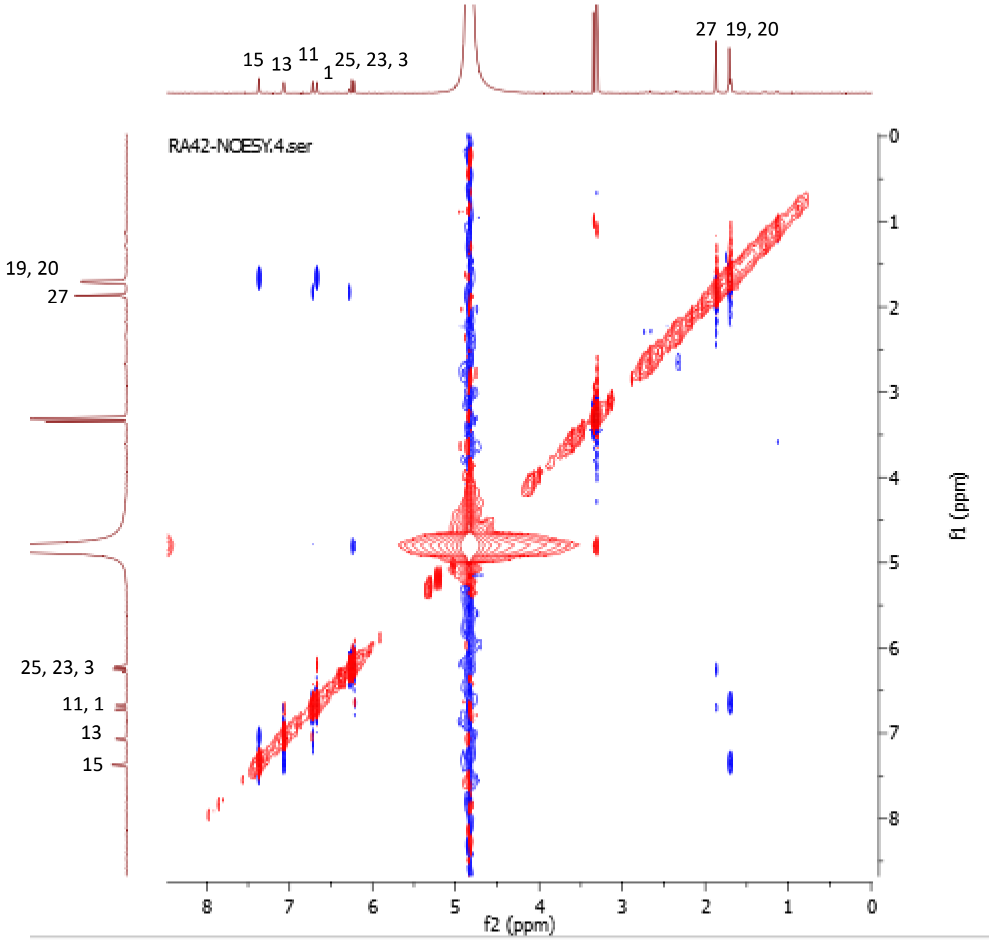

Figure S8. NOESY of naphthacemycin B1 **12** (CD_3OD, 298K, 600MHz)

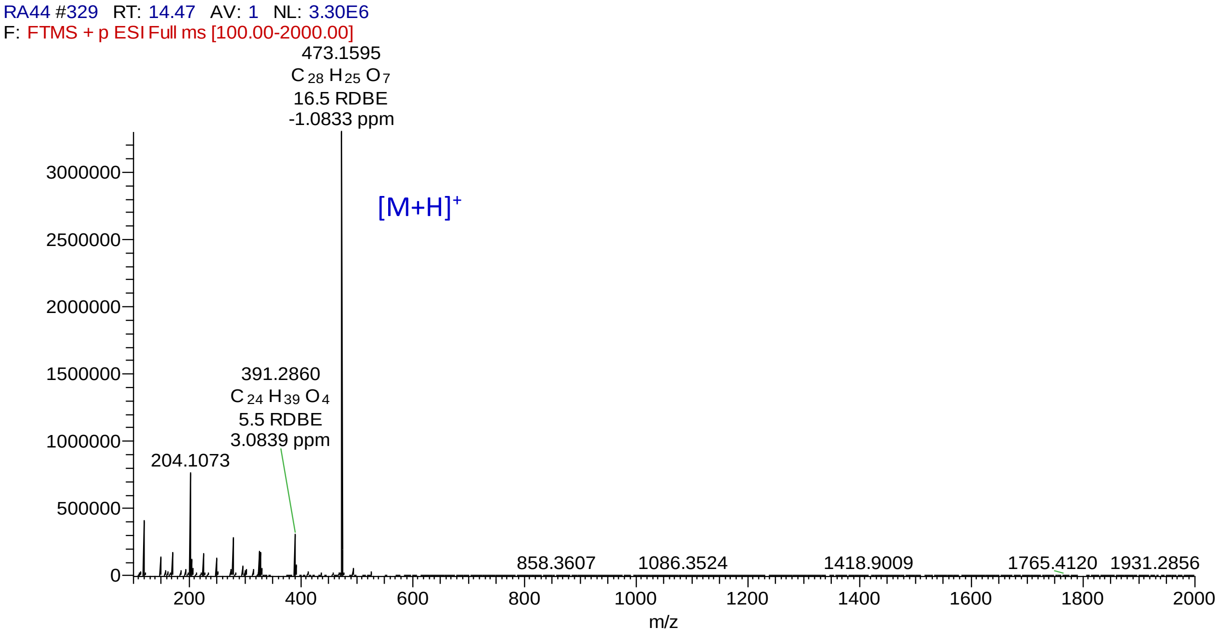

Figure S9. HRESIMS of fasamycin C **13**

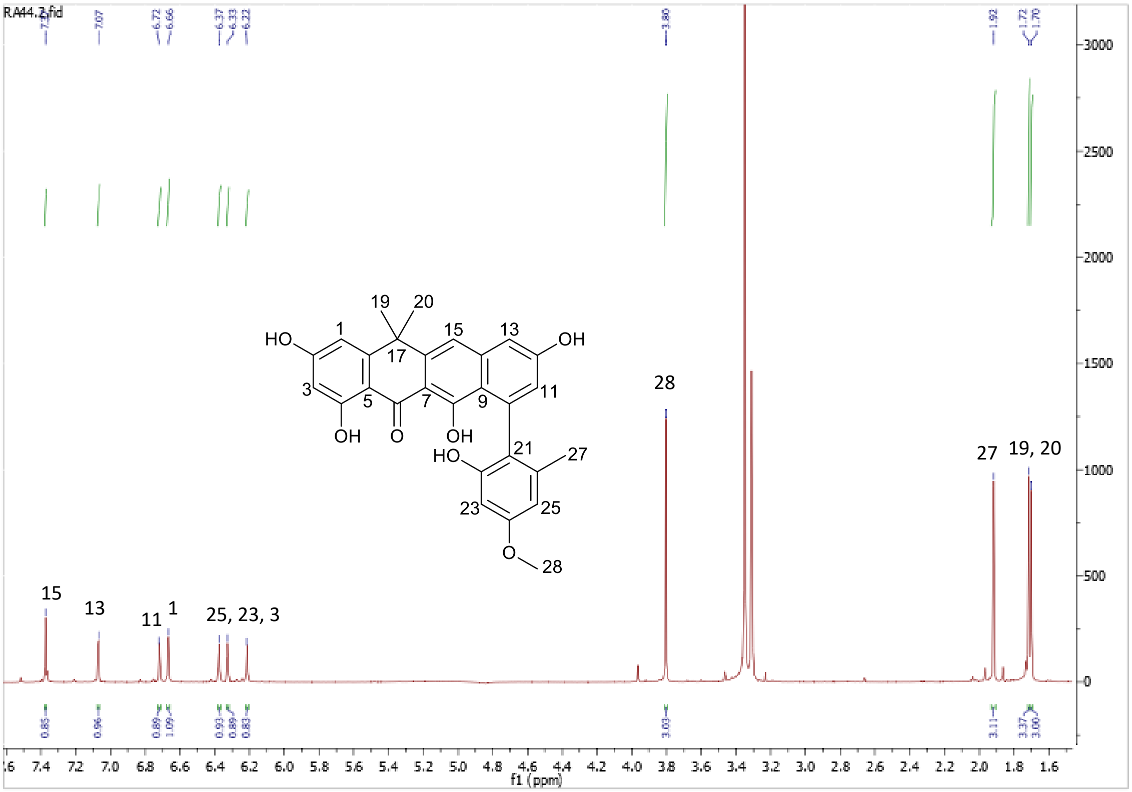

Figure S10. ^{1}H-NMR of fasamycin C **13** (CD_3OD, 298K, 600MHz)

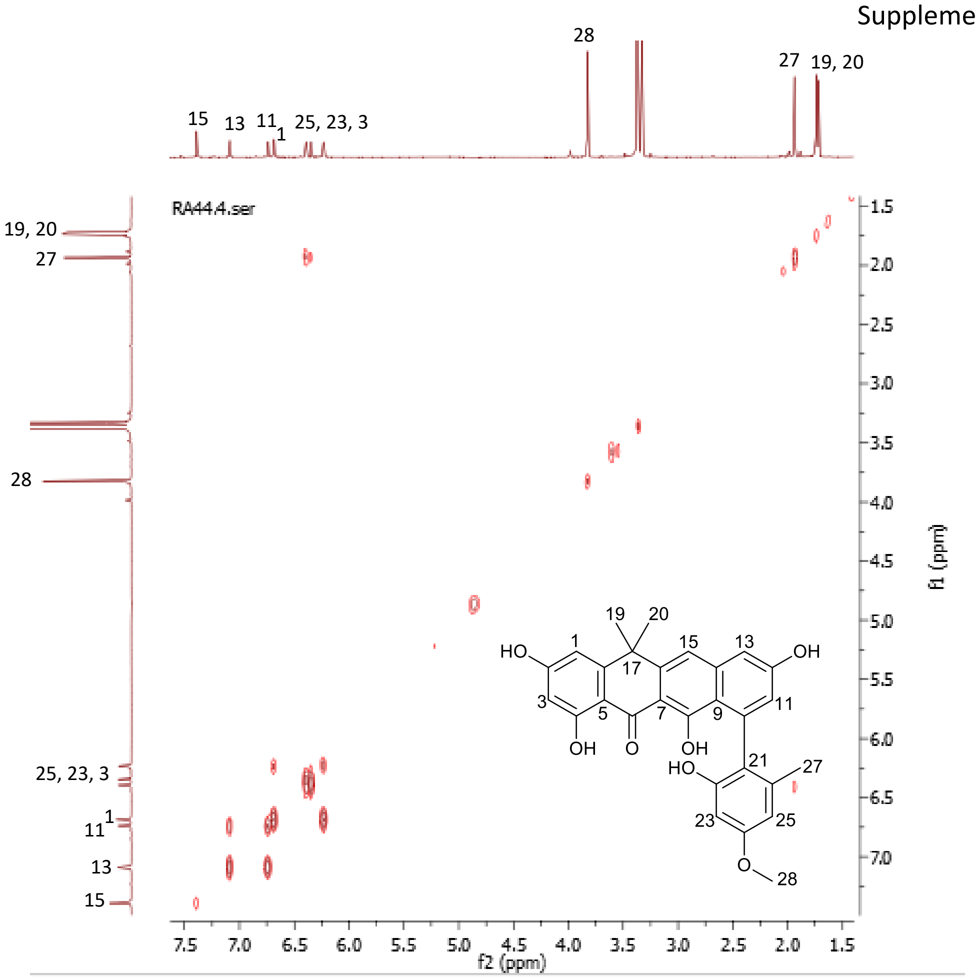

Figure S11. ^{1}H-^{1}H COSY of fasamycin C **13** (CD_3OD, 298K, 600MHz)

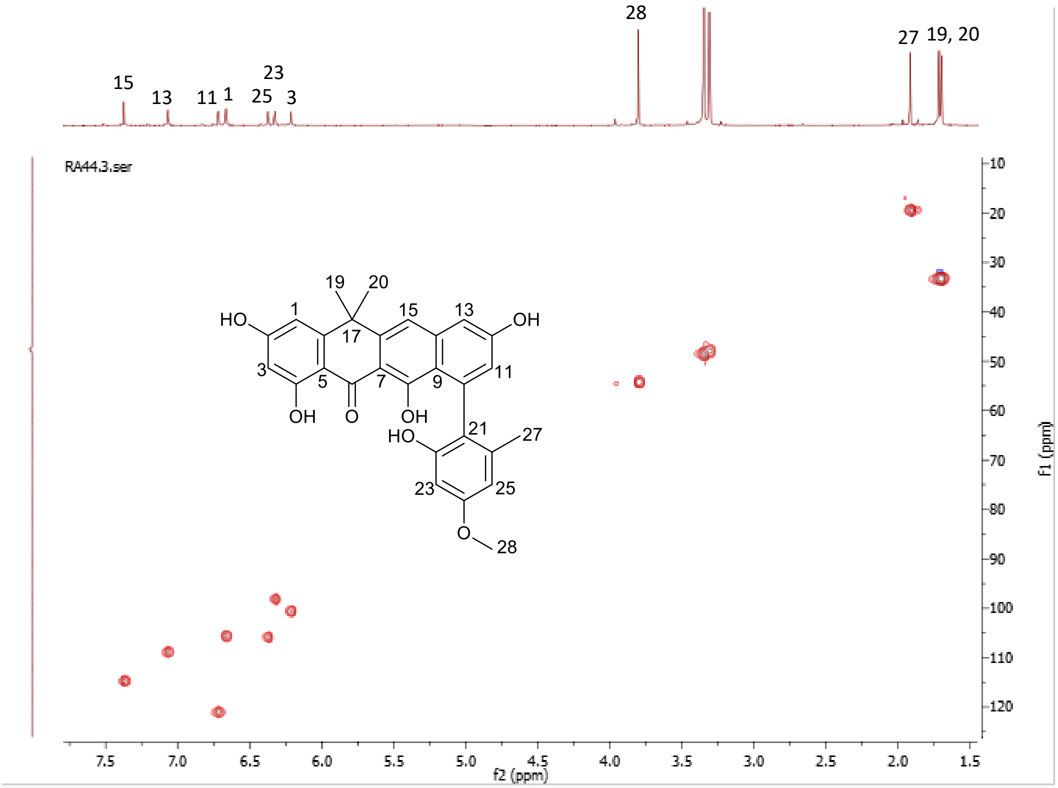

Figure S12. HSQC of fasamycin C **13** (CD_3OD, 298K, 600MHz)

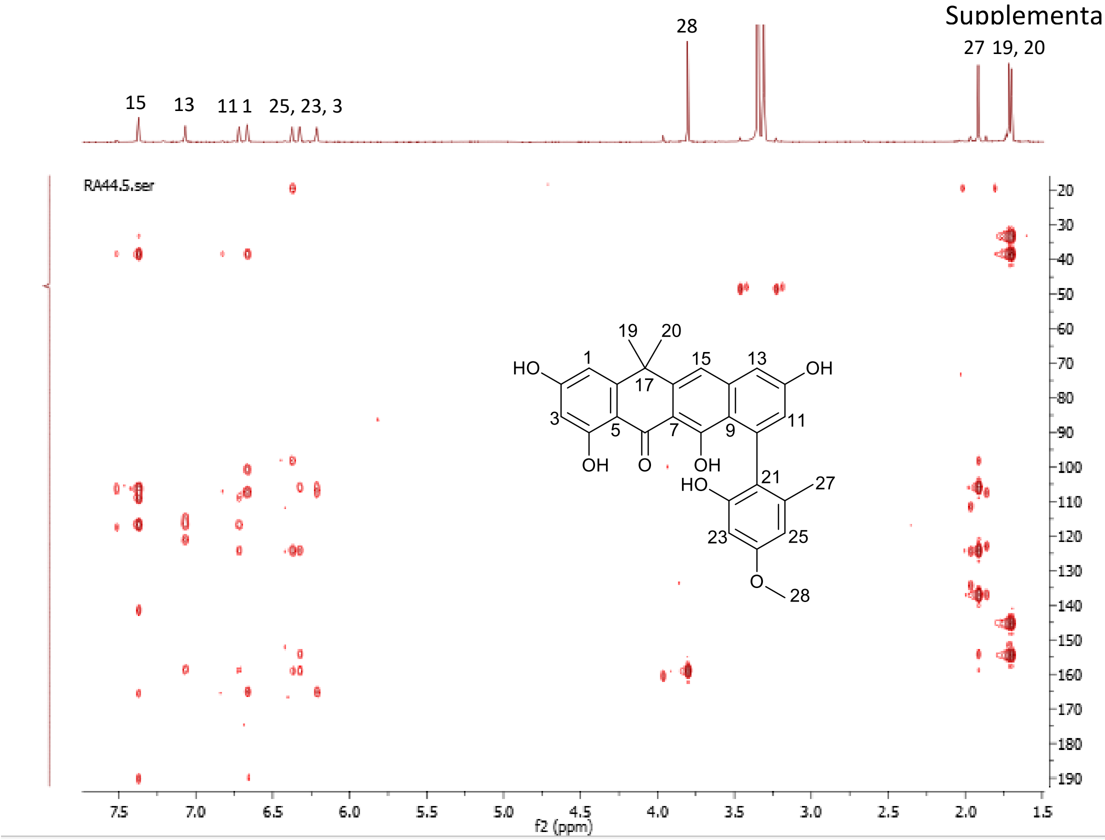

Figure S13. HMBC of fasamycin C **13** (CD_3OD, 298K, 600MHz)

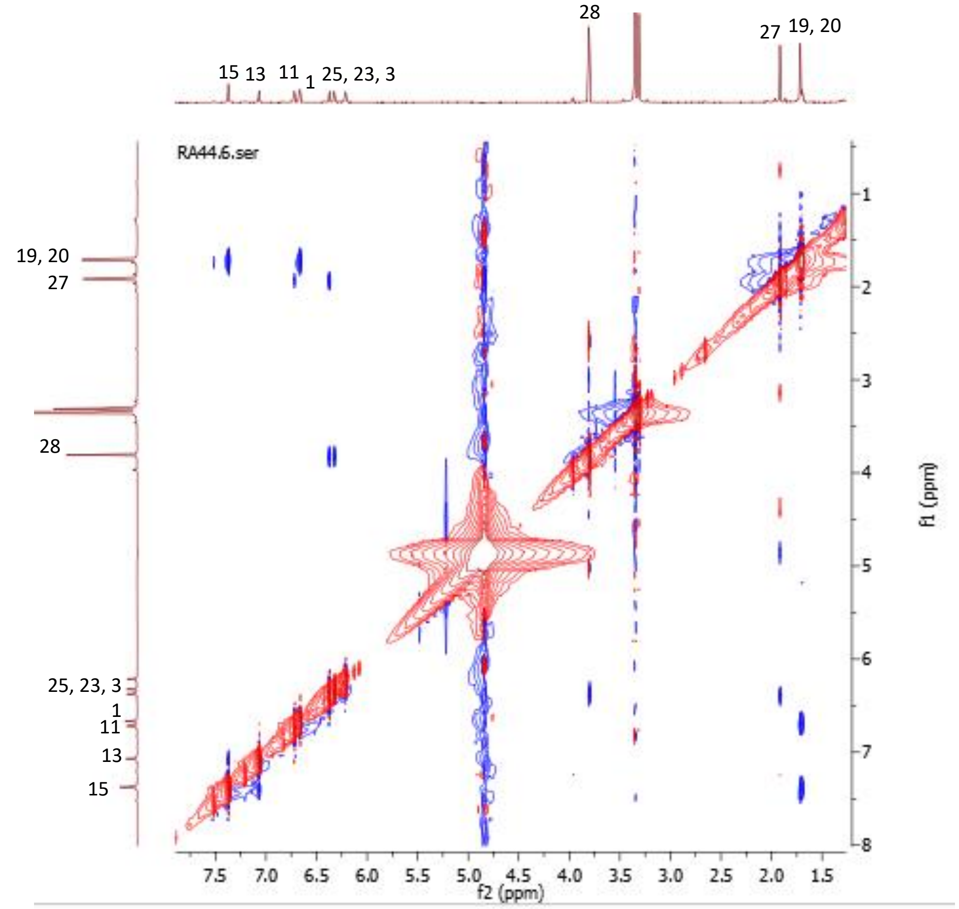

Figure S14. NOESY of fasamycin C **13** (CD_3OD, 298K, 600MHz)

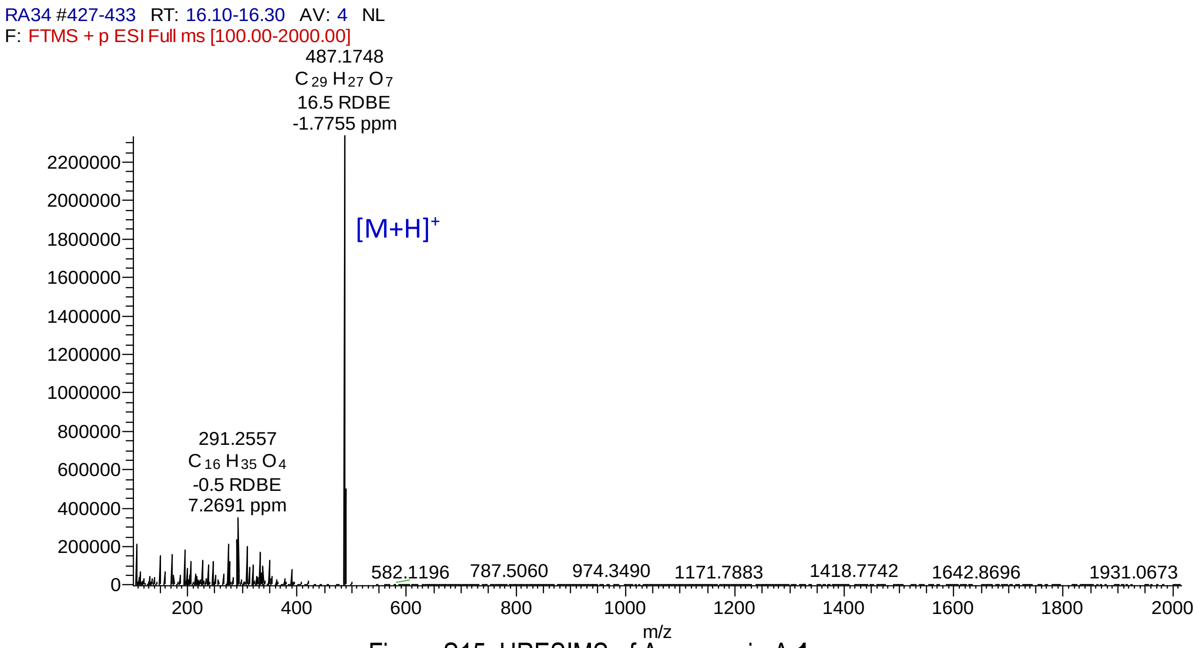

Figure S15. HRESIMS of Accramycin A **1**

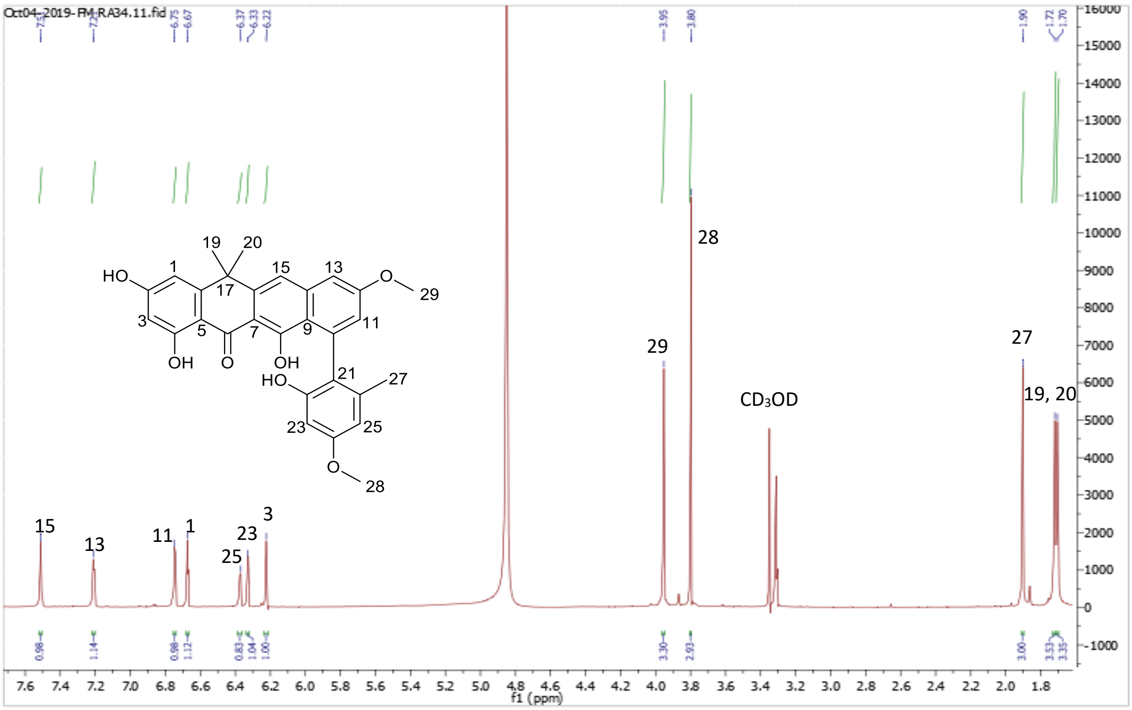

Figure S16. ^{1}H-NMR of Accramycin A **1** (CD_3OD, 298K, 600MHz)

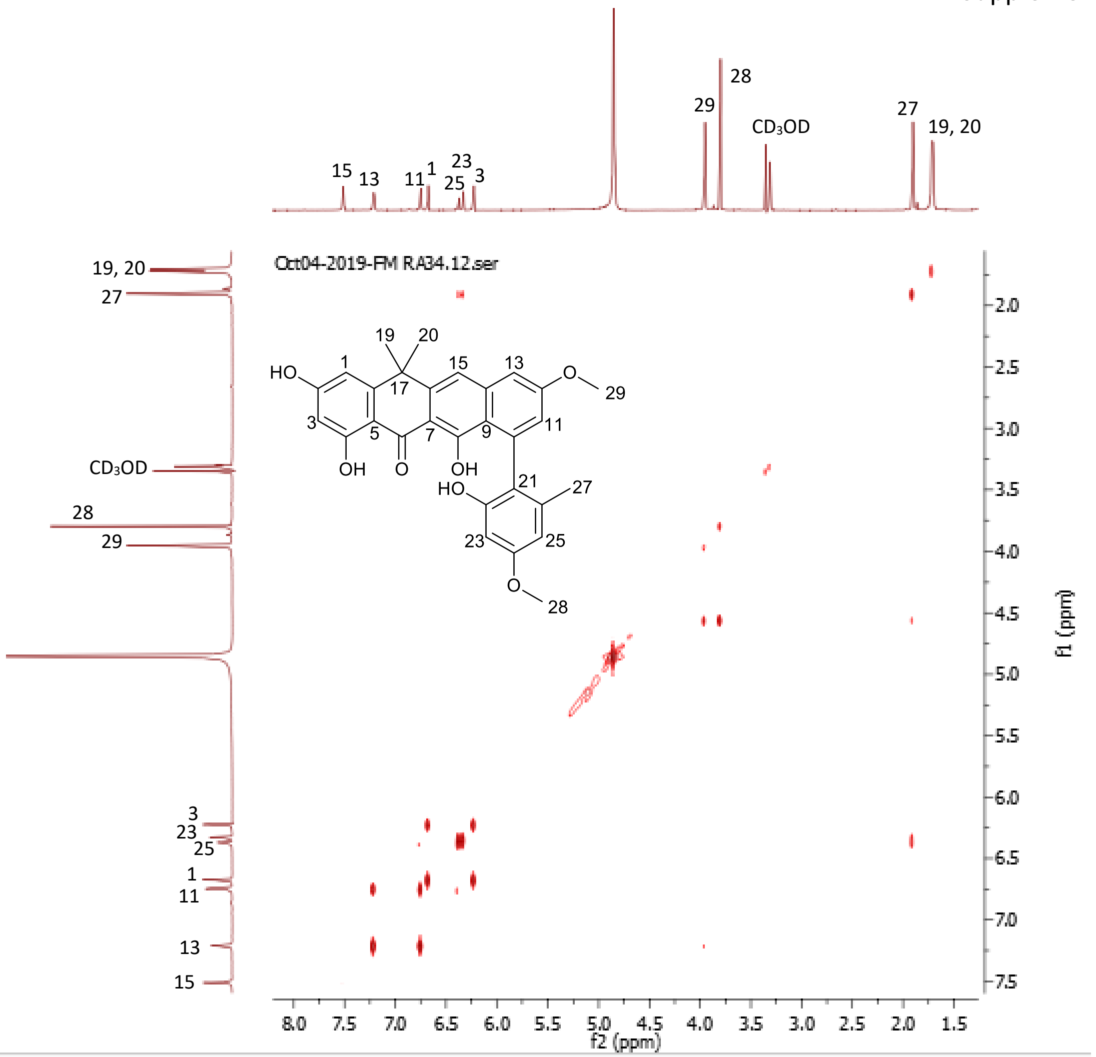

Figure S17. ^1H-^1H COSY NMR of Accramycin A **1** (CD_3OD, 298K, 600MHz)

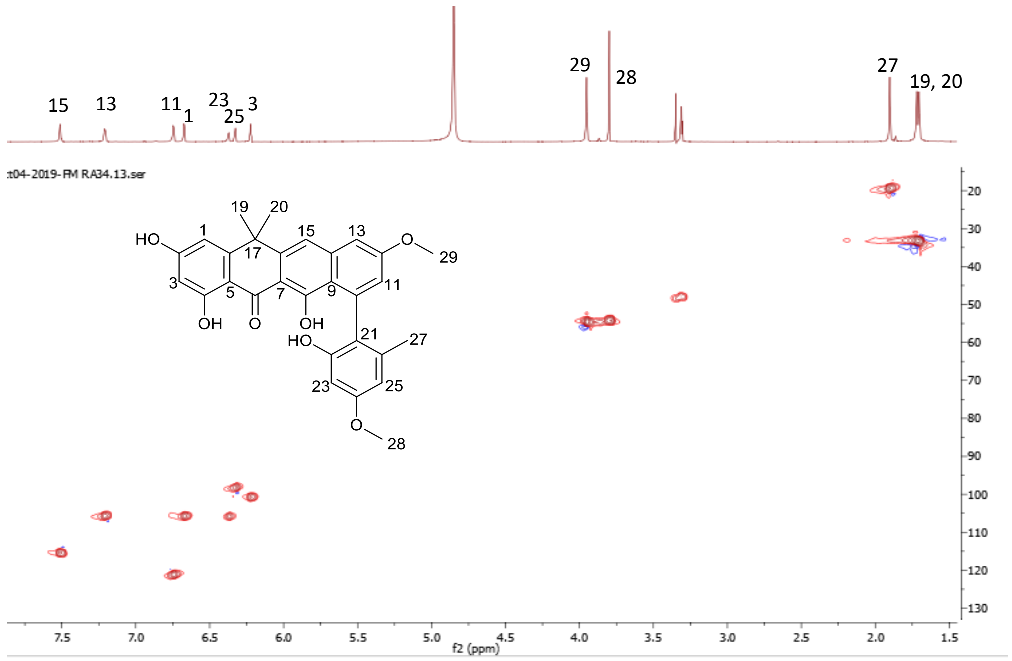

Figure S18. HSQC of Accramycin A **1** (CD_3OD, 298K, 600MHz)

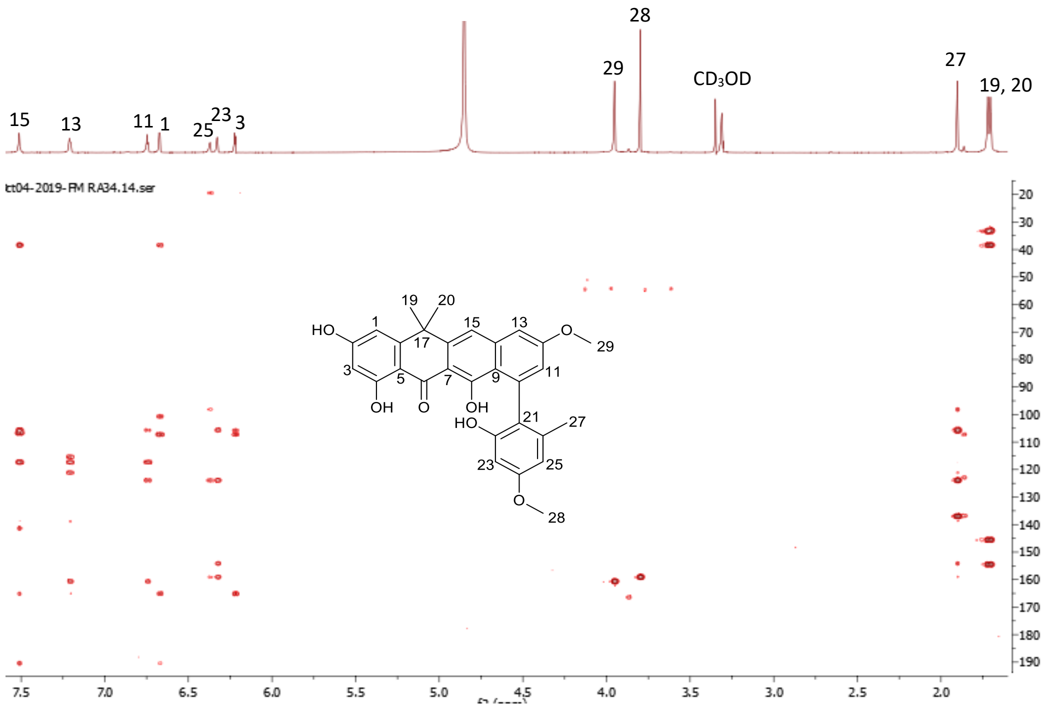

Figure S19. HMBC of Accramycin A **1** (CD_3OD, 298K, 600MHz)

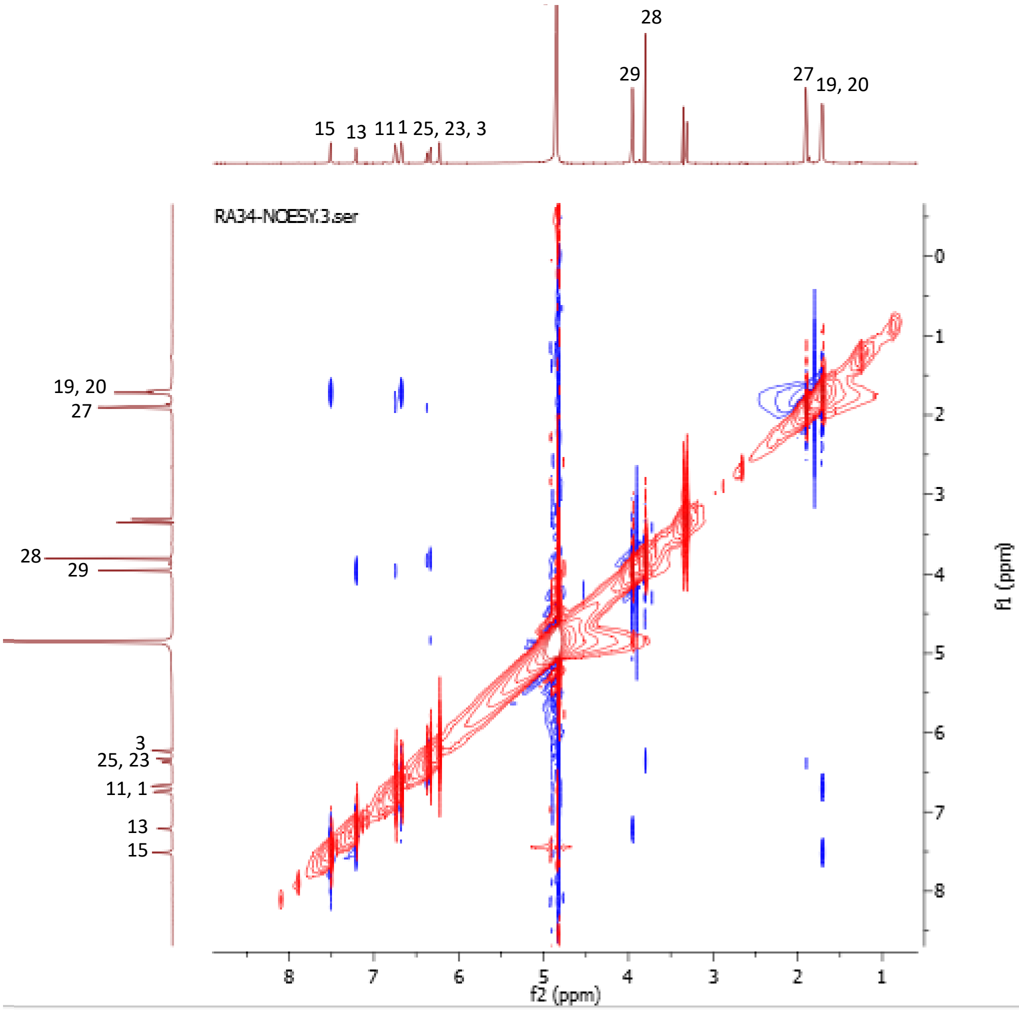

Figure S20. NOESY of Accramycin A **1** (CD_3OD, 298K, 600MHz)

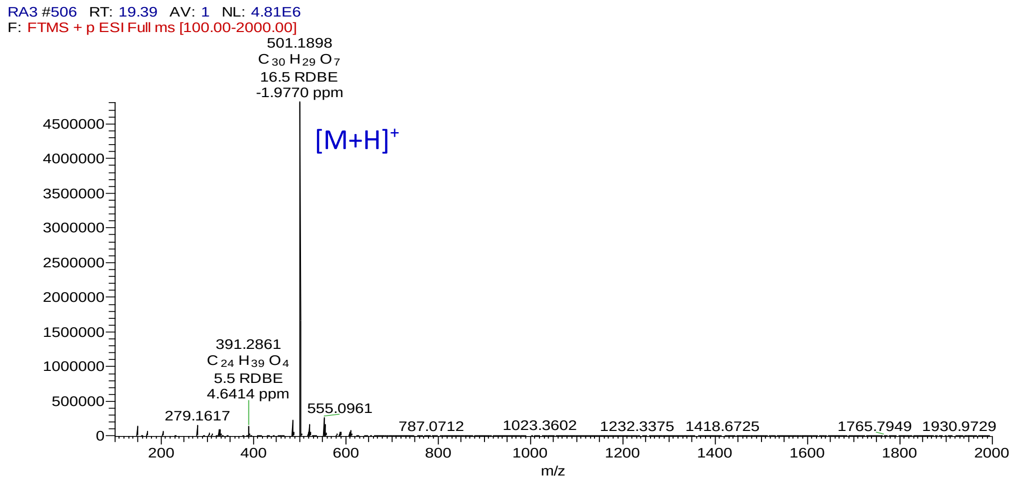

Figure S21. HRESIMS of Accramycin B **2**

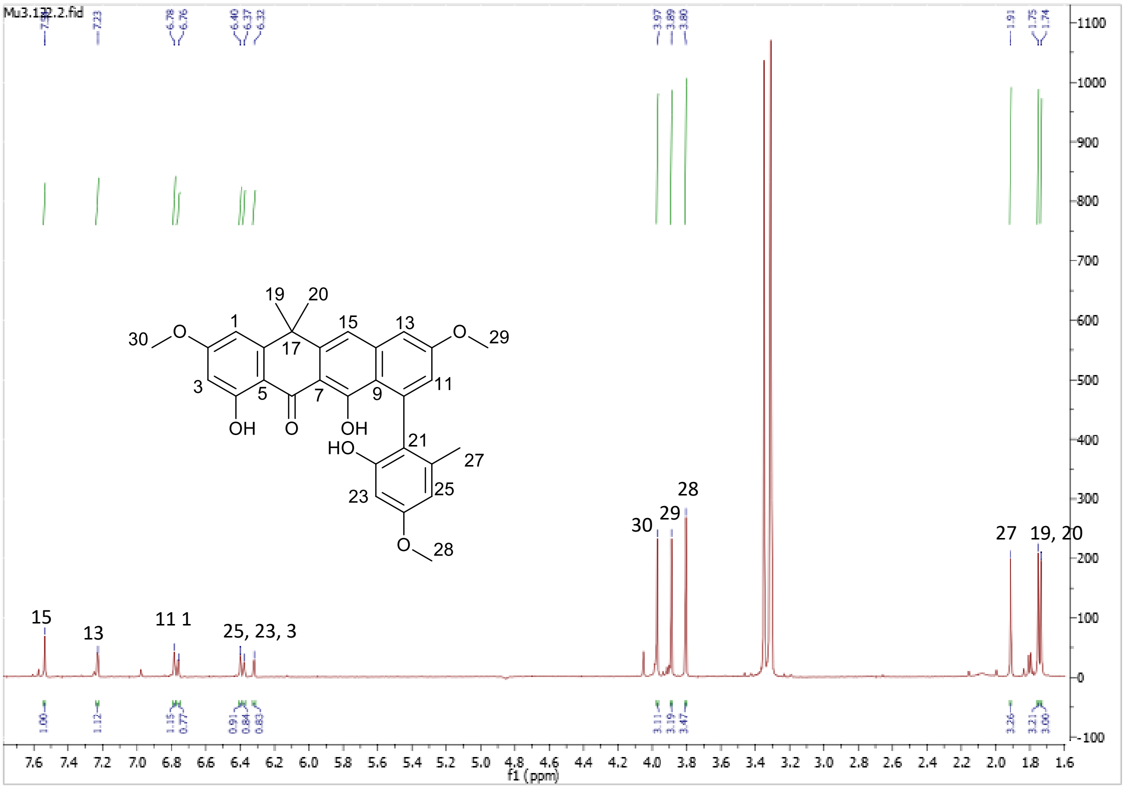

Figure S22. ^{1}H-NMR of Accramycin B **2** (CD_3OD, 298K, 600MHz)

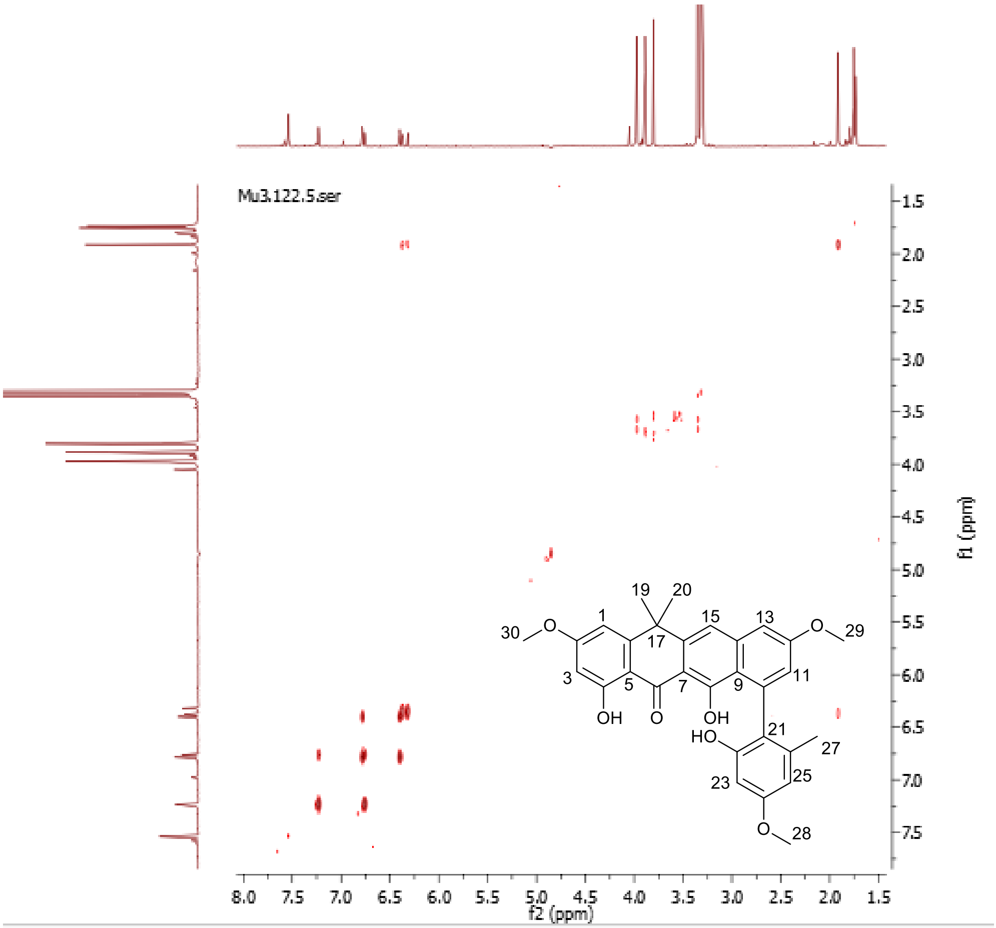

Figure S23. COSY of Accramycin B **2** (CD_3OD, 298K, 600MHz)

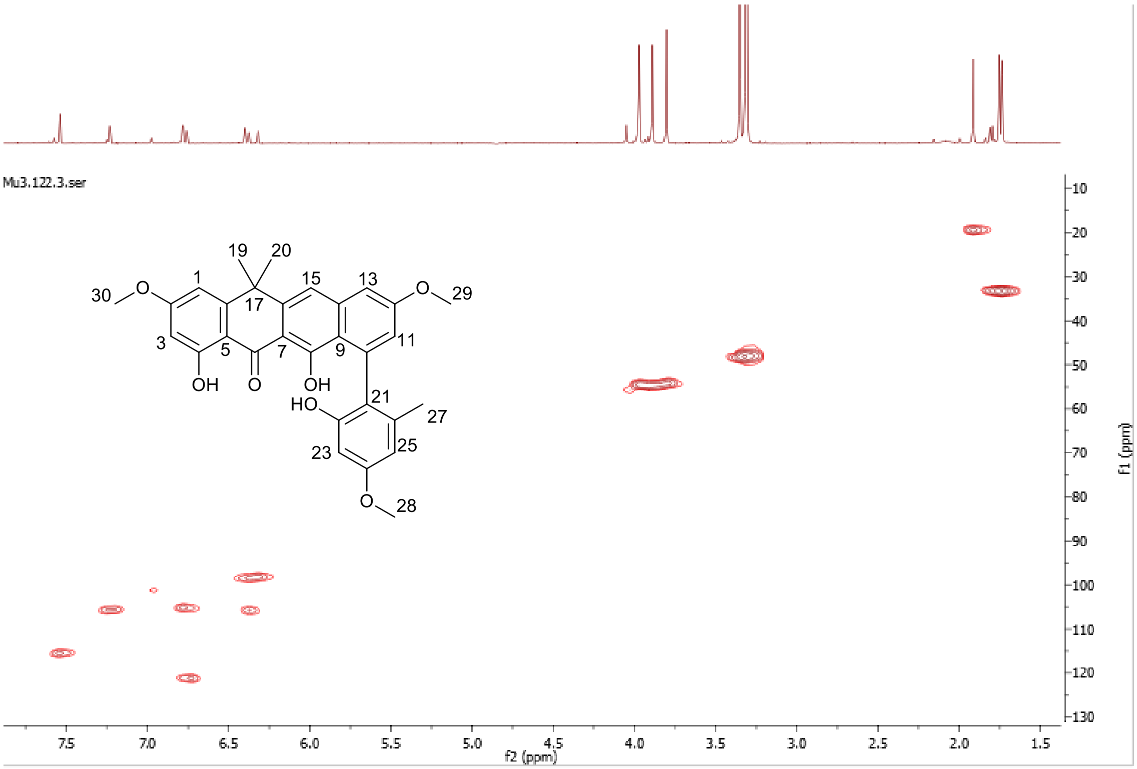

Figure S24. HSQC of Accramycin B **2** (CD_3OD, 298K, 600MHz)

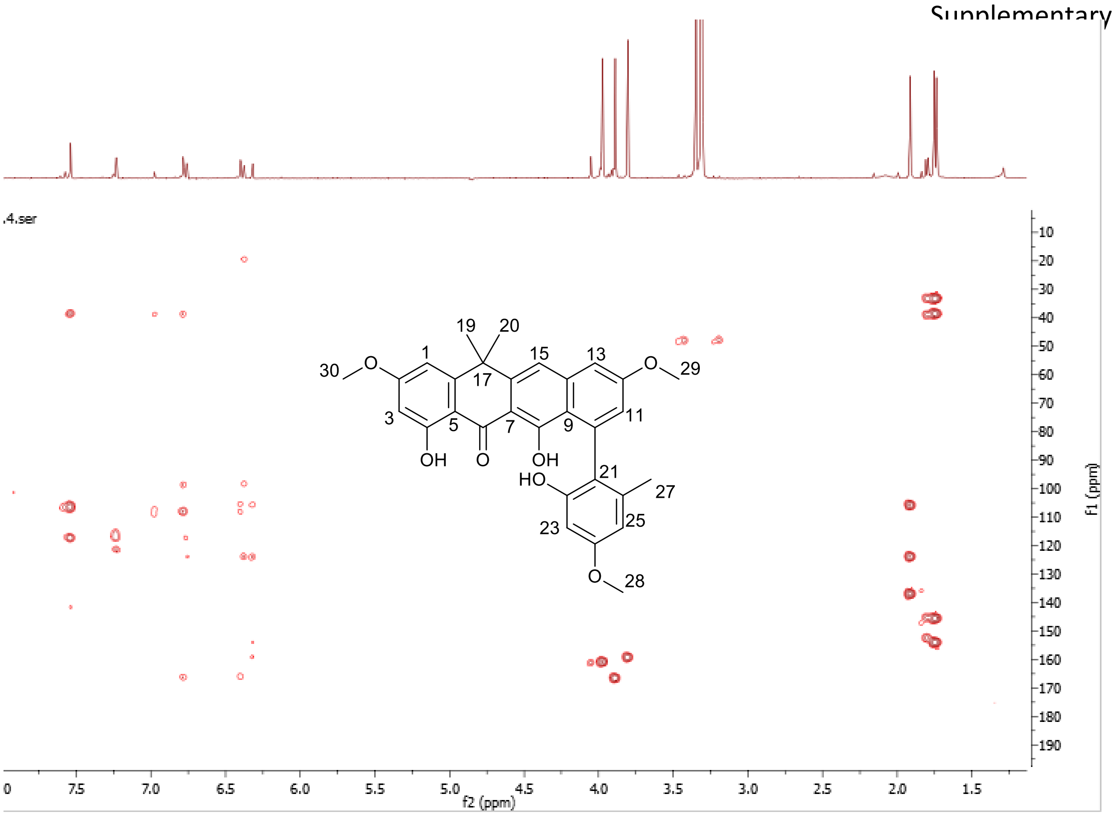

Figure S25. HMBC of Accramycin B **2** (CD_3OD, 298K, 600MHz)

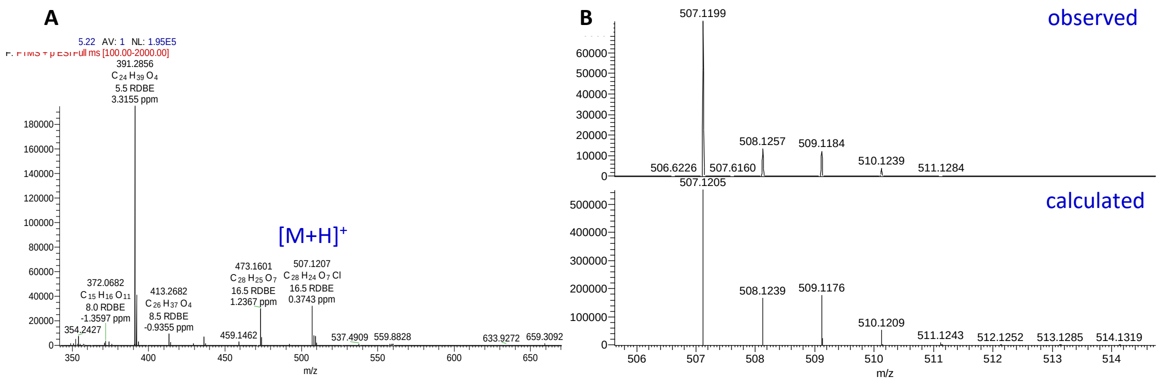

Figure S26. **A**. HRESIMS and **B.** Isotope Pattern of Accramycin C **3**

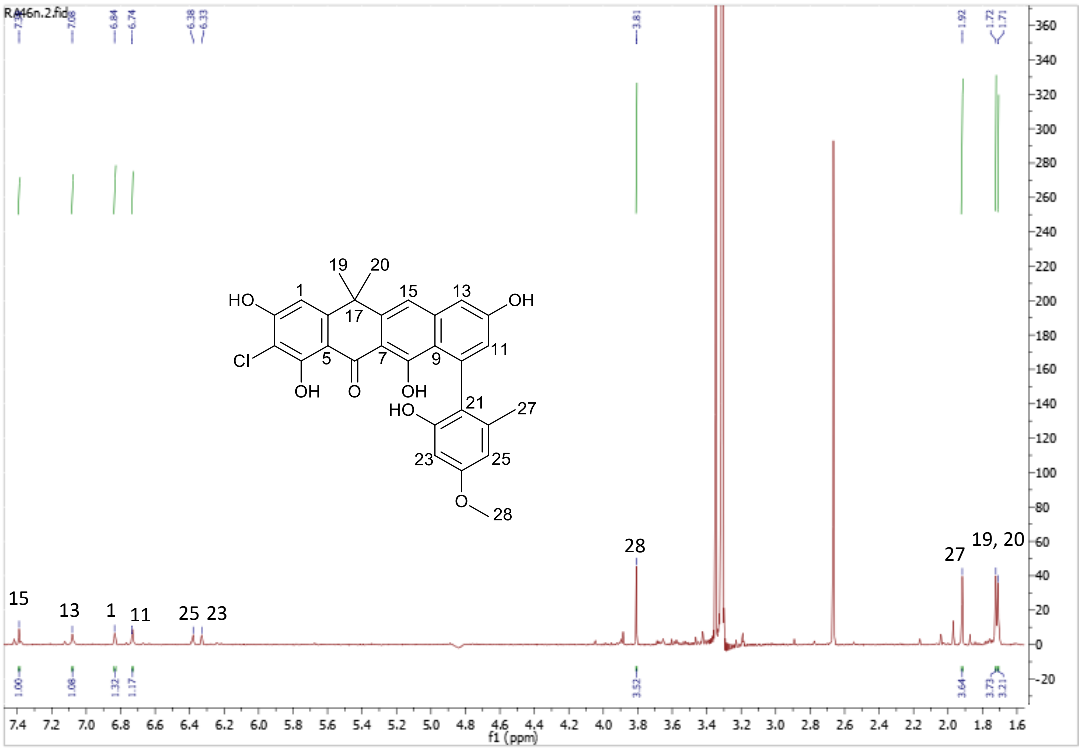

Figure S27. ^{1}H-NMR of Accramycin C **3** (CD_3OD, 298K, 600MHz)

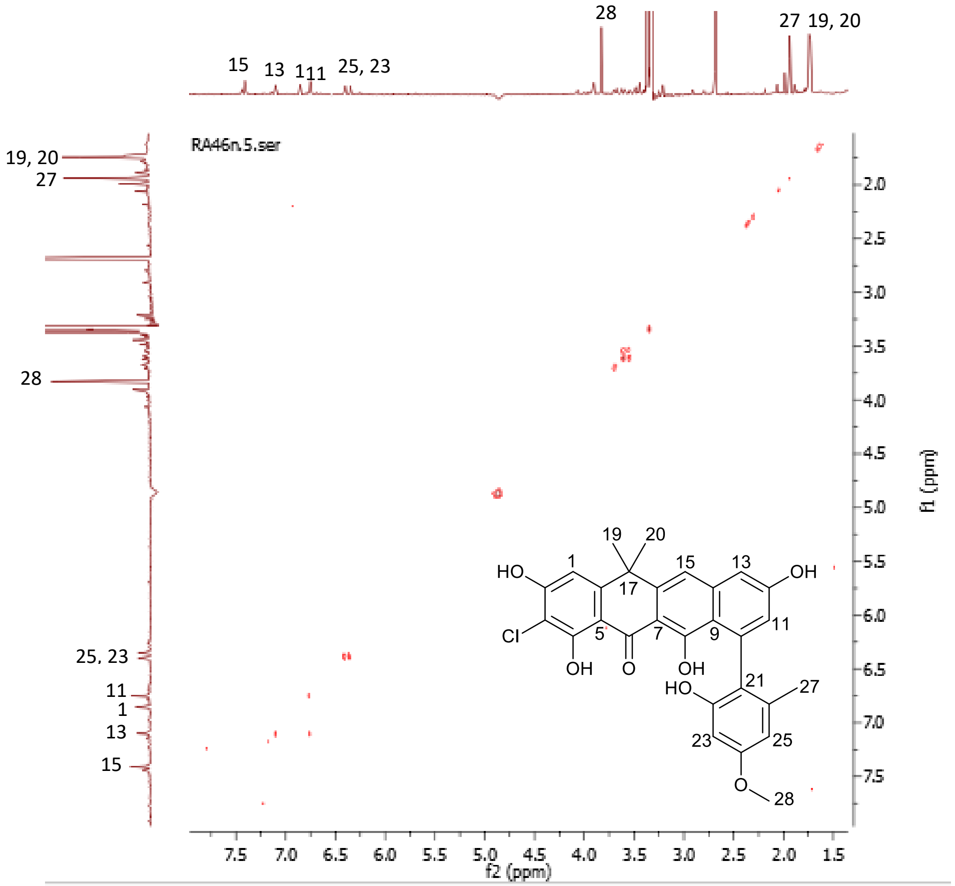

Figure S28. ^{1}H-^{1}H COSY of Accramycin C **3** (CD_3OD, 298K, 600MHz)

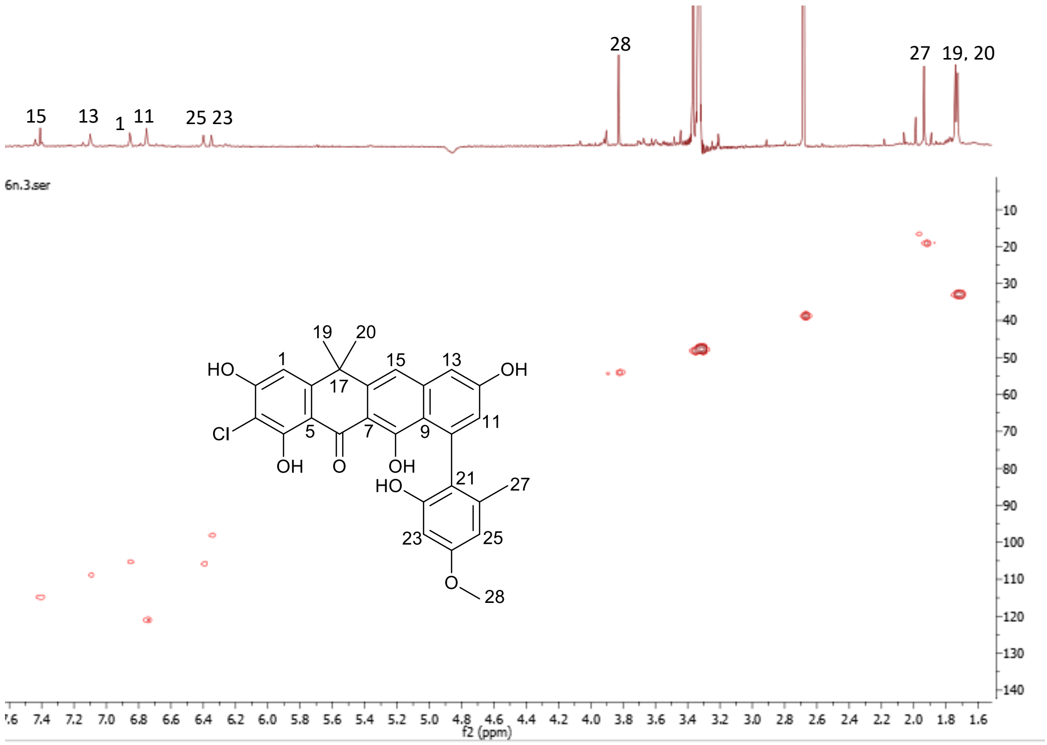

Figure S29. HSQC of Accramycin C **3** (CD_3OD, 298K, 600MHz)

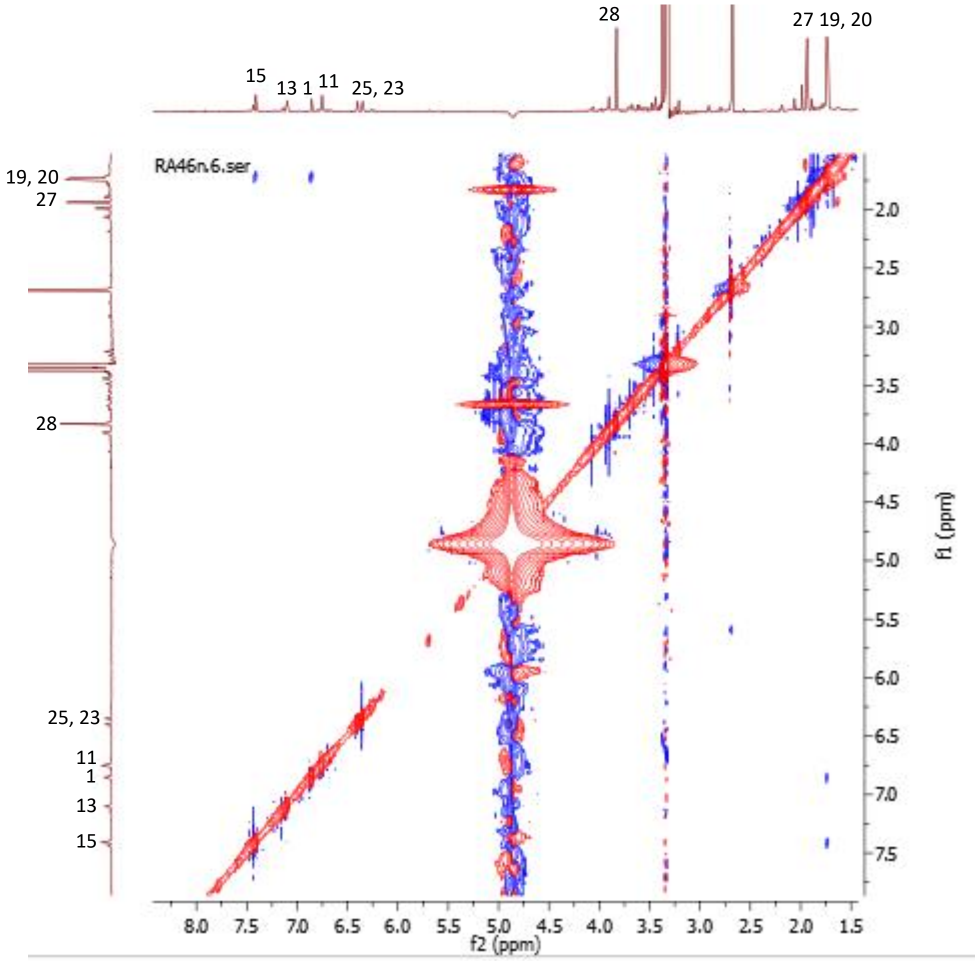

Figure S30. NOESY of Accramycin C **3** (CD_3OD, 298K, 600MHz)

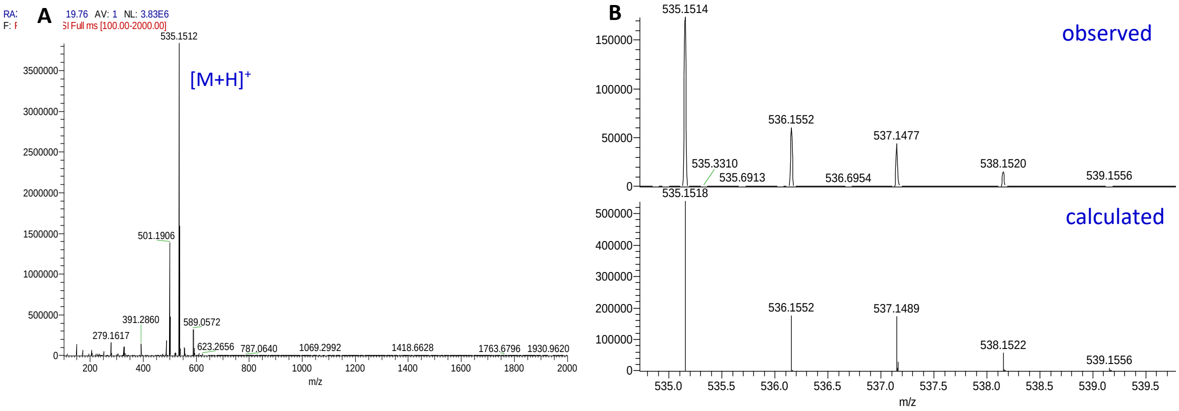

Figure S31. **A**. HRESIMS and **B**. Isotope Pattern of Accramycin D **4**

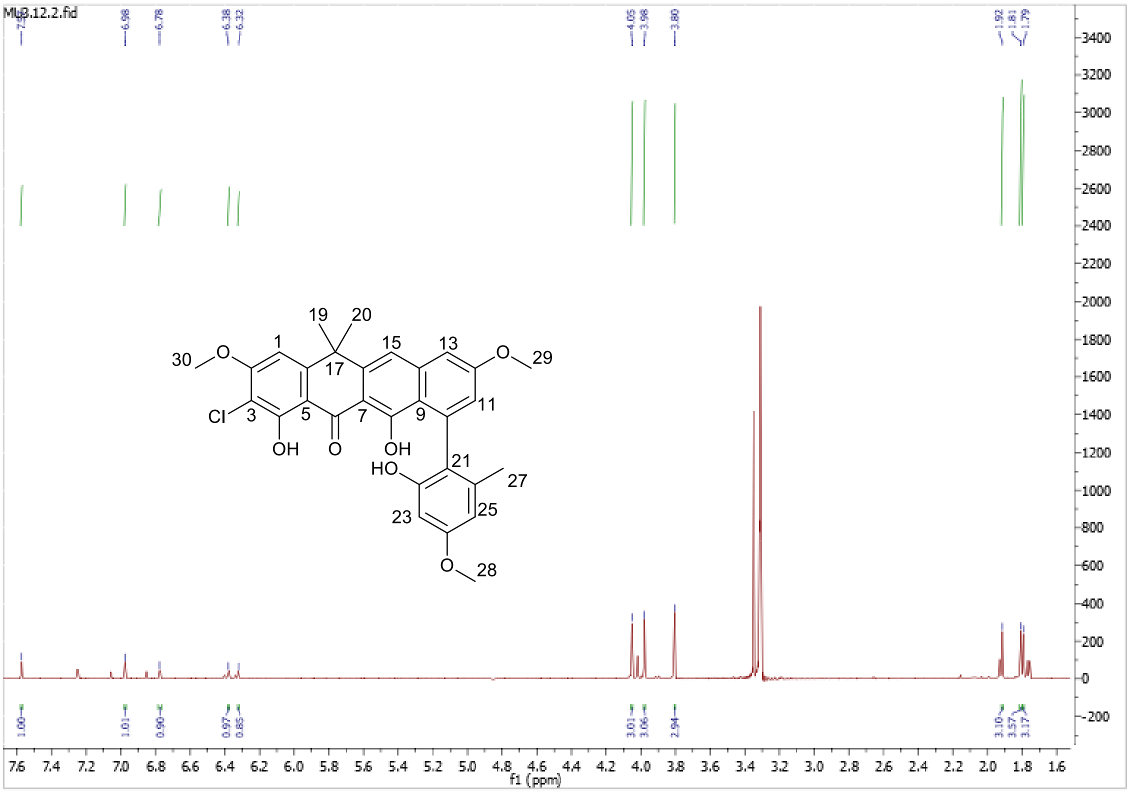

Figure S32. ^{1}H-NMR of Accramycin D **4** (CD_3OD, 298K, 600MHz)

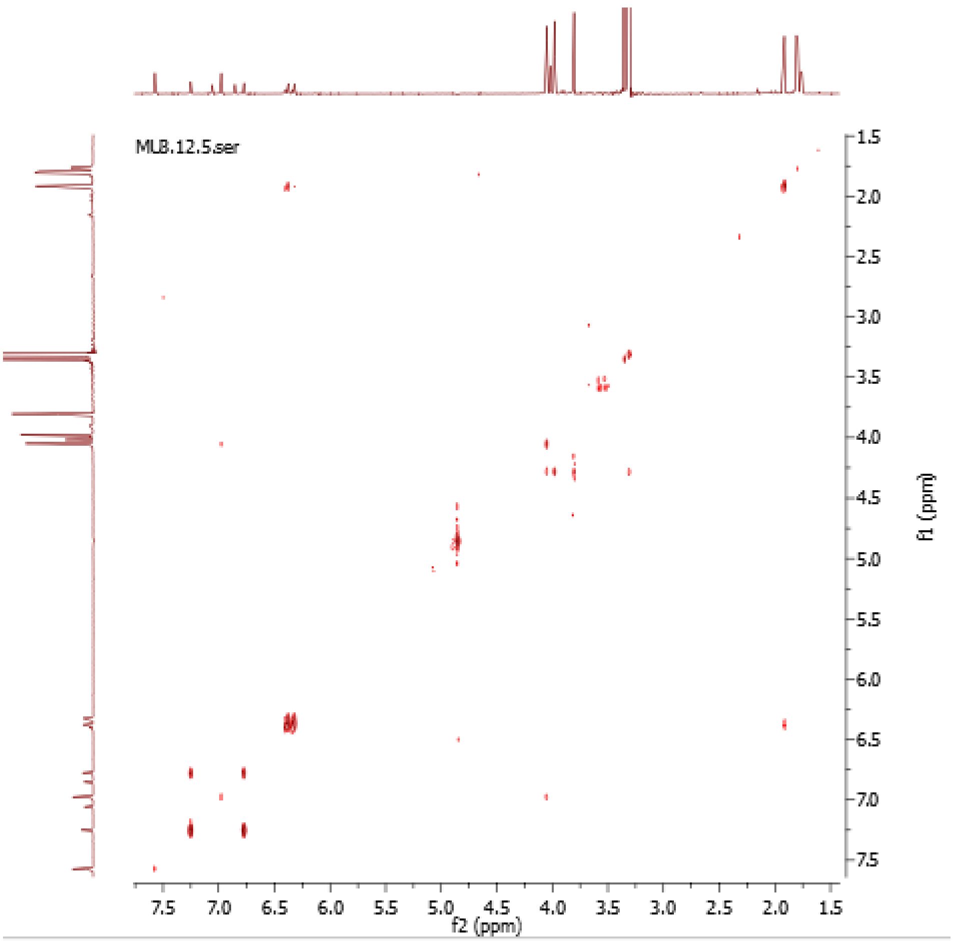

Figure S33. ^{1}H-^{1}H COSY of Accramycin D **4** (CD_3OD, 298K, 600MHz)

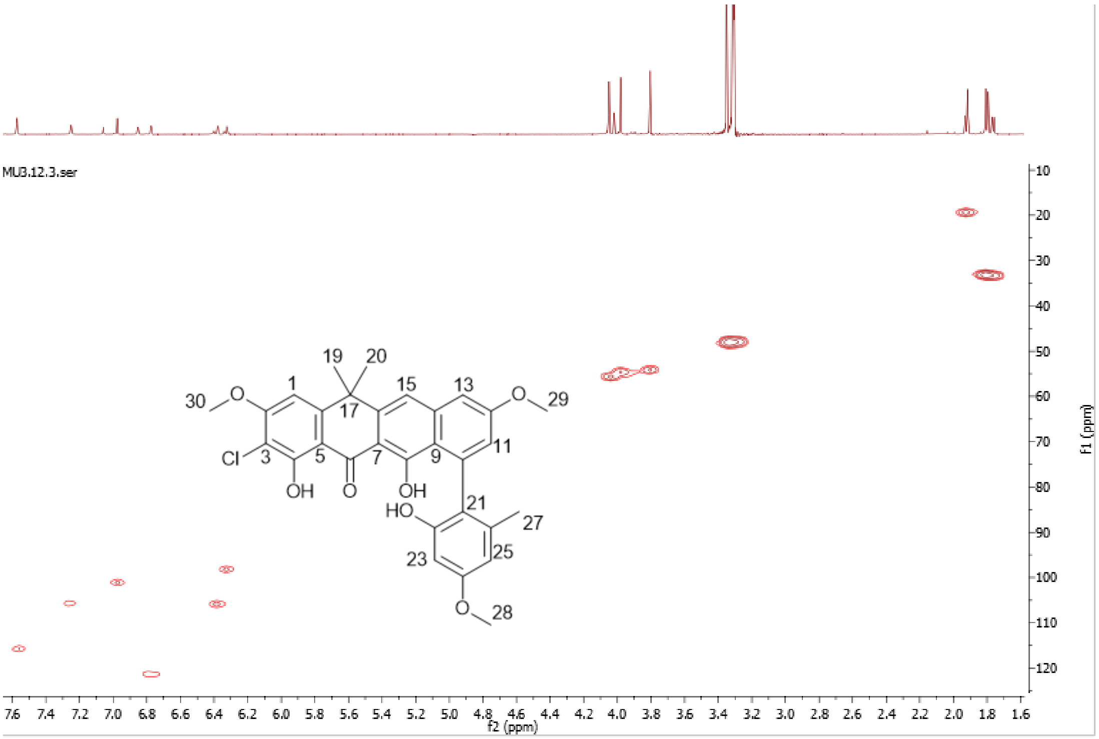

Figure S34. HSQC of Accramycin D **4** (CD_3OD, 298K, 600MHz)

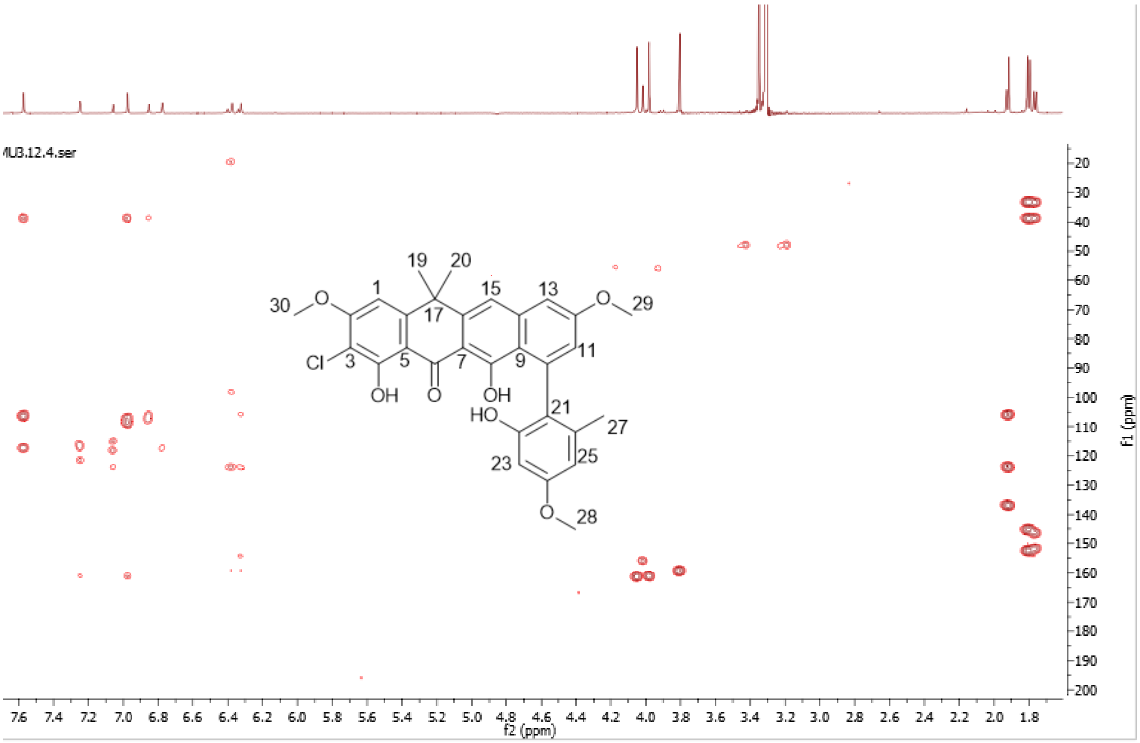

Figure S35. HMBC of Accramycin D **4** (CD_3OD, 298K, 600MHz)

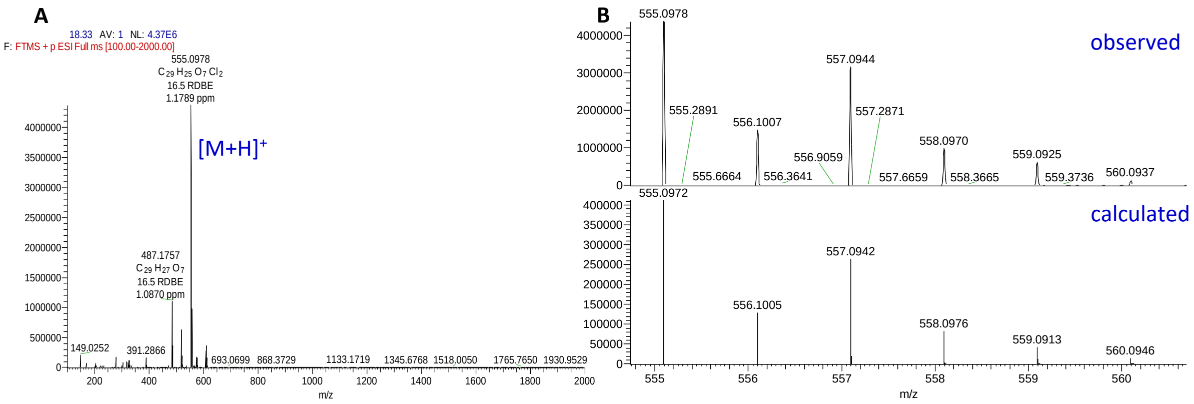

Figure S36. **A.** HRESIMS and **B.** Isotope Pattern of Accramycin E **5**

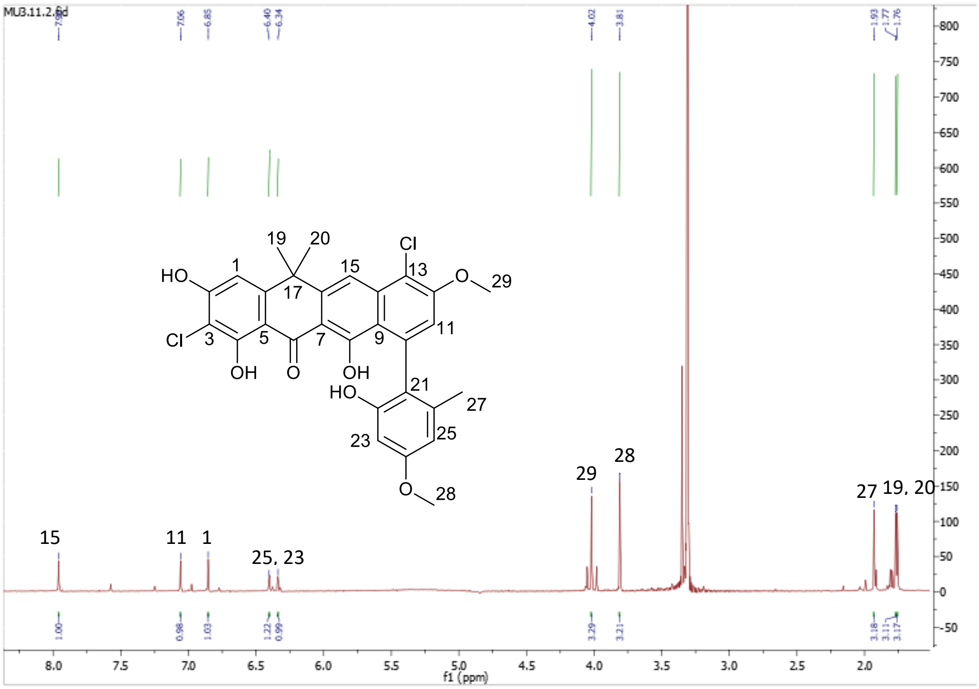

Figure S37. ^{1}H-NMR Accramycin E **5** (CD_3OD, 298K, 600MHz)

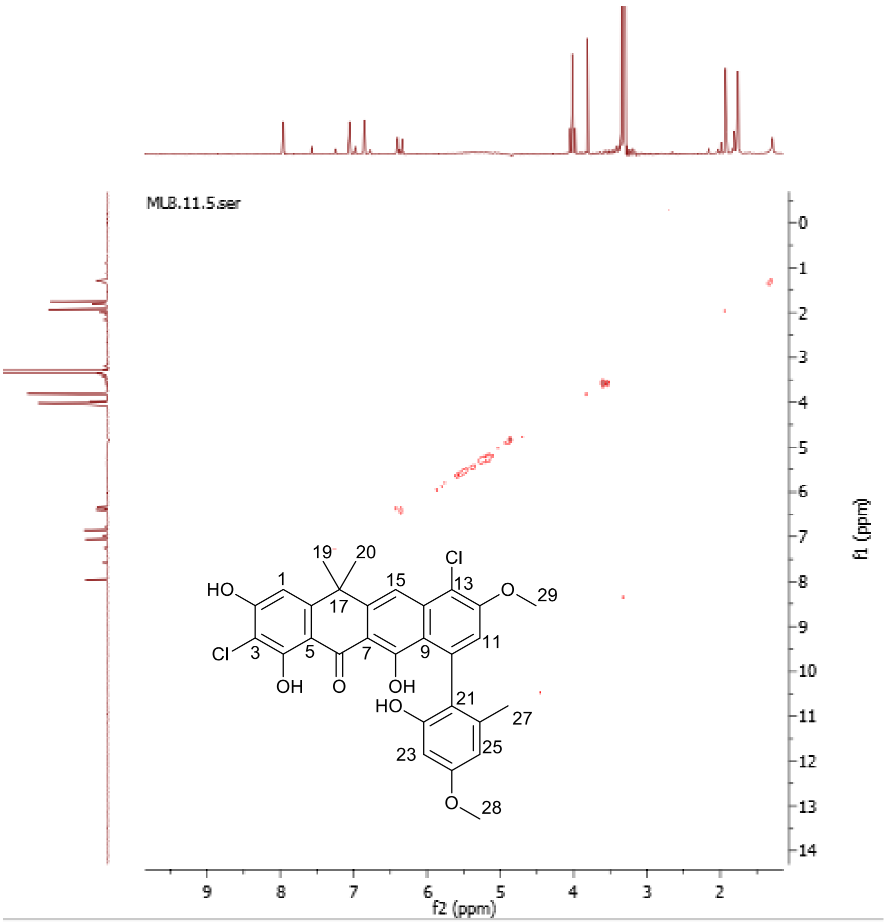

Figure S38. ^{1}H-^{1}H COSY Accramycin E **5** (CD_3OD, 298K, 600MHz)

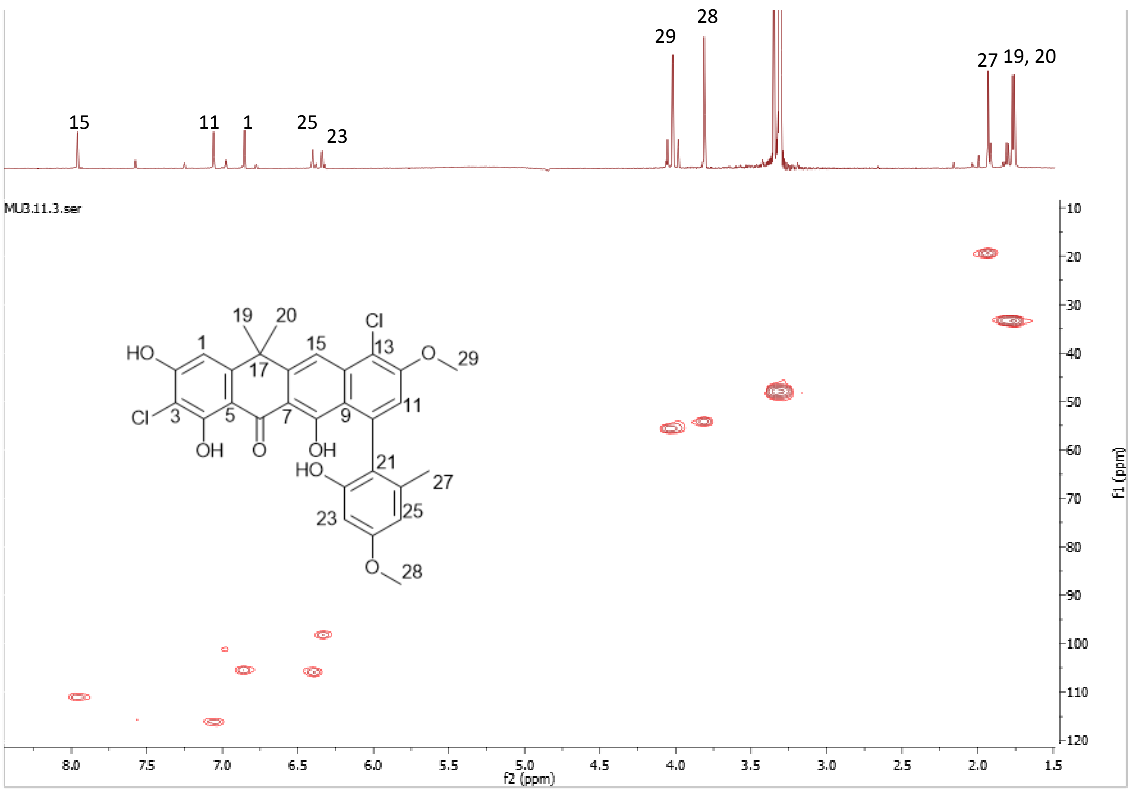

Figure S39. HSQC Accramycin E **5** (CD_3OD, 298K, 600MHz)

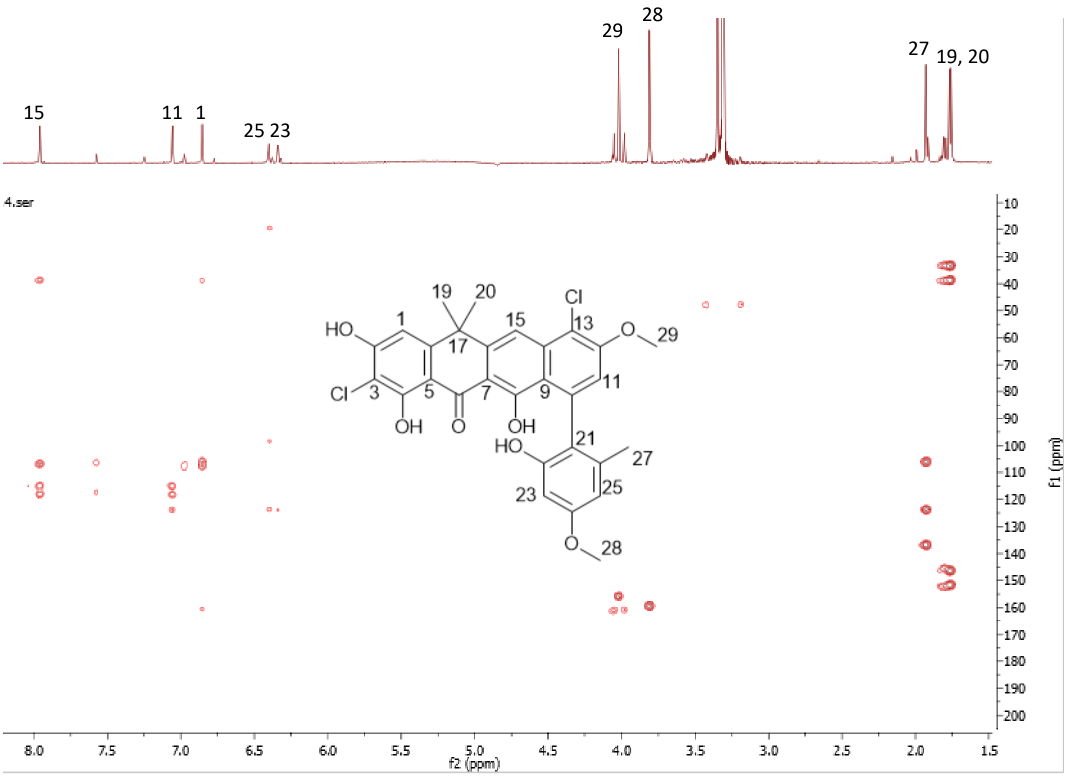

Figure S40. HMBC Accramycin E **5** (CD_3OD, 298K, 600MHz)

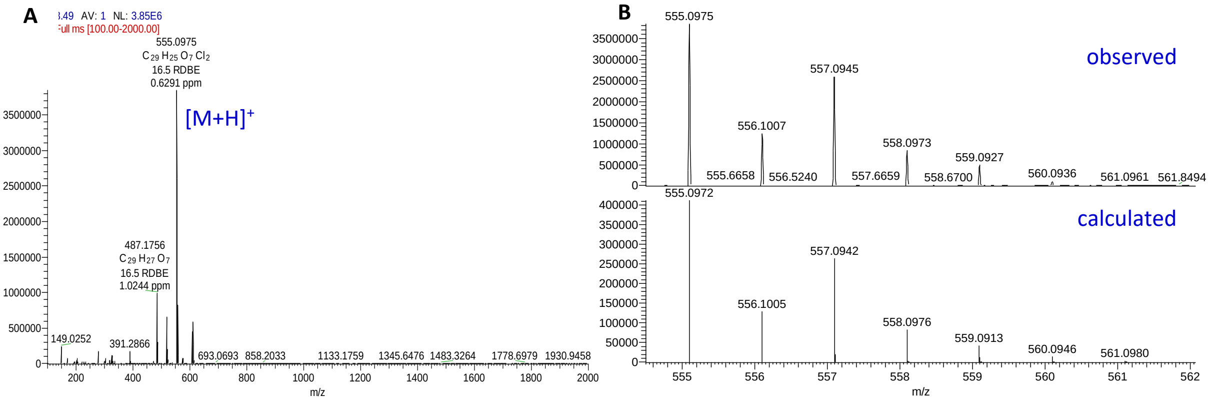

Figure S41. **A.** HRESIMS and **B.** Isotope Pattern of Accramycin F **6**

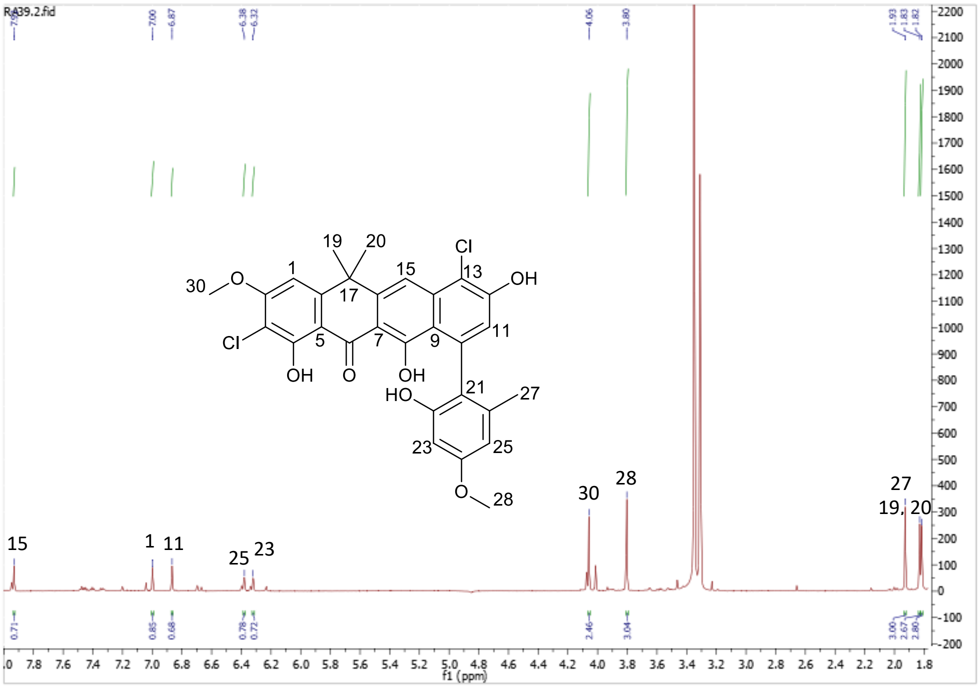

Figure S42. ^{1}H-NMR of Accramycin F **6** (CD_3OD, 298K, 600MHz)

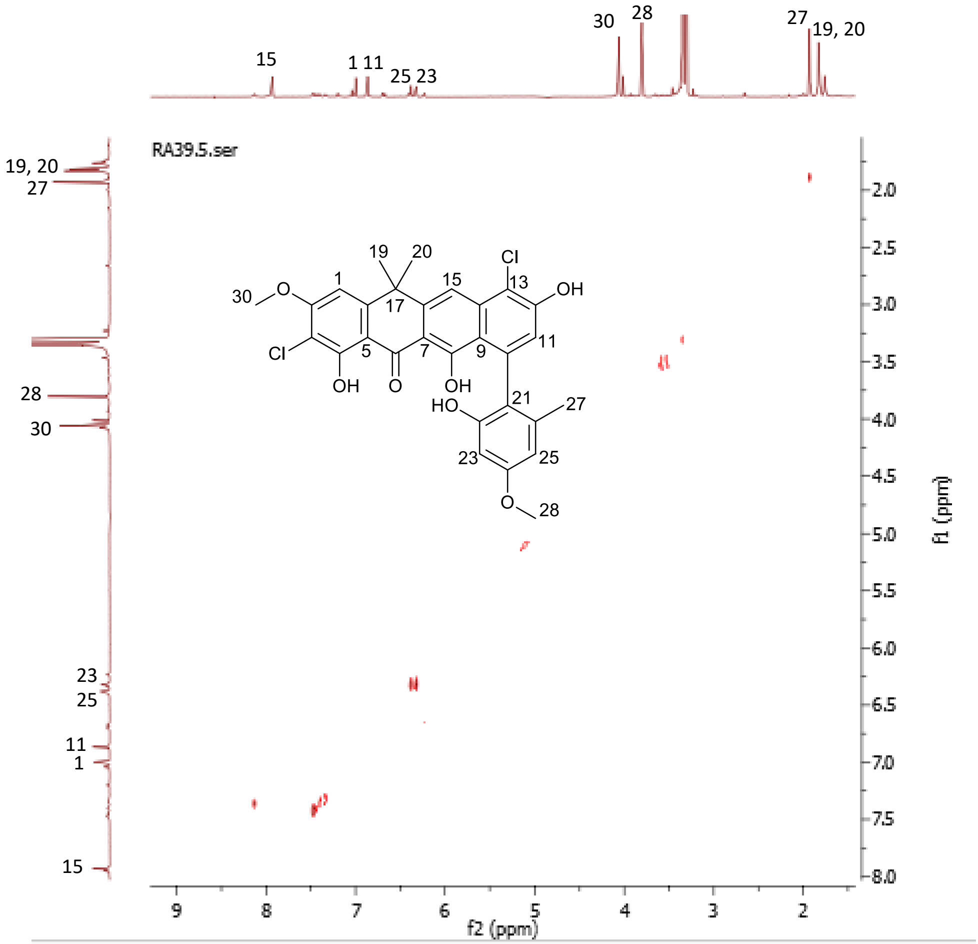

Figure S43. ^{1}H-^{1}H COSY of Accramycin F **6** (CD_3OD, 298K, 600MHz)

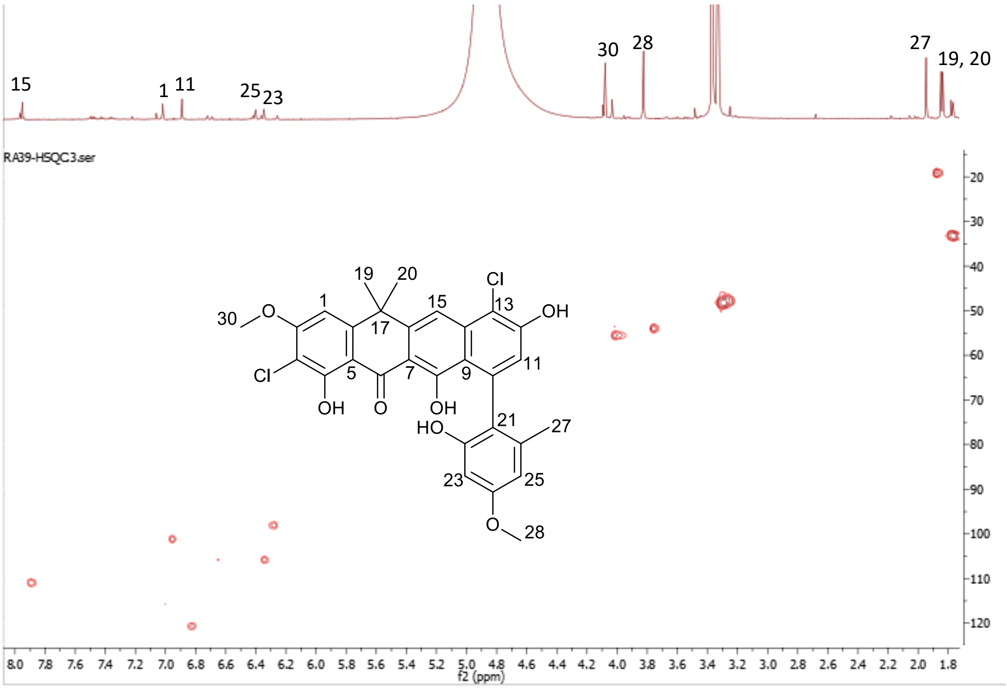

Figure S44. HSQC NMR of Accramycin F **6** (CD_3OD, 298K, 600MHz)

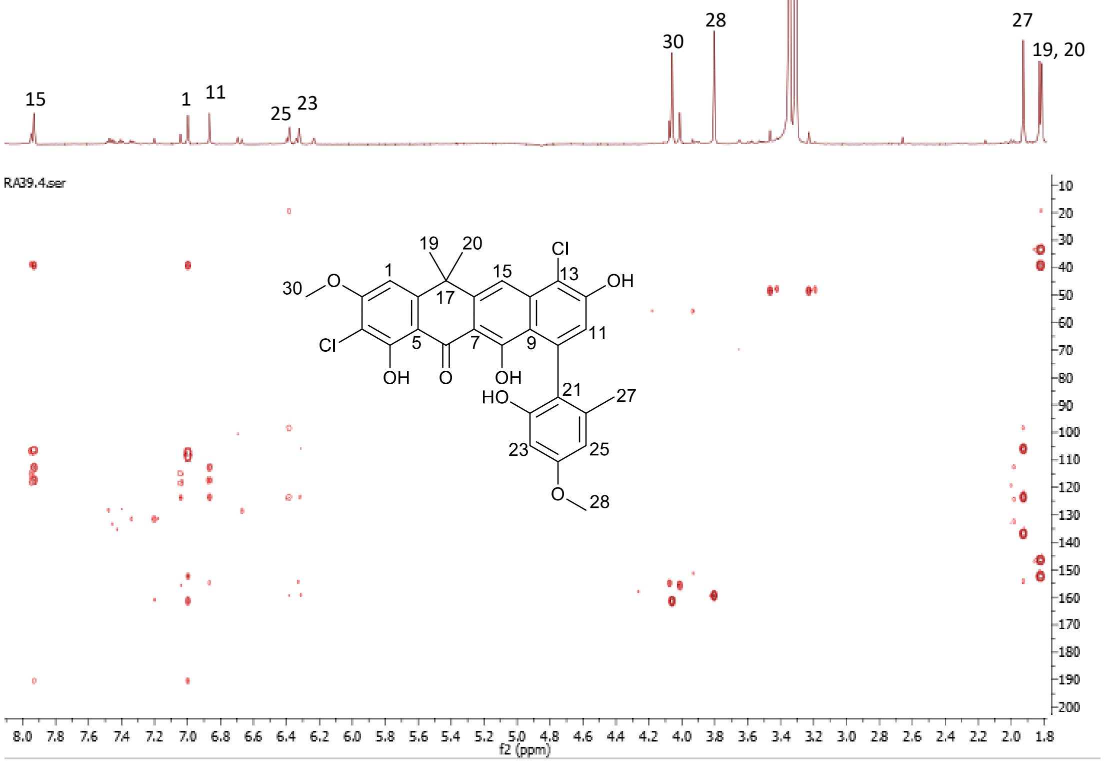

Figure S45. HMBC NMR of Accramycin F **6** (CD_3OD, 298K, 600MHz)

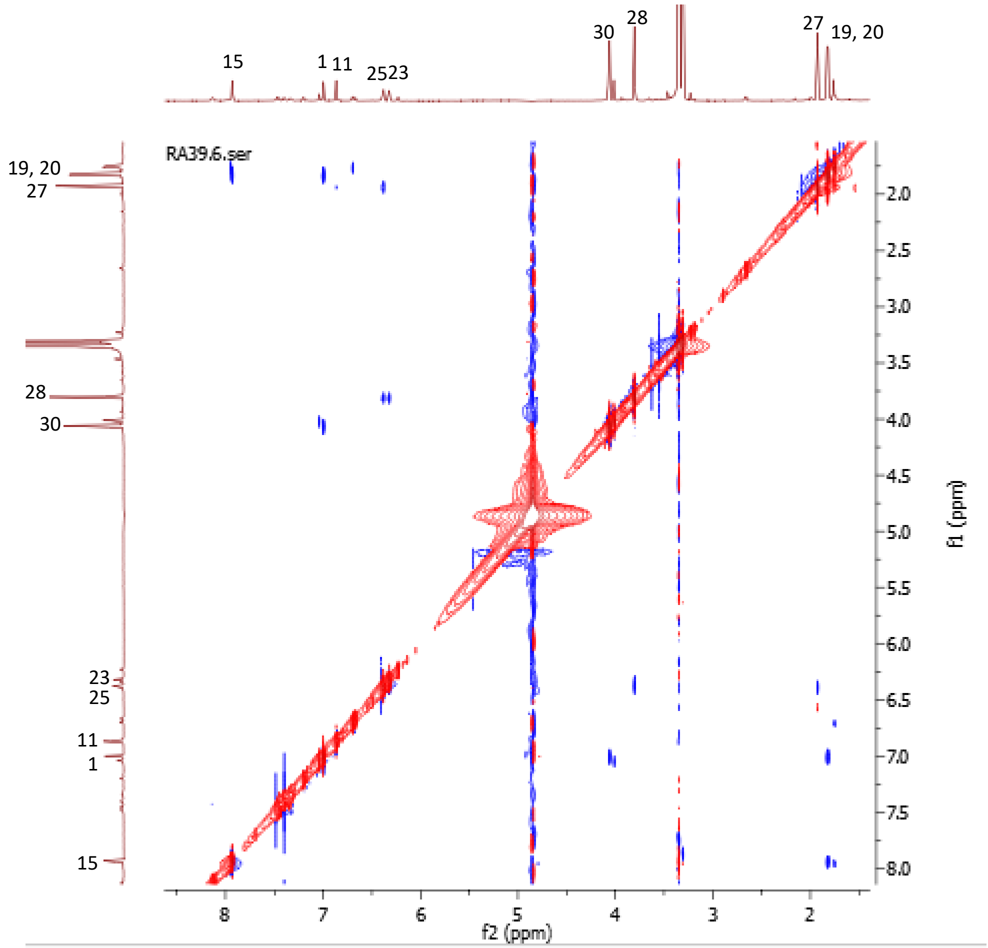

Figure S46. NOESY of Accramycin F **6** (CD_3OD, 298K, 600MHz)

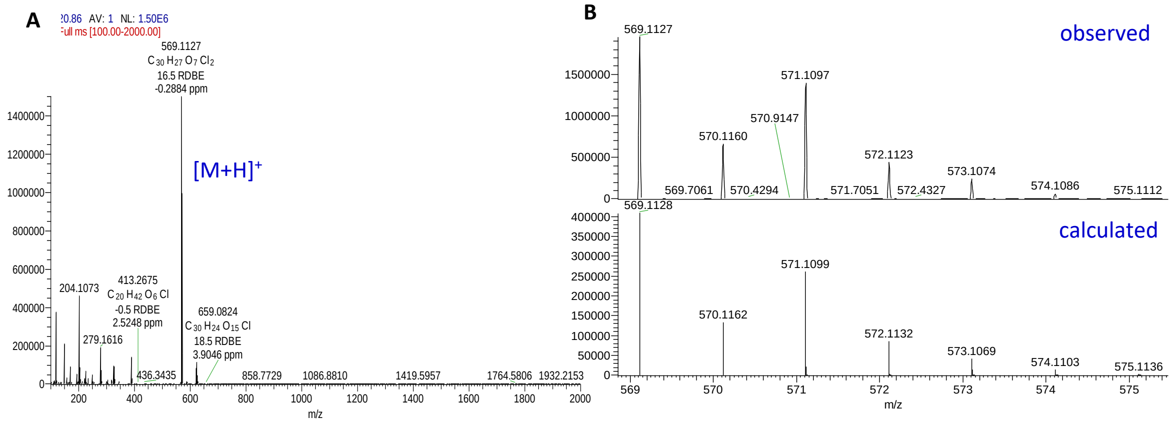

Figure S47. **A.** HRESIMS and **B.** Isotope Pattern of Accramycin G **7**

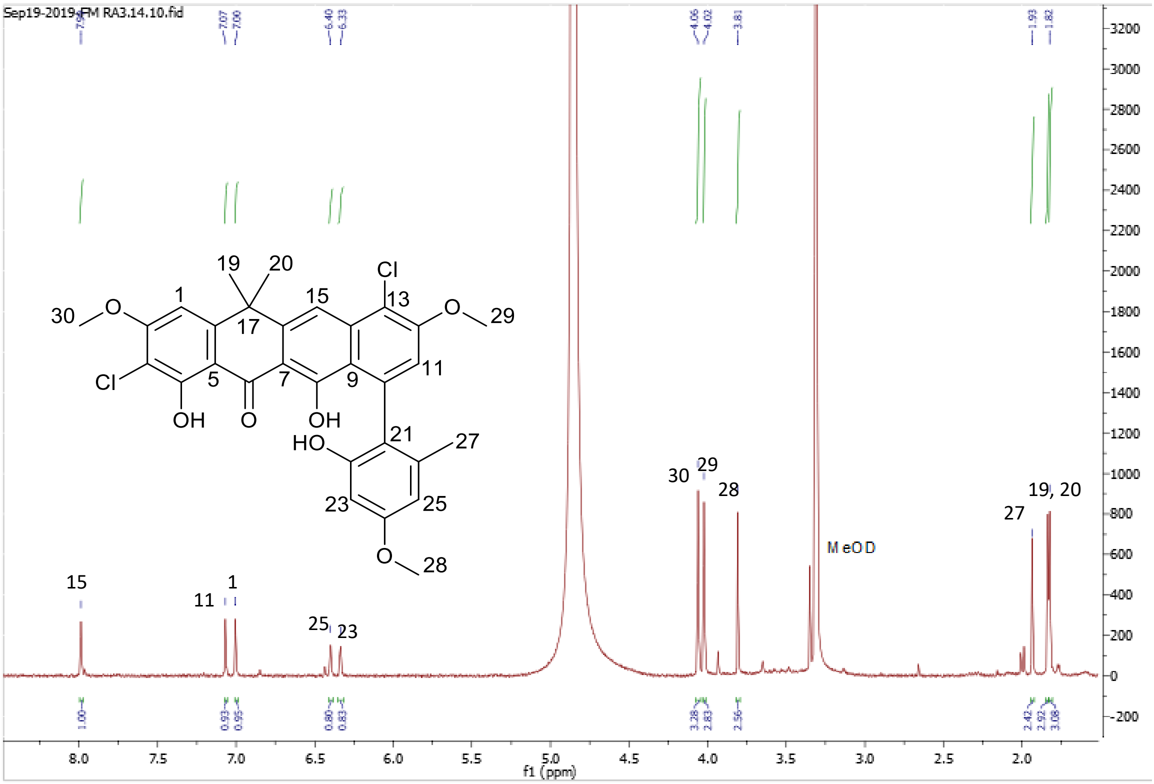

Figure S48. ^{1}H-NMR of Accramycin G **7** (CD_3OD, 298K, 600MHz)

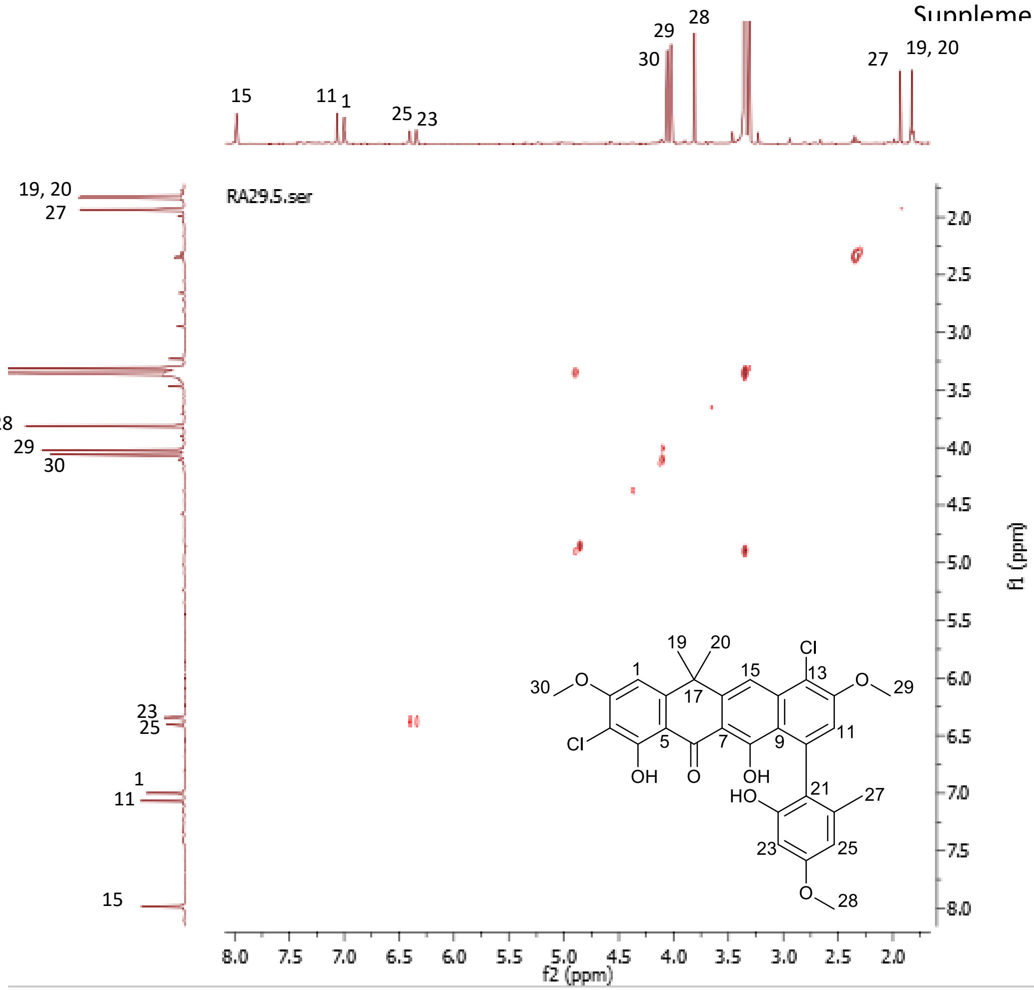

Figure S49. ^1H-^1H COSY of Accramycin G **7** (CD_3OD, 298K, 600MHz)

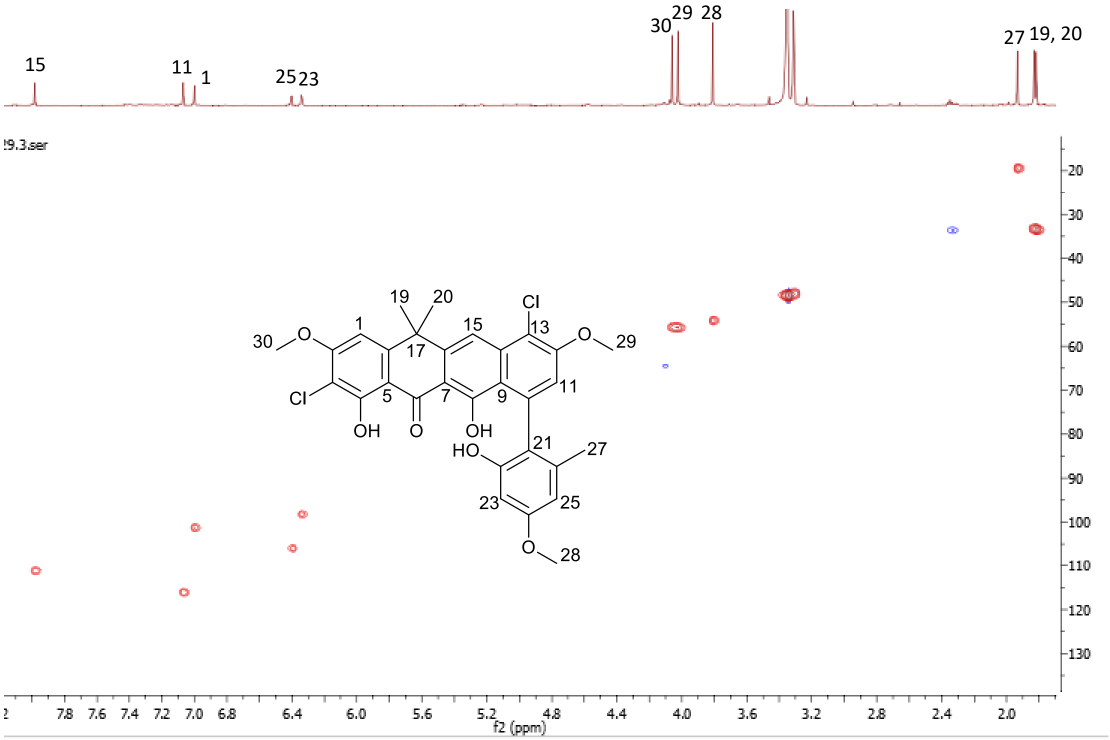

Figure S50. HSQC of Accramycin G **7** (CD_3OD, 298K, 600MHz)

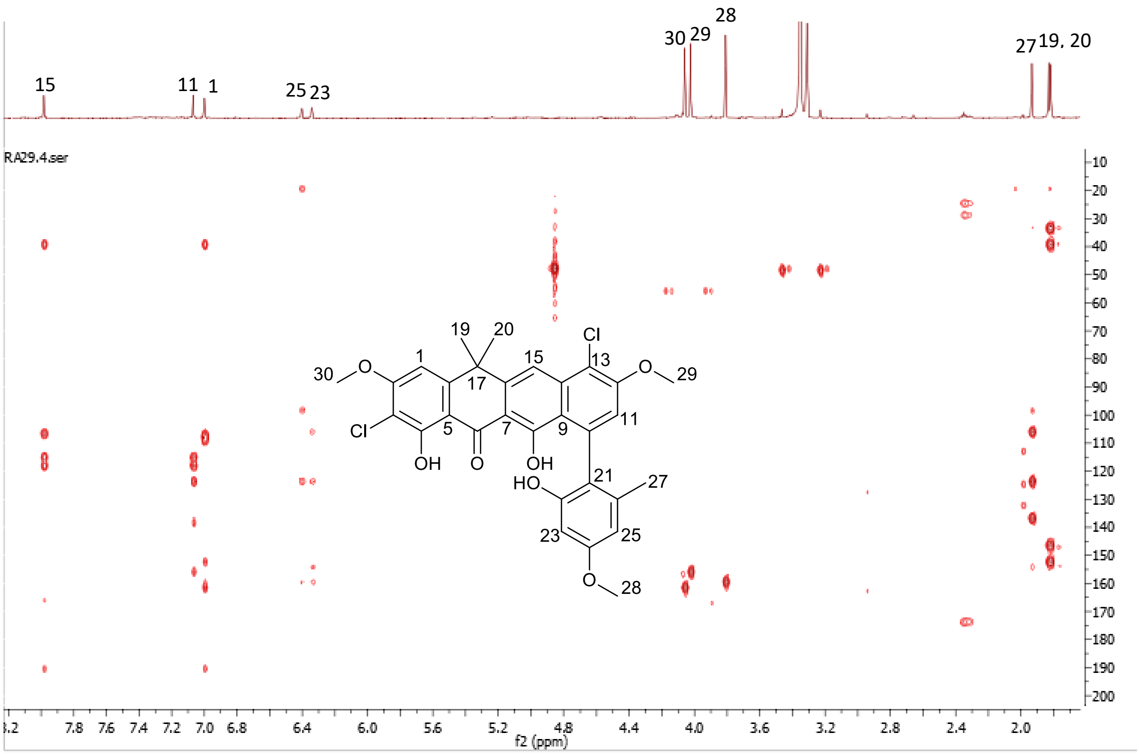

Figure S51. HMBC of Accramycin G **7** (CD_3OD, 298K, 600MHz)

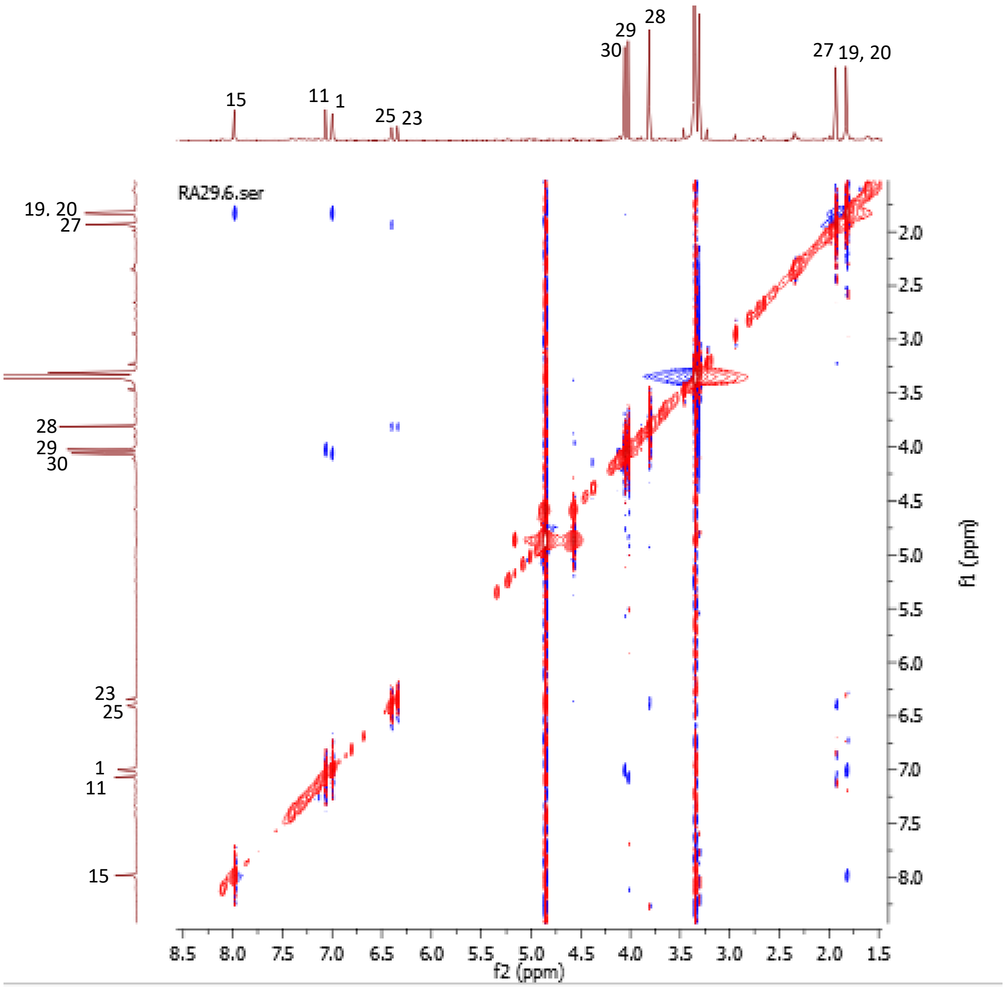

Figure S52. NOESY of Accramycin G **7** (CD_3OD, 298K, 600MHz)

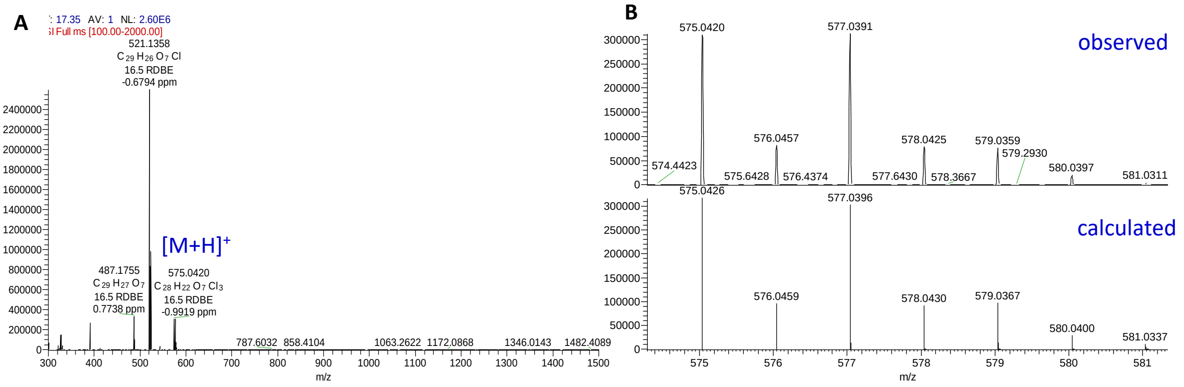

Figure S53. **A.** HRESIMS and **B.** Isotope Pattern of Accramycin H **8**

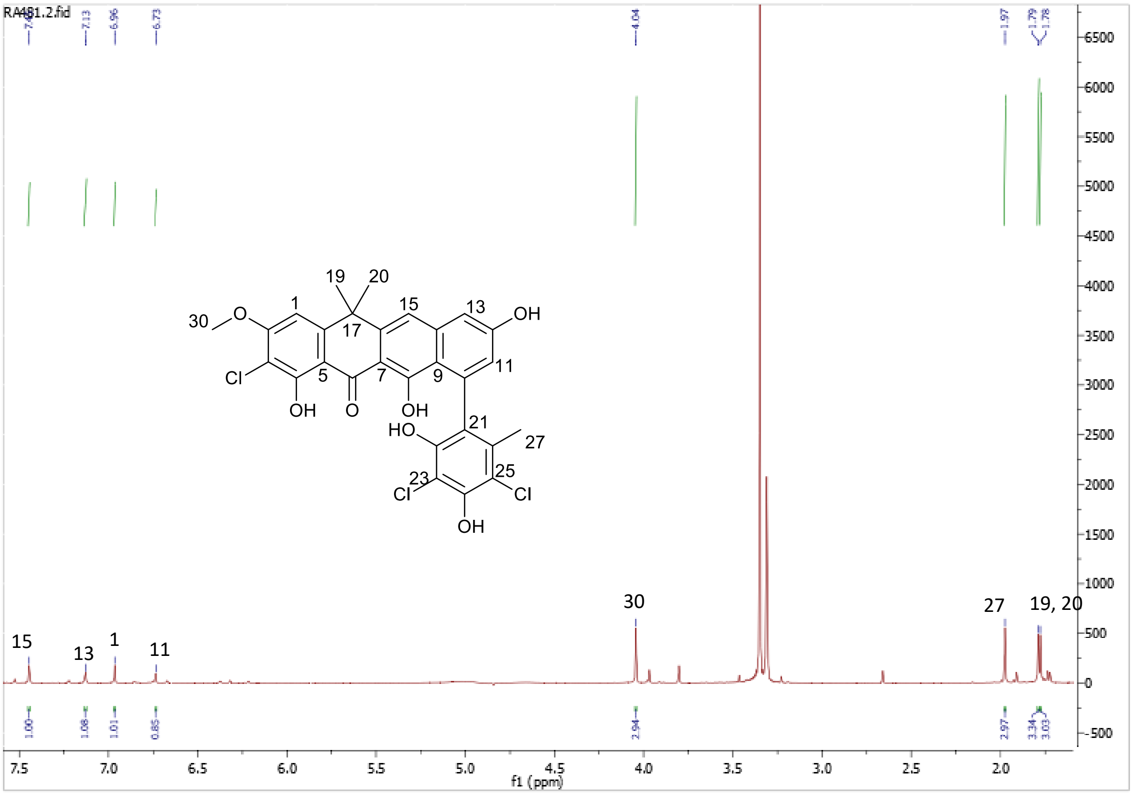

Figure S54. ^{1}H-NMR of Accramycin H **8** (CD_3OD, 298K, 600MHz)

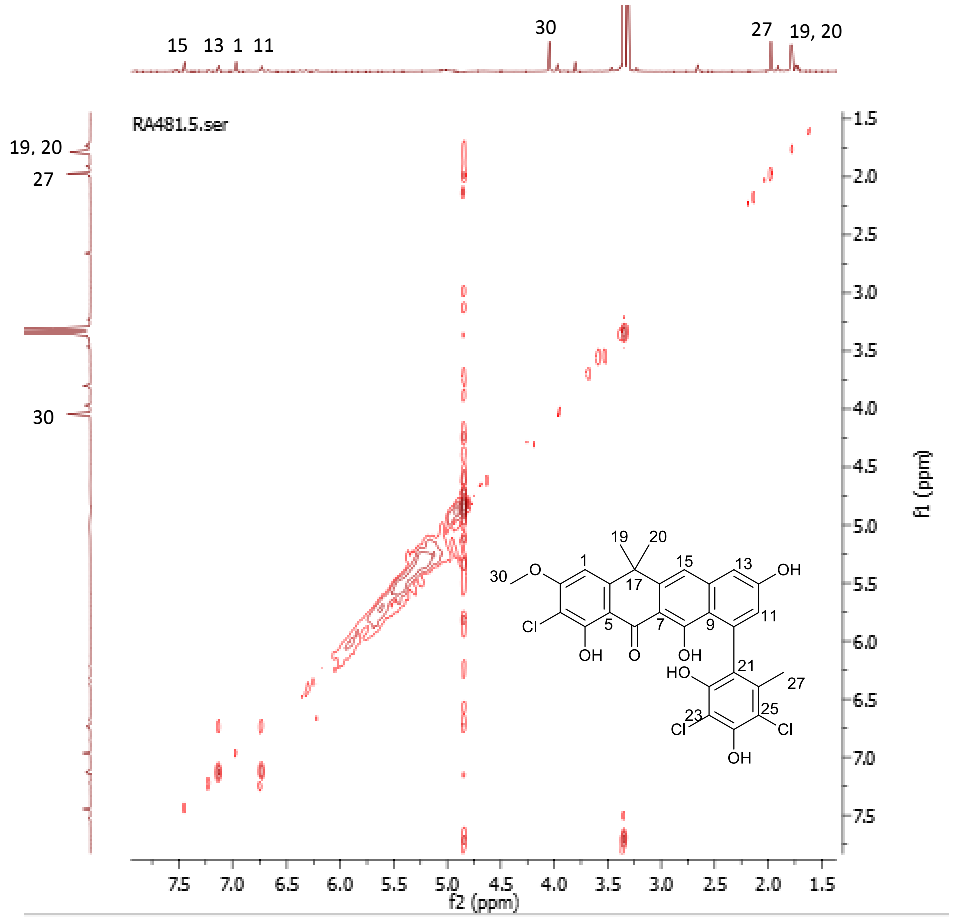

Figure S55. ^1H-^1H COSY of Accramycin H **8** (CD_3OD, 298K, 600MHz)

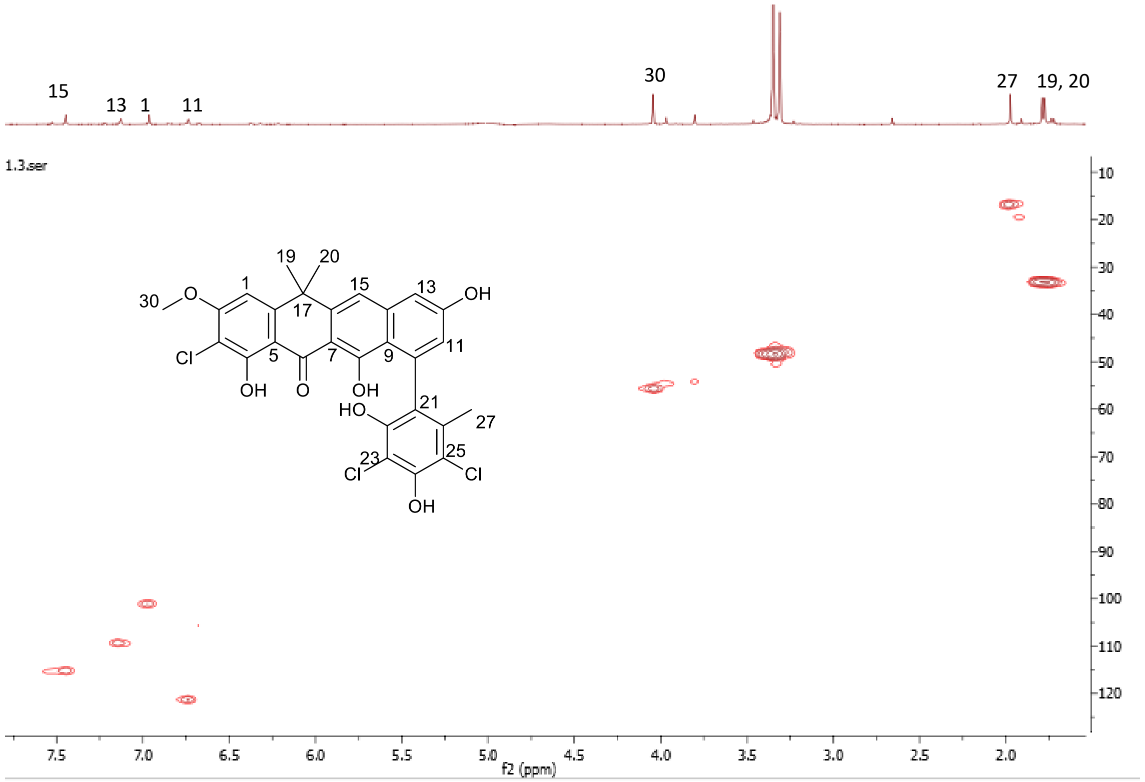

Figure S56. HSQC of Accramycin H **8** (CD_3OD, 298K, 600MHz)

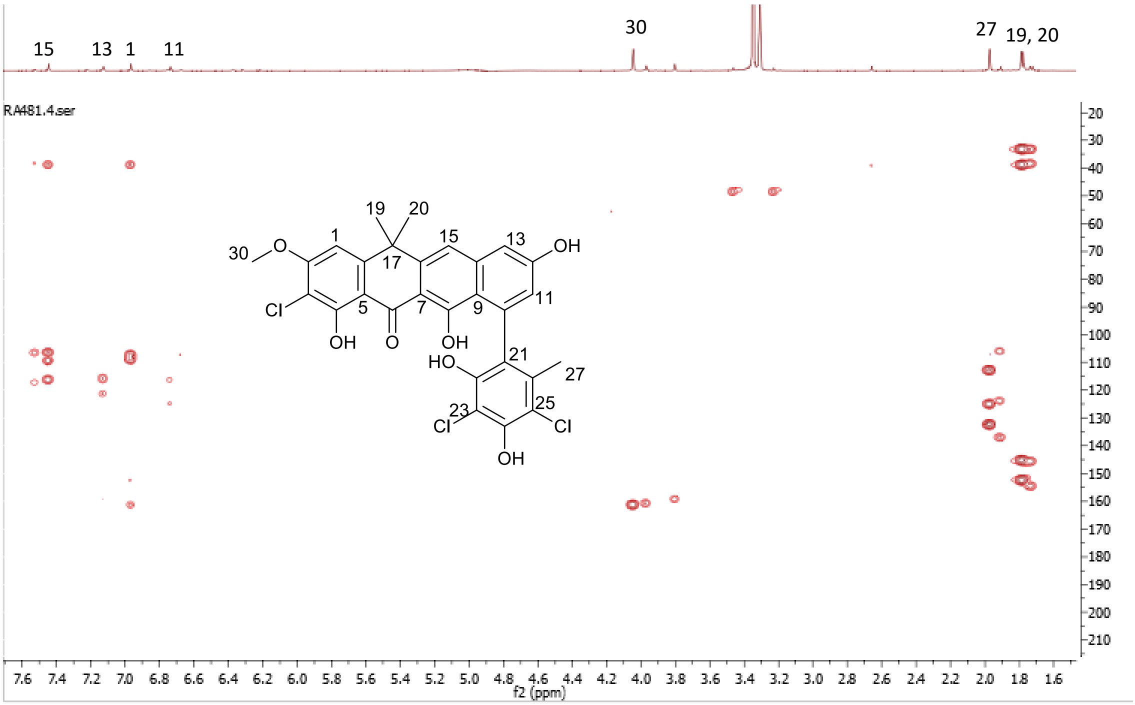

Figure S57. HMBC of Accramycin H **8** (CD_3OD, 298K, 600MHz)

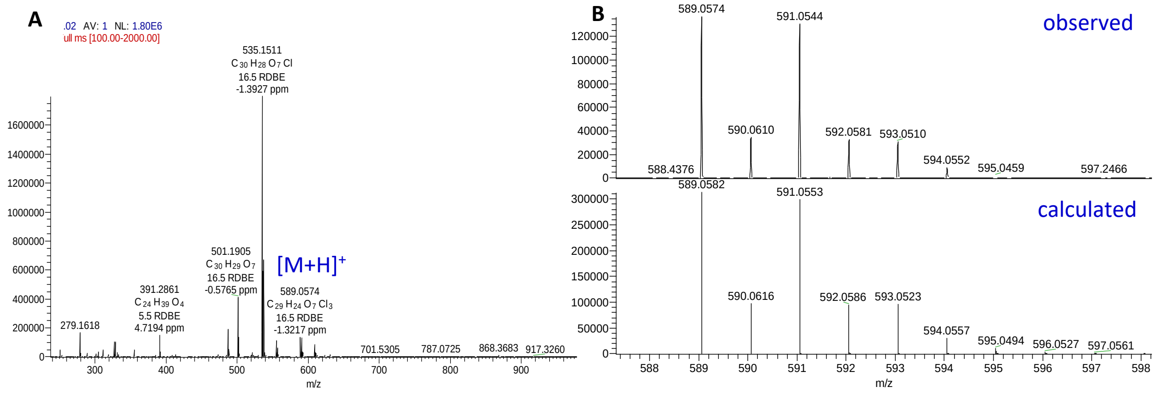

Figure S58. **A.** HRESIMS and **B.** Isotope Pattern of Accramycin I **9**

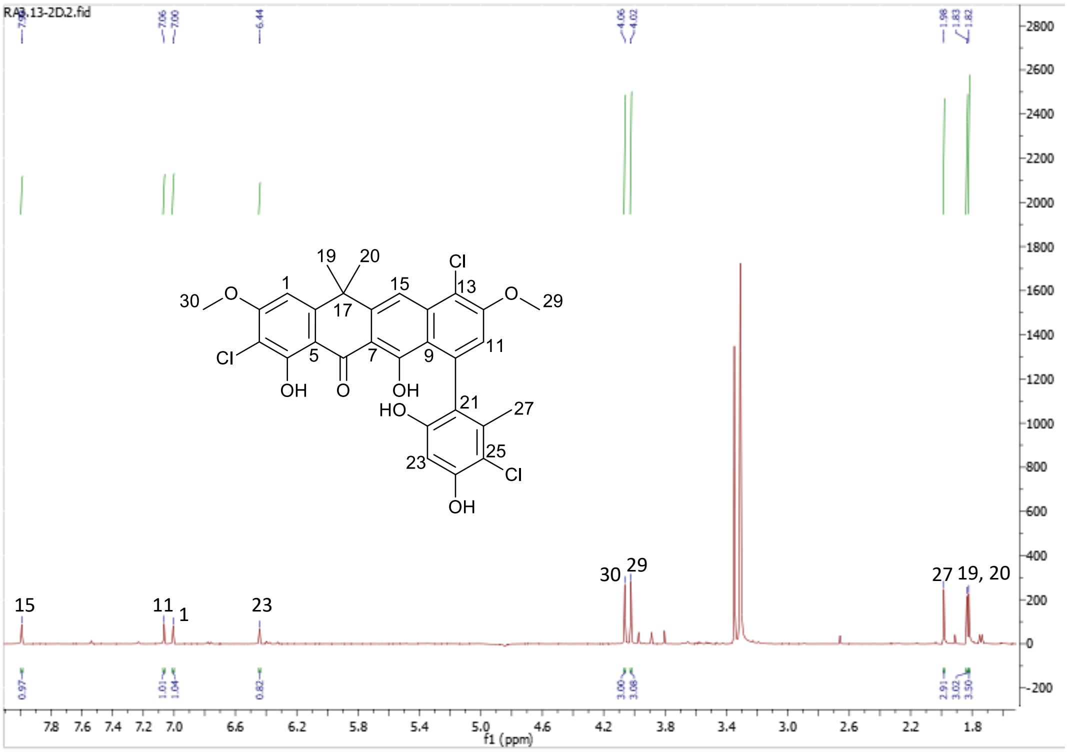

Figure S59. ^{1}H NMR of Accramycin I **9** (CD_3OD, 298K, 600MHz)

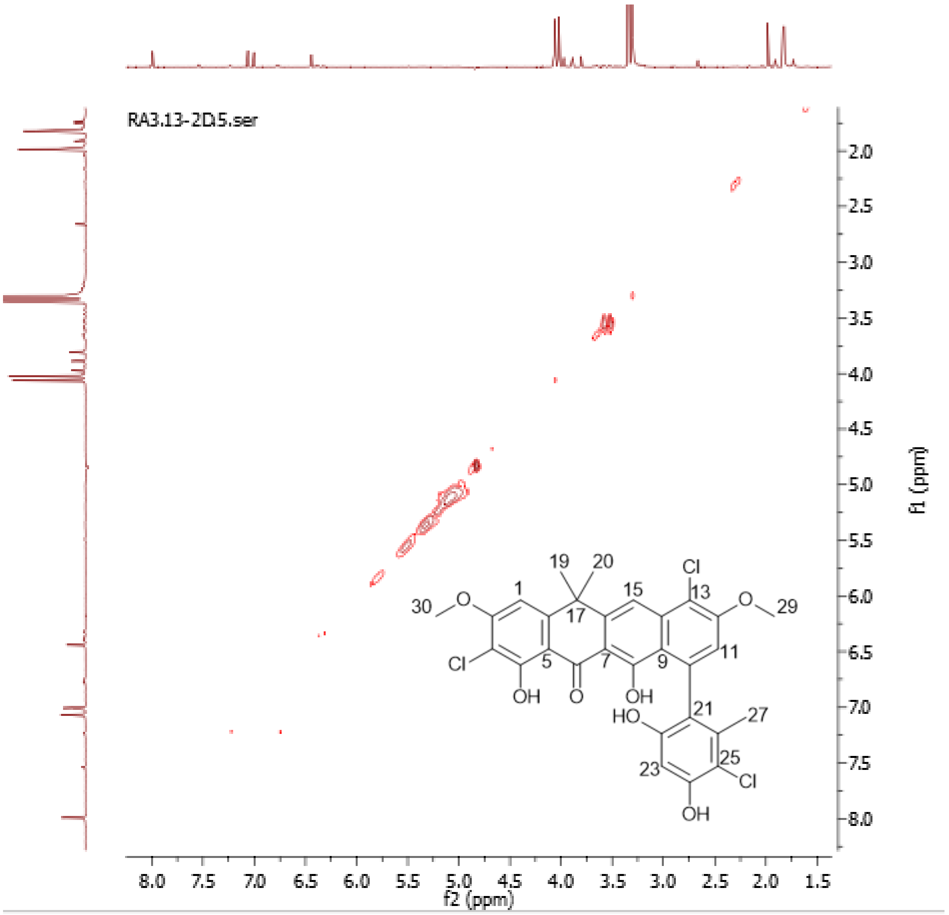

Figure S60. ^{1}H-^{1}H COSY of Accramycin I **9** (CD_3OD, 298K, 600MHz)

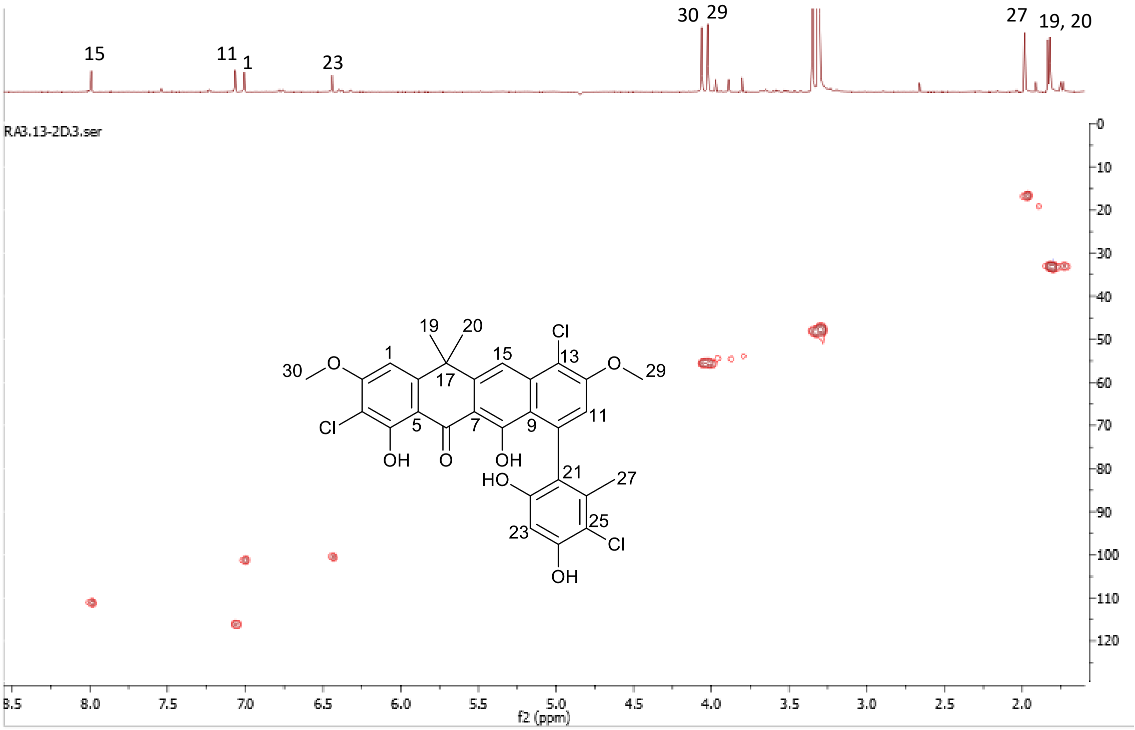

Figure S61. HSQC of Accramycin I **9** (CD_3OD, 298K, 600MHz)

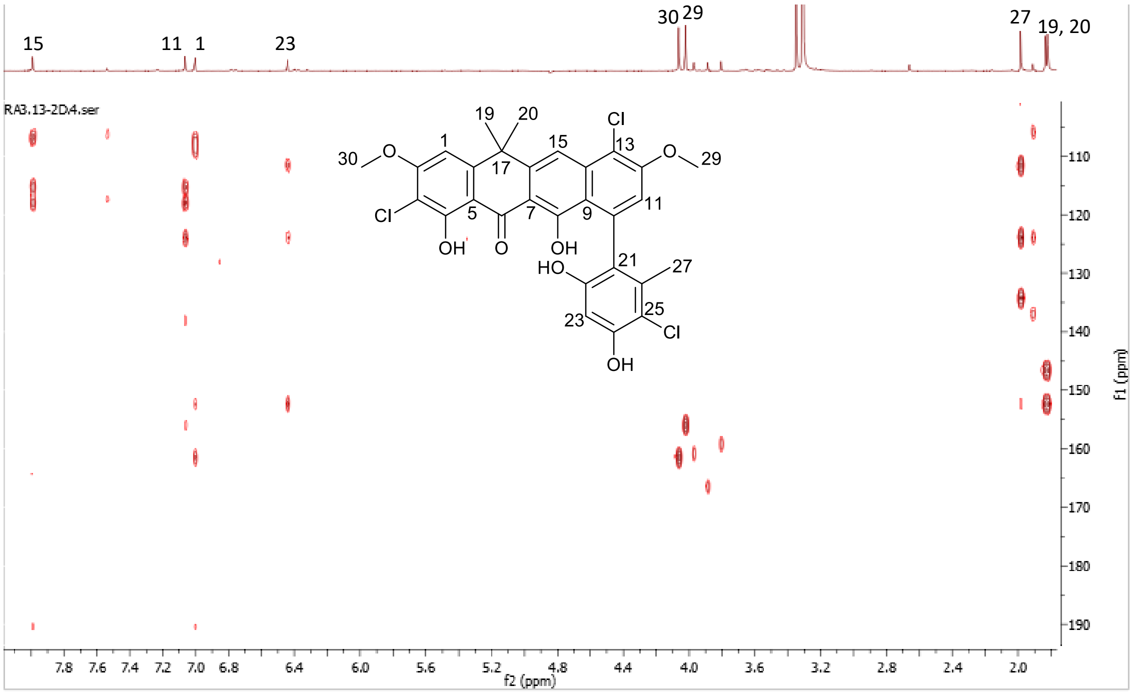

Figure S62. HMBC of Accramycin I **9** (CD_3OD, 298K, 600MHz)

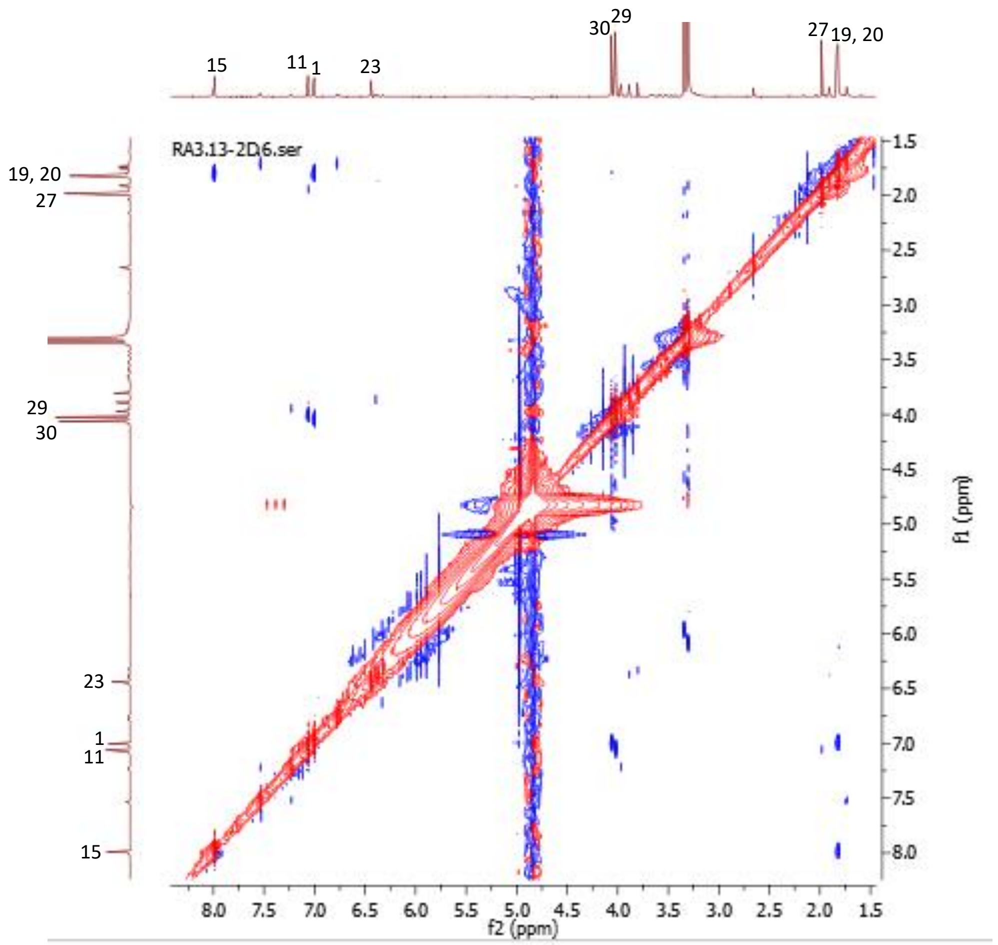

Figure S63. NOESY of Accramycin I **9** (CD_3OD, 298K, 600MHz)

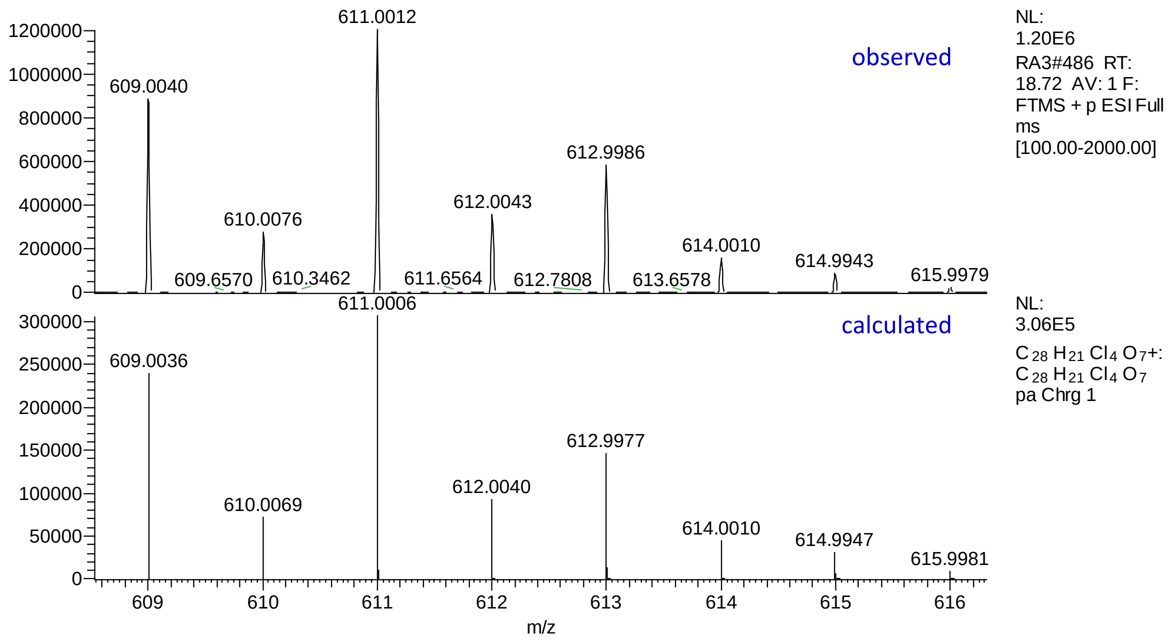

Figure S64. LCMS isotope pattern of Accramycin J **10**

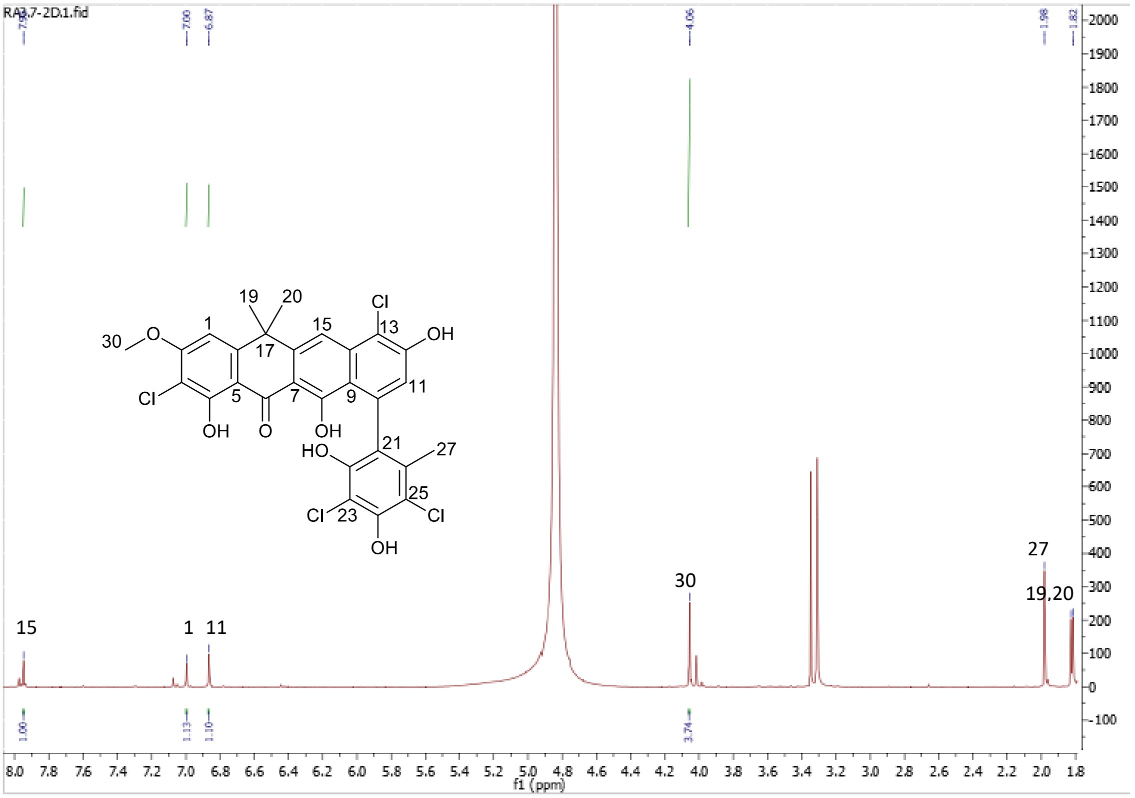

Figure S65. ^{1}H-NMR of Accramycin J **10** (CD_3OD, 298K, 600MHz)

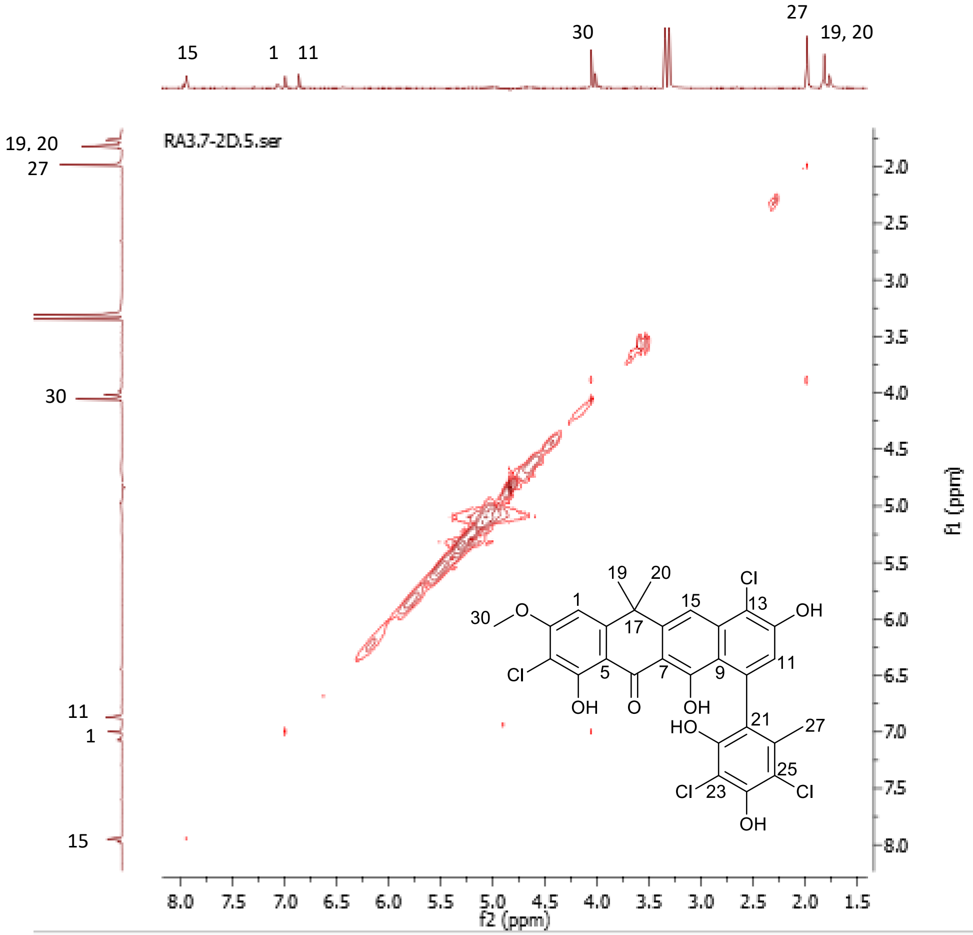

Figure S66. ^{1}H-^{1}H COSY of Accramycin J **10** (CD_3OD, 298K, 600MHz)

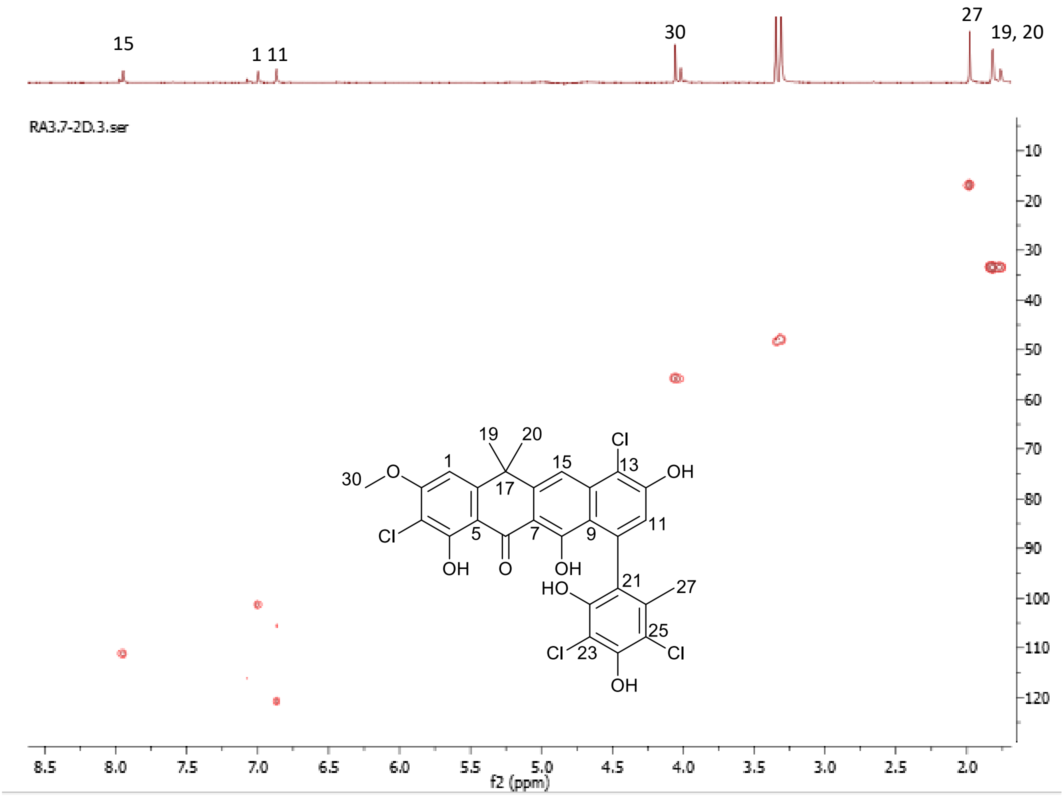

Figure S67. HSQC of Accramycin J **10** (CD_3OD, 298K, 600MHz)

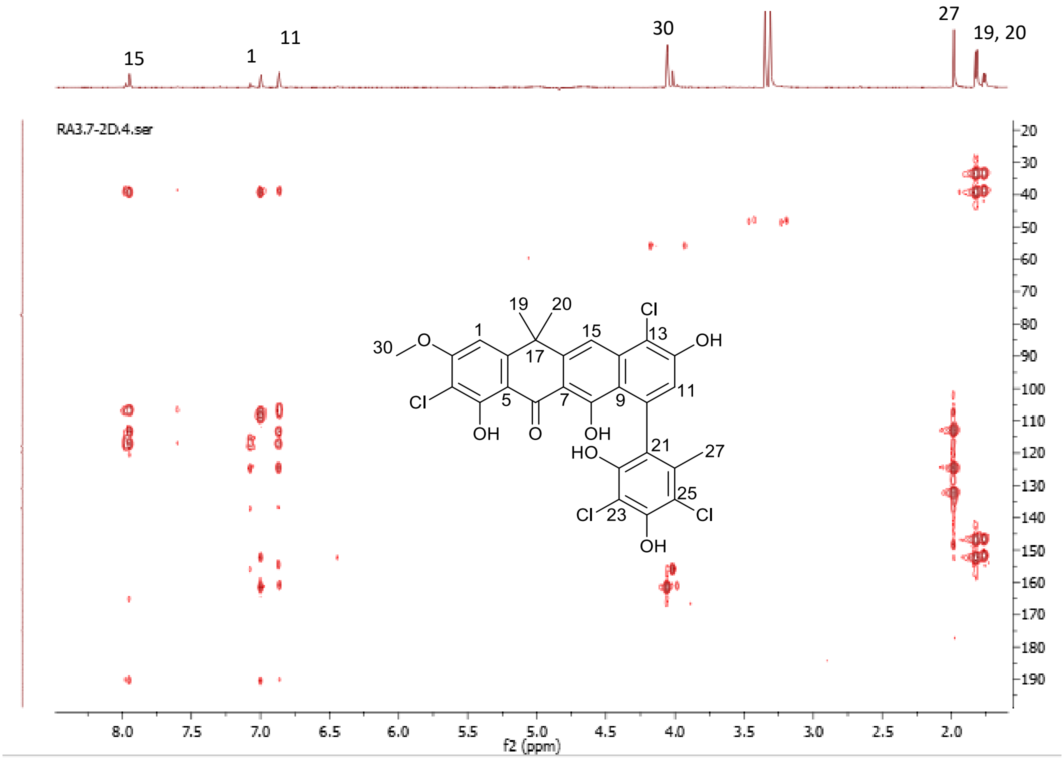

Figure S68. HMBC of Accramycin J **10** (CD_3OD, 298K, 600MHz)

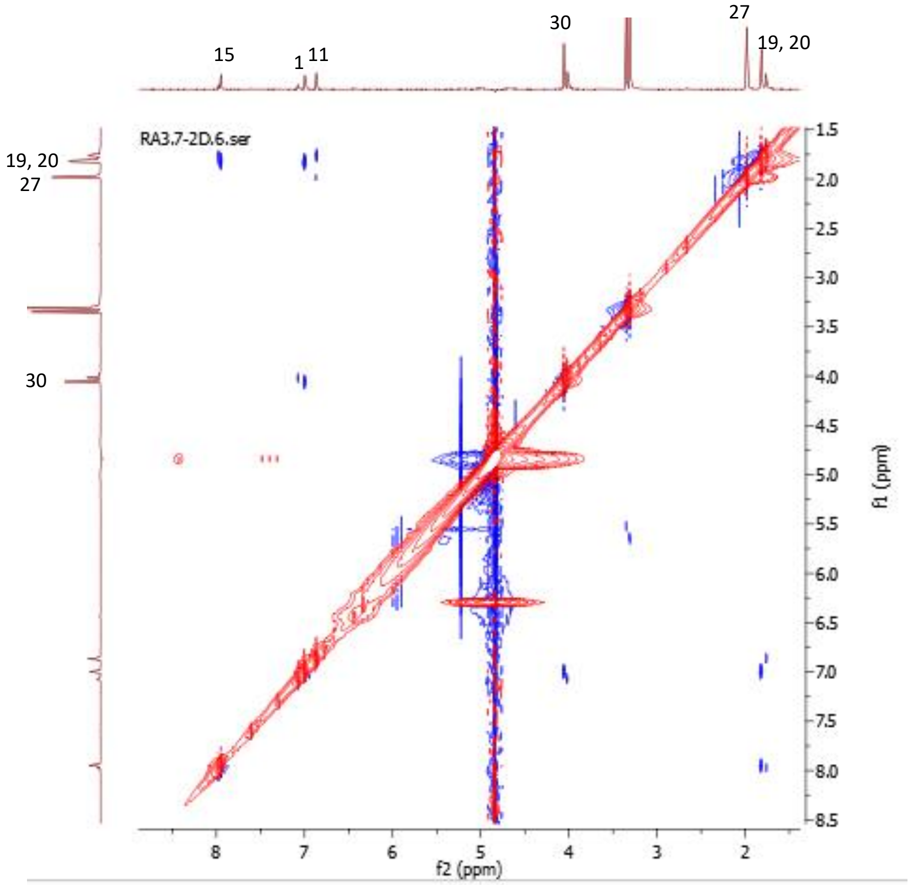

Figure S69. NOESY of Accramycin J **10** (CD_3OD, 298K, 600MHz)

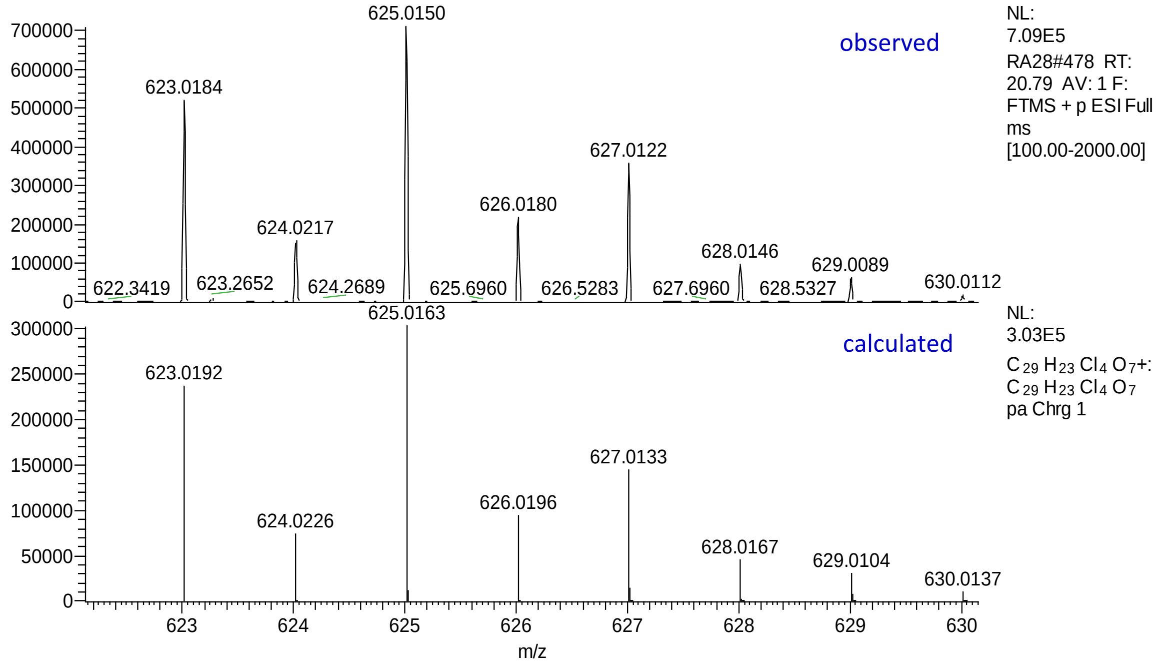

Figure S70. LCMS Isotope Pattern of Accramycin K **11**

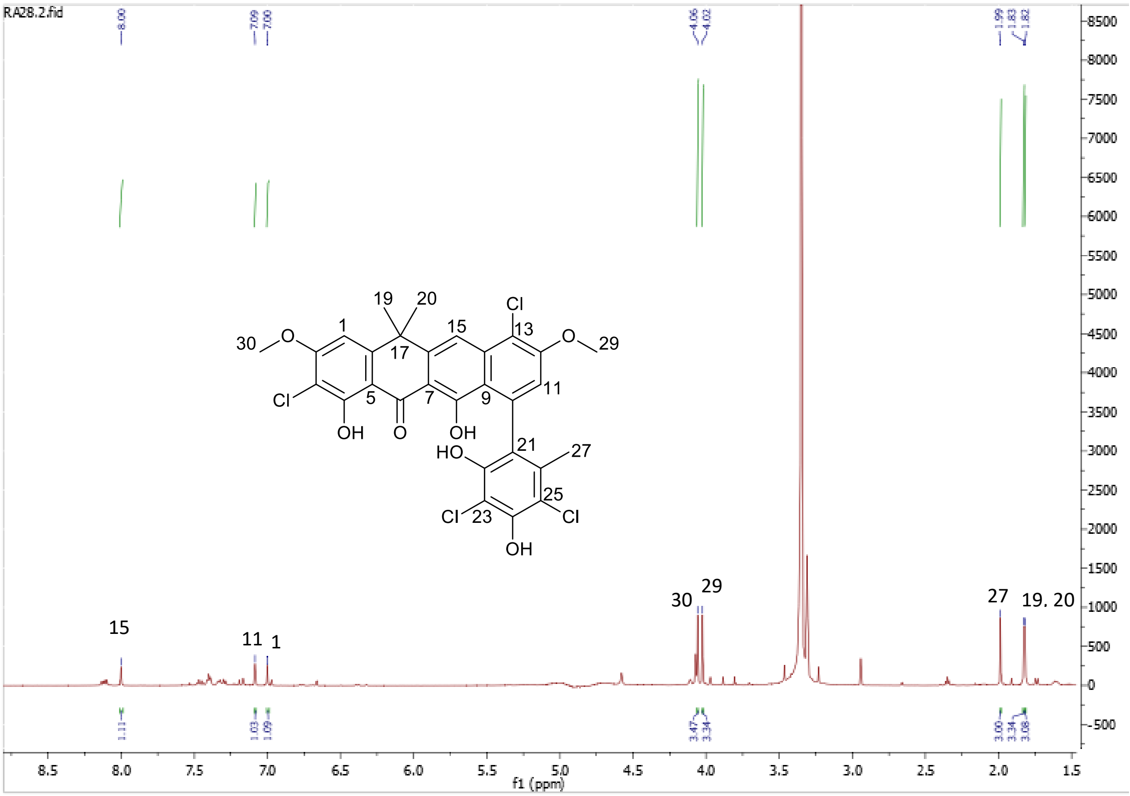

Figure S71. ^{1}H-NMR of Accramycin K **11** (CD_3OD, 298K, 600MHz)

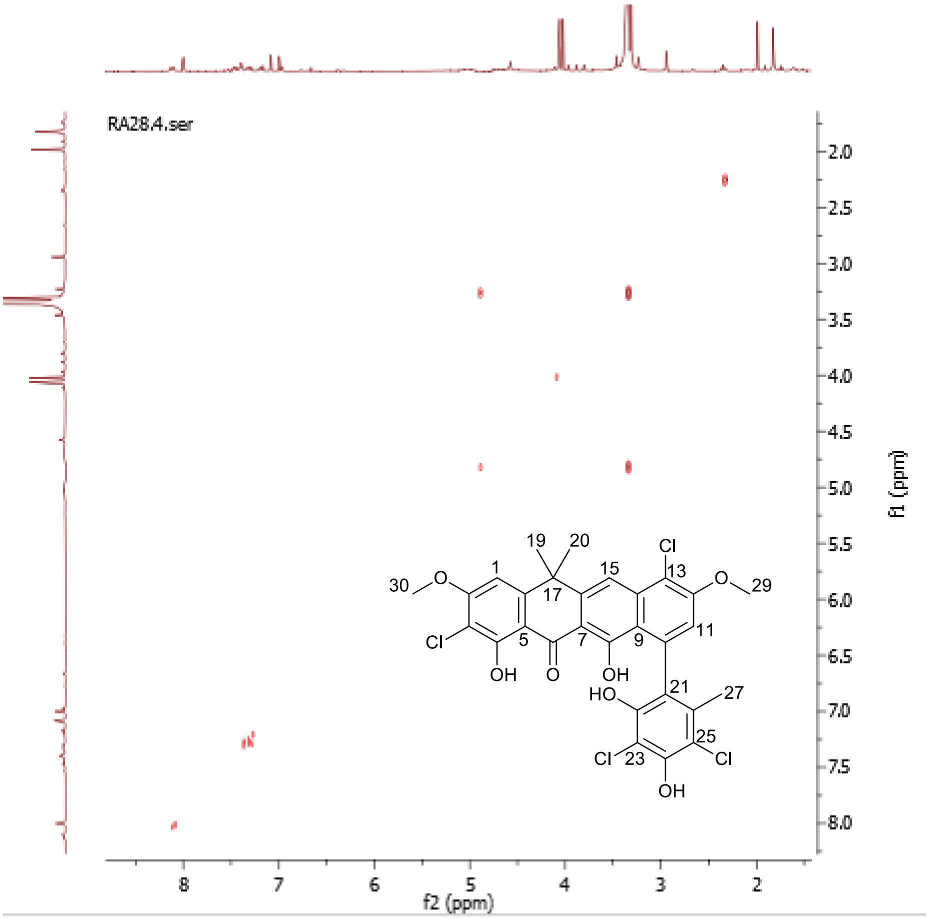

Figure S72. ^{1}H-^{1}H COSY of Accramycin K **11** (CD_3OD, 298K, 600MHz)

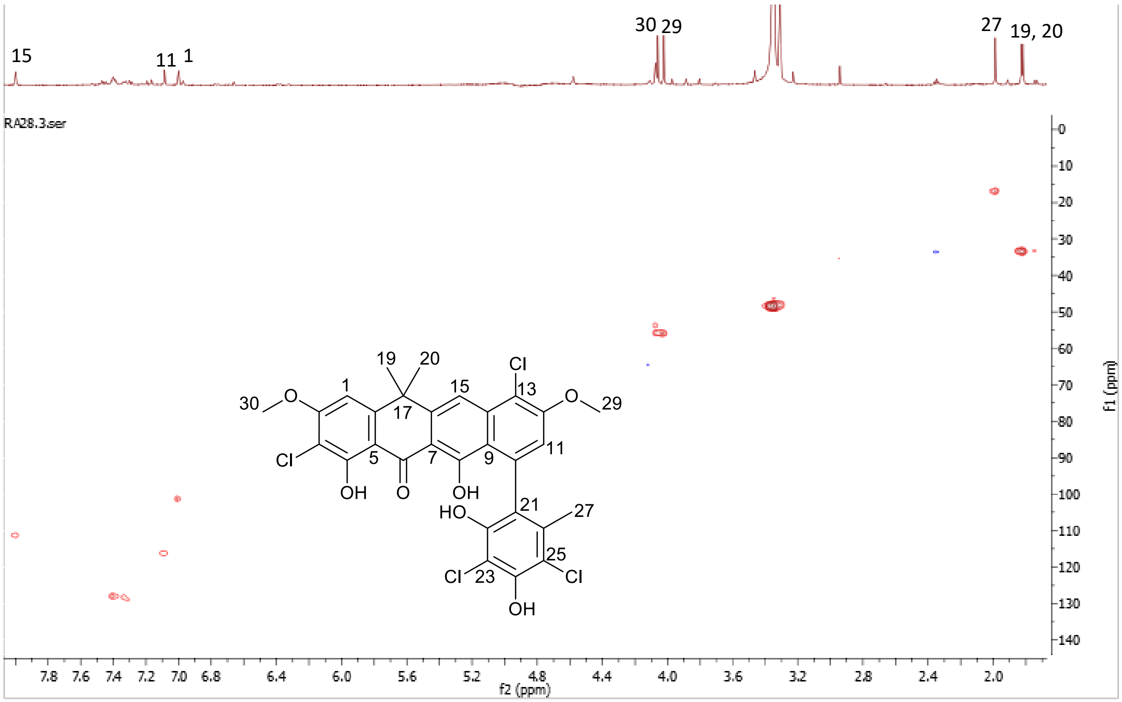

Figure S73. HSQC of Accramycin K **11** (CD_3OD, 298K, 600MHz)

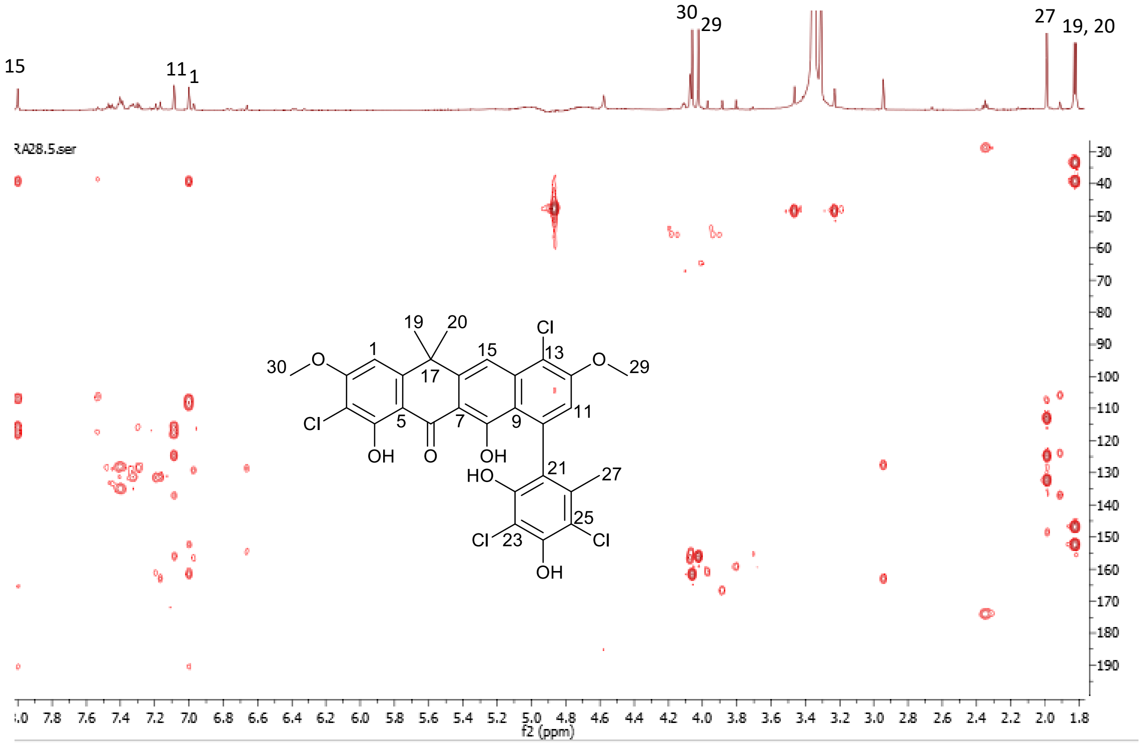

Figure S74. HMBC of Accramycin K **11** (CD_3OD, 298K, 600MHz)

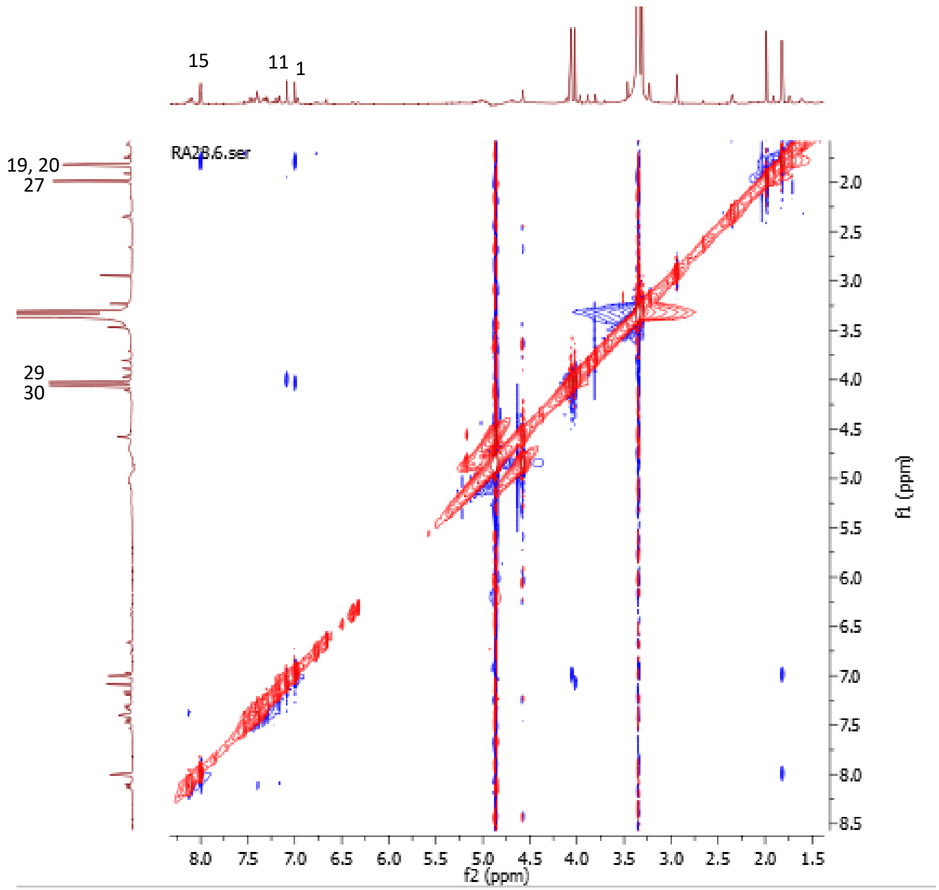

Figure S75. NOESY of Accramycin K **11** (CD_3OD, 298K, 600MHz)
